# Synthesis of 1,4-imino-L-lyxitols modified at C-5 and their evaluation as inhibitors of GH38 α-mannosidases

**DOI:** 10.3762/bjoc.14.189

**Published:** 2018-08-17

**Authors:** Maroš Bella, Sergej Šesták, Ján Moncoľ, Miroslav Koóš, Monika Poláková

**Affiliations:** 1 Department of Glycochemistry, Institute of Chemistry, Slovak Academy of Sciences, Dúbravská cesta 9, SK-845 38, Bratislava, Slovakia; 2 Department of Inorganic Chemistry, Faculty of Chemical and Food Technology, Radlinského 9, SK-812 37 Bratislava, Slovakia

**Keywords:** azasugars, hydrolases, inhibitors, pyrrolidines, synthesis

## Abstract

A synthetic approach to 1,4-imino-L-lyxitols with various modifications at the C-5 position is reported. These imino-L-lyxitol cores were used for the preparation of a series of *N*-(4-halobenzyl)polyhydroxypyrrolidines. An impact of the C-5 modification on the inhibition and selectivity against GH38 α-mannosidases from *Drosophila melanogaster*, the Golgi (GMIIb) and lysosomal (LManII) mannosidases and commercial jack bean α-mannosidase from *Canavalia ensiformis* was evaluated. The modification at C-5 affected their inhibitory activity against the target GMIIb enzyme. In contrast, no inhibition effect of the pyrrolidines against LManII was observed. The modification of the imino-L-lyxitol core is therefore a suitable motif for the design of inhibitors with desired selectivity against the target GMIIb enzyme.

## Introduction

Carbohydrates as chiral templates for a construction of bioactive compounds are of steady interest in medicinal chemistry [1–3]. The polyfunctional nature of carbohydrate units offers many possibilities for the design of a wide variety of new compounds. Moreover, various desired substituents can be selectively appended to any required position of the carbohydrate unit. This leads to a preparation of mimetics that meet the requirements of metabolically more stable bioactive compounds.

During the years, many scaffolds based on monosaccharides [4], disaccharides or higher oligosaccharides [5–6] as well as multivalent [7–8] carbohydrate units have been developed. These glycomimetics and glycopeptides have also found applications as bioactive compounds [9–10].

One group of the scaffolds includes iminosugars [11–12] as analogues of the monosaccharides wherein the endocyclic oxygen atom is replaced by a nitrogen atom. An additional feature of iminosugars, in comparison with their parent oxygen containing counterparts, is a protonation of the ring nitrogen under physiological pH. The protonation of the amine is often important for the inhibition properties of these compounds [13–14]. Another advantage of the iminosugars is a possibility to introduce a functional group onto the nitrogen atom. The importance of iminosugars is documented by a number of reports dealing with the synthesis of polyhydroxylated piperidines, pyrrolidines, pyrolizidines, indolizidines etc. which exhibited remarkable biological activities and are highly interesting as pharmaceutical agents [15–19]. In addition, iminosugars exhibited a powerful inhibitory activity against a wide range of glycoside hydrolases [20–22].

One naturally occurring iminosugar, alkaloid swainsonine, interferes with the glycosylation pathway where it specifically inhibits GH38 glycoside hydrolases [23–24]. Up to date, swainsonine is the most potent Golgi mannosidase II (GMII) inhibitor. It is known that inhibition of the biosynthesis of complex *N*-glycans in the Golgi apparatus influences progress of tumor growth and metastasis. However, all potent GMII inhibitors, including swainsonine, showed also an undesired co-inhibition of lysosomal α-mannosidase (LM) that limits their use as therapeutic agents [25]. Since the discovery of swainsonine, new inhibitors of GMII that are based on its structure and related pyrrolidines are of particular interest as potential candidates for cancer treatment [26–27].

Our research interest has been focused on searching for efficient inhibitors of α-mannosidases from the GH38 family. Another important feature required for such a potential inhibitor is its ability to inhibit only selected α-mannosidases, i.e., to exhibit high inhibition activity and selectivity against the enzymes within the GH38 family or even within the whole GH family. These enzymes were represented by model GH38 α-mannosidases from *Drosophila melanogaster* Golgi α-mannosidase II (GMIIb, target enzyme) and lysosomal α-mannosidase (LManII, enzyme not to be inhibited), and commercial enzyme jack bean α-mannosidase (JBMan) from *Canavalia ensiformis*.

**Figure 1:**
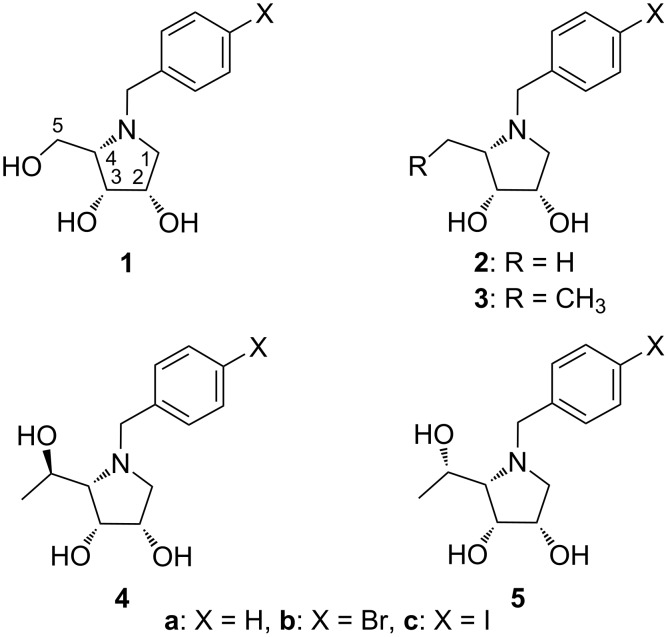
Structures of GMIIb inhibitors.

In a series of our previous papers, it has been found that a combination of a saccharide core (D-mannose, D-mannose with modification at C-6) and hydrophobic linker (benzyl, phenethyl) has an impact on the inhibition efficiency of the tested synthetic compound against the target GMIIb enzyme. Evaluation of these derivatives revealed that benzyl is a suitable hydrophobic linker. Some of them showed a weak inhibitory activity and selectivity against GMIIb [28–29]. Therefore, the structural design was further developed and a modification of the saccharide core, i.e., a replacement of the D-mannose unit to five-membered imino-L-lyxitol core has been suggested. Indeed, such *N*-benzyl-substituted polyhydroxypyrrolidines **1** (Figure 1) were found to be more potent and selective inhibitors of the target enzyme. Moreover, proposed *N*-benzyl substituent at the pyrrolidine core was also confirmed to be essential for selectivity [30].

In this study, we explored structure–activity relationships (SAR) of pyrrolidine derivatives **1** with different modifications at position 5. The benzyl moiety was chosen to be (4-halo)benzyl as the pyrrolidines **1** bearing these structural fragments were the most effective and selective against GMIIb [30]. The synthesis of modified pyrrolidine derivatives **2**–**5** (Figure 1) and GH38 α-mannosidases inhibition studies are reported.

## Results and Discussion

### Synthesis

**Scheme 1:**
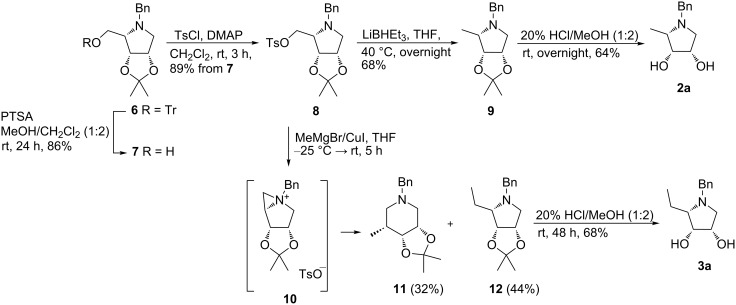
Synthesis of pyrrolidines **2a** and **3a**.

The synthesis of target compounds **2** and **3** started from imino-L-lyxitol **6** which was prepared in large quantity from D-ribose as described in our previous paper (Scheme 1) [30]. Conversion of trityl ether **6** into the tosyl derivative **8** was achieved by removal of the protecting trityl group under acidic conditions followed by tosylation of the liberated hydroxy group with TsCl in the presence of DMAP as a base. Thus, required tosyl derivative **8** was obtained in 76% yield in two steps. It should be emphasized that tosylation in the presence of commonly used bases such as pyridine or TEA was sluggish. Subsequent substitution of the tosylate in **8** either with Super-hydride^®^ (LiBHEt_3_) or with a cuprate generated in situ from MeMgBr and CuI afforded pyrrolidines **9** [31] and **12** in 68% and 44% yield, respectively (Scheme 1). In the course of tosylate substitution with the cuprate, 3-methylpiperidine **11** was isolated as a byproduct in 32% yield. A formation of the piperidine **11** proceeds via opening of aziridinium intermediate **10** (Scheme 1) [32]. Interestingly, a product of a ring expansion was not observed during the tosylate substitution with LiBHEt_3_. Simple acidic hydrolysis of acetonides **9** and **12** gave target compounds **2a** (64%) and **3a** (68%, Scheme 1).

**Scheme 2:**
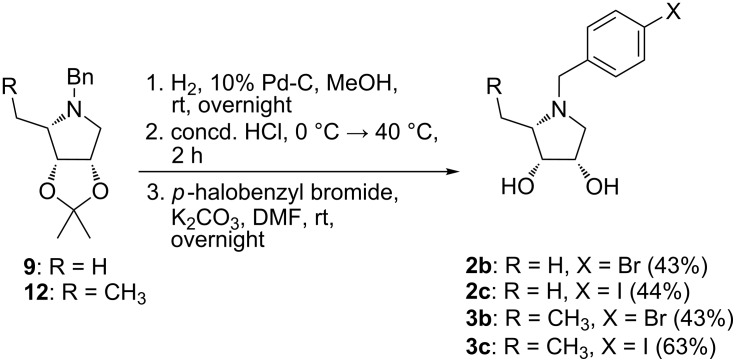
Synthesis of pyrrolidines **2b**,**c** and **3b**,**c**.

As amines prepared by catalytic hydrogenolysis of *N*-benzylpyrrolidines **9** and **12** were extremely volatile they were immediately subjected to acetonide hydrolysis without isolation. So-obtained hydrochlorides were used directly to the selective *N*-benzylation with the corresponding 4-halobenzyl bromide under basic conditions to provide compounds **2b**,**c** and **3b**,**c**. By this way, the final compounds **2b**,**c** and **3b**,**c** were accessed in three steps in good yields (43–63%, Scheme 2).

**Scheme 3:**
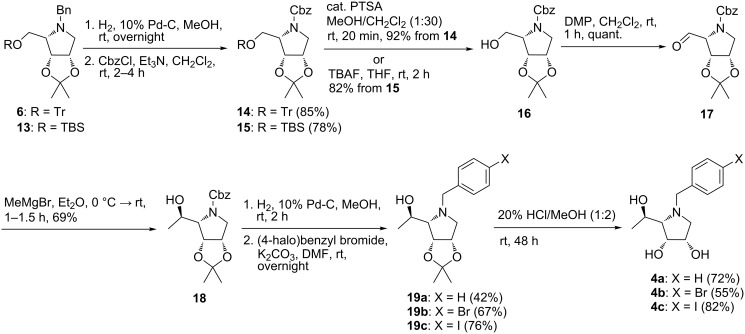
Synthesis of pyrrolidines **4**.

The next series of target pyrrolidines **4** and **5** could be achieved via nucleophilic addition of MeMgBr to an aldehyde obtained by the oxidation of alcohol **7**. Despite this fact, we did not manage the preparation of the aldehyde by the oxidation of alcohol **7** probably due to its instability [33]. However, similar aldehyde **17** bearing a Cbz protecting group instead of the benzyl moiety at the nitrogen atom was prepared as stable compound by Trajkovic et al. [34]. Therefore, our attention was focused on preparation of aldehyde **17** starting from protected pyrrolidines **6** and **13** (Scheme 3). The exchange of the benzyl group in **6** and **13** for a Cbz moiety was achieved by *N*-debenzylation under catalytic hydrogenolysis conditions followed by protection of the liberated amines with CbzCl furnishing fully protected pyrrolidines **14** [35] and **15**. Exposure of **14** to a catalytic amount of PTSA (0.04 equiv) in a mixture of CH_2_Cl_2_/MeOH 30:1 (v/v) resulted in rapid cleavage of the trityl ether providing known alcohol **16** [34]. Treatment of **15** with TBAF yielded identical alcohol **16** in good yield. As described by Trajkovic et al. [34], oxidation of alcohol **16** with DMP led cleanly to the desired aldehyde **17**. As some decomposition products were formed during flash chromatography on silica gel, the sensitive aldehyde **17** was used in the next step without further purification and characterization. Diastereoselective addition of MeMgBr to the aldehyde group of **17** gave alcohol **18** as single diastereoisomer in 69% yield. Removal of the Cbz protecting group of **18** under catalytic hydrogenolysis conditions furnished the free amine which was subsequently subjected to *N*-benzylation with the corresponding (4-halo)benzyl bromide to afford *N*-(4-halo)benzylpyrrolidines **19a**–**c**. Acidic hydrolysis of the acetonide protecting group in **19a**–**c** provided target compounds **4a**–**c** in good yields (Scheme 3).

**Scheme 4:**
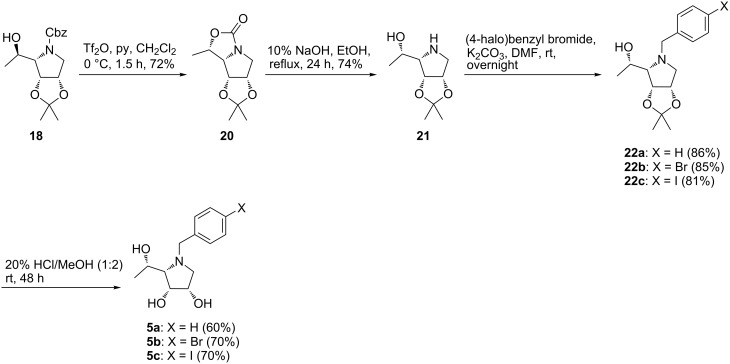
Synthesis of pyrrolidines **5**.

**Figure 2:**
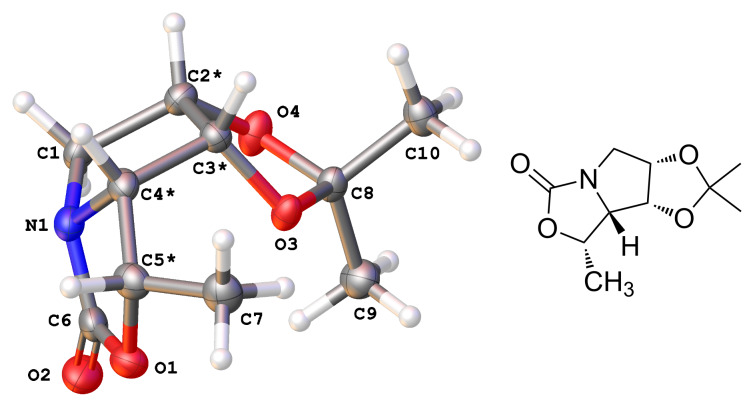
Molecular structure (OLEX2 drawing with adjacent ChemDraw image) of compound **20**. Atomic displacement ellipsoids are drawn at 50% probability level.

In order to obtain final compounds **5**, a configurational inversion of the stereocenter at C-5 in **18** was necessary. The inversion of the configuration was first attempted by a modified Mitsunobu reaction or activation of the hydroxy group by mesylation according to Trajkovic et al. [34]. However, these attempts resulted in either no reaction or formation of an unstable mesylate. For this reason, the inversion of configuration was performed by the activation of the hydroxy group in **18** with triflic anhydride in the presence of pyridine at 0 °C to form carbamate **20** in 72% yield (Scheme 4). The structure and absolute configuration of carbamate **20** was confirmed by single-crystal X-ray analysis (Figure 2) [36]. Basic hydrolysis of carbamate **20** with 10% NaOH in refluxing EtOH provided aminoalcohol **21** which was subsequently *N*-benzylated with the corresponding benzyl bromides to yield pyrrolidines **22**. Final hydrolysis of acetonides **22** in highly acidic media provided target compounds **5** (Scheme 4).

### Biochemical evaluation

*N*-(4-Halo)benzylpyrrolidines **2**–**5** were evaluated against the class II α-mannosidases GMIIb, LManII and JBMan from the GH38 family to investigate their ability to inhibit only selected enzyme. All pyrrolidines **2**–**5** demonstrated inhibitory activity against the target enzyme (GMIIb) with IC_50_ values in the range of 0.30 mM to 2.95 mM (Table 1). On the other hand, none of them was effective against LManII at 2 mM concentration. Therefore in this panel of tested enzymes the pyrrolidines **2–5** are selective inhibitors for GMIIb.

**Table 1:** Inhibitory activity of pyrrolidines **2–5** against the class II α-mannosidases GMIIb, LManII and JBMan from GH38 family enzymes.

Compound	GMIIb [IC_50_ (M)]	LmanII [IC_50_ (M)]	JBMan^a^ (%)

**1a** [30]	(8.8 ± 0.06) × 10^−5^	7.0 × 10^−3^	1
**2a**	(3.6 ± 0.20) × 10^−4^	n.i.^a^	n.i.
**2b**	(3.5 ± 0.17) × 10^−4^	n.i.^a^	n.i.
**2c**	(3.0 ± 0.18) × 10^−4^	n.i.^a^	n.i.
**3a**	(7.5 ± 0.35) × 10^−4^	n.i.^a^	3
**3b**	(9.5 ± 0.33) × 10^−4^	n.i.^a^	14
**3c**	(4.5 ± 0.14) × 10^−4^	n.i.^a^	39
**4a**	(18.0 ± 0.40) × 10^−4^	n.i.^a^	2
**4b**	(16.0 ± 0.40) × 10^−4^	n.i.^a^	4
**4c**	(19.0 ± 0.46) × 10^−4^	n.i.^a^	17
**5a**	(19.5 ± 0.41) × 10^−4^	n.i.^a^	21
**5b**	(26.5 ± 0.51) × 10^−4^	n.i.^a^	40
**5c**	(29.5± 0.56) × 10^−4^	n.i.^a^	38

^a^Inhibition in the presence of 2 mM inhibitor concentration; n.i. no inhibition.

The inhibitory activity of the tested pyrrolidines against GMIIb was affected by modification at C-5 of the imino-L-lyxitol core. Elongation of the C-5 position in pyrrolidine **1a** by a methyl group led to a pair of diastereoisomers **4a** and **5a** which differ in the configuration at the new C-5 stereocenter. This elongation led to about 25-fold decrease in inhibition activity against GMIIb in comparison with **1a**. It is interesting that the increase of IC_50_ values was essentially not influenced by the stereochemistry at C-5 in **4a** and **5a**. On the contrary, the introduction of a halogen atom to the para position of the benzyl substituent had a certain effect. While the inhibitory activity of **4b** and **4c** was very similar to **4a**, the halogenated counterparts of **5a**, **5b** and **5c** showed slightly reduced inhibitions of GMIIb. Some differences were also observed in their effects on JBMan. Pyrrolidine **4c** showed moderate inhibitory activity comparable with **5a**, while **5b**,**c** were the most active, but still poor (40% inhibition at 2 mM concentration) inhibitors of this enzyme among all tested pyrrolidines. Structural modifications in pyrrolidines **4a** and **4b** showed negligible influence on the activity against JBMan.

Pyrrolidines **3** represent deoxygenated analogs of **4** and **5**, and their efficiencies against JBMan were similar. In comparison with the latter compounds, inhibition capacity of **3** against GMIIb was improved, each showing an IC_50_ value lower than 1 mM, in case of the most efficient **3c** even below 0.5 mM.

Further improvement of the GMIIb inhibition was achieved by the removal of the primary hydroxy group from C-5 position in **1** leading to deoxygenated analogs **2**. In series of compound **2** only a weak impact of the halogen substituent at the aromatic unit on the efficiency of the inhibition was observed. Pyrrolidine **2a**, as well as its *N*-(4-halobenzyl) derivatives **2b** and **2c**, exhibited similar IC_50_ values in the range of 0.30–0.36 mM. The most potent was *N*-(4-iodobenzyl)pyrrolidine **2c** (IC_50_ 0.30 mM). In regard to the inhibition pattern against GMIIb, the results are in agreement with our previous study for (4-halo)benzylpyrrolidines **1** with retained primary hydroxymethyl function [30]. In both series of pyrrolidines **1** and **2**, the presence of a halogen substituent at the aromatic unit slightly improved efficiency of the GMIIb inhibition. However, the deoxygenation of the hydroxymethyl function to a methyl group (compounds **2**) led to approximately 4-fold decrease in potency (benzylpyrrolidine **1a** was used as a reference compound in this assay, IC_50_ 0.088 mM). None of the pyrrolidines **2** affected JBMan at 2mM concentration.

In summary, taking into account no significant influence on other tested GH38 mannosidases (LManII and JBMan), all synthesized derivatives **2**–**5** having C-5 modified pyrrolidine core were identified as selective inhibitors of the target GMIIb enzyme. Nature and size of the functional group at position 5 of the pyrrolidine core has a limited impact on the activity against the GMIIb which was decreasing with increasing size of this functional group, suggesting a certain role of steric effect. An even smaller effect was found for 4-halogenation of the *N*-benzyl substituent, arguing against a steric or electron effect at this position of the phenyl ring. Nevertheless, the synthesis of different carbohydrate based scaffolds and their evaluation against a given target is of importance with aim to reveal the role of the saccharide core and aromatic moiety for the interaction with the target enzyme.

## Conclusion

A synthetic approach to imino-L-lyxitols with modification at C-5 is described. These new carbohydrate analogues may be utilized as structural motifs for a development of selective inhibitors of GMIIb as a target GH38 enzyme. The synthesized modified pyrrolidines represent a new scaffold with a promising potential to be used in the design of new bioactive compounds. However, further improvement of their potency against the target enzyme is needed. This could be achieved by introducing of different interacting group(s) at the aromatic unit that would ensure stronger interaction with the target enzyme.

## Supporting Information

File 1 Experimental procedures and analytical data.

File 2 Copies of ^1^H and ^13^C NMR spectra of all prepared compounds.
