# Supplementary material for: Synthesis of 1,4-imino-L-lyxitols modified at C-5 and their evaluation as inhibitors of GH38 α-mannosidases
Source: Beilstein J Org Chem. 2018 Aug 17;14:2156–62. doi: 10.3762/bjoc.14.189 (PMC6122390; doi:10.3762/bjoc.14.189)
Supplement: File 1 — Experimental procedures and analytical data. [file Beilstein_J_Org_Chem-14-2156-s001.pdf]

Supporting Information

for

Synthesis of 1,4-imino-L-lyxitols modified at C-5 and their evaluation as inhibitors of GH38 α-mannosidases

Maroš Bella[1], Sergej Šesták[1], Ján Moncoľ[2], Miroslav Koóš[1] and Monika Poláková*[1]

Address: [1]Department of Glycochemistry, Institute of Chemistry, Slovak Academy of Sciences Dúbravská cesta 9, SK-845 38, Bratislava, Slovakia and [2]Department of Inorganic Chemistry, Faculty of Chemical and Food Technology, Radlinského 9, SK-812 37 Bratislava, Slovakia

Email: Monika Polákova - chemonca@savba.sk

*Corresponding author

Experimental procedures and analytical data

Experimental Section
All commercially available reagents and anhydrous solvents were used as received. Solvents of technical grade were distilled before use. Dichloromethane was boiled under reflux over P_2O_5 for 1 h and was distilled immediately prior to use. Melting points were determined using a Boetius PHMK 05 microscope. Specific optical rotations were determined on a Jasco P-2000 polarimeter. ^{1}H NMR and ^{13}C NMR spectra were recorded at 25 °C with a Bruker AVANCE III HD 400 spectrometer. Chemical shifts are given in ppm (δ) relative to residual signal of appropriate deuterated solvent used ($CDCl_3$, CD_3OD). Coupling constants are given in Hz. All given ^{13}C spectra are proton decoupled. The 2D NMR experiments (HSQC and COSY) were used for the signal assignments. High-resolution mass spectra were obtained using Orbitrap Elite (Thermo Scientific) mass spectrometer with ESI ionization in positive mode. The IR spectra (ATR) were measured using a Nicolet 6700 FTIR spectrometer. Thin-layer chromatography (TLC) was performed on glass plates pre-coated with TLC Silica gel 60 F_{254} (E. Merck). Visualization was achieved by immersing the plates into PMA solution (10% solution of phosphomolybdic acid in ethanol) or $KMnO_4$ solution (3 g of $KMnO_4$, 20 g, K_2CO_3, 2.5 mL of 10% NaOH and 400 mL of water) and heating at ca 200 °C with a heat gun. Column chromatography was performed as flash chromatography on Silica gel 60 (E. Merck, 0.040–0.063 mm). Solvents used for flash chromatography were of technical grade and distilled before use. Data collection and cell refinement of **20** were made on a Stoe StadiVari diffractometer using Pilatus3R 300K detector and microfocused X-ray source Xenocs Genix 3D Cu HF at 100K. The structure was solved by the charge-flipping method using SUPERFLIP program and refined by the full-matrix least-squares procedure with SHELXL (version 2018/1).[1,2] The structure was drawn using OLEX2 package.[3] The absolute structure and configuration was determined. The Flack parameter was calculated by the Parsons method.[4]

((3a*R*,4*S*,6a*S*)-5-Benzyl-2,2-dimethyltetrahydro-3a*H*-[1,3]dioxolo[4,5-*c*]pyrrol-4-yl)methanol (7). To a stirred solution of trityl ether **6** (4.59 g, 9.07 mmol) in MeOH (45 mL) and CH_2Cl_2 (90 mL), *p*-toluenesulfonic acid monohydrate (2.59 g, 13.61 mmol) was added and the stirring was continued for 24 h. Next, *p*-toluenesulfonic acid was neutralized with concd. aqueous NH_3 (3 mL), the resulting mixture was treated with silica gel (25 g) and volatiles were evaporated. The residue was purified by column chromatography (EtOAc/hexane 2:3) to afford alcohol **7** as colorless oil (2.06 g, 86%). R_f = 0.17 (EtOAc/hexane 2:3); $[\alpha]_D^{20}$ = +81.7 (*c* = 0.53, $CHCl_3$) (Ref.[5] $[\alpha]_D^{25}$ = +101.8 (*c* = 1.9, MeOH)); IR (ATR) *v* = 3436, 2935, 1371, 1206, 1093, 699 cm^{-1}; ^{1}H NMR (400 MHz, $CDCl_3$) δ = 7.37–7.17 (m, 5H, Ph), 4.71 (dd, *J* = 6.4, 4.8 Hz, 1H, H-3), 4.59 (dd, *J* = 6.4, 4.6 Hz, 1H, H-2), 4.05 (d, *J* = 13.4 Hz, 1H, NCH_2Ph), 3.99–3.88 (m, 2H, H-5), 3.22 (d, *J* = 13.4 Hz, 1H, NCH_2Ph), 3.08 (d, *J* = 11.0 Hz, 1H, H-1a), 2.62 (br s, 1H, OH), 2.37 (q, *J* = 4.8 Hz, 1H, H-4), 2.13 (dd, *J* = 11.0, 4.6 Hz, 1H, H-1b), 1.55 and 1.32 [2s, each 3H, C(CH_3)$_2$] ppm; ^{13}C NMR (100 MHz, $CDCl_3$) δ = 138.0 (Ph), 128.6 (Ph), 128.2 (Ph), 126.9 (Ph), 111.4 [*C*(CH_3)], 81.8 (C-3), 77.8 (C-2), 67.1 (C-4), 59.7 (C-5), 58.7 (C-1), 56.7 (NCH_2Ph), 26.1 and 25.0 [C(CH_3)$_2$] ppm; HRMS (ESI, m/z): calculated for $C_{15}H_{21}NO_3$ $[M+H]^+$: 264.1594; found: 264.1593.

((3a*R*,4*S*,6a*S*)-5-Benzyl-2,2-dimethyltetrahydro-3a*H*-[1,3]dioxolo[4,5-*c*]pyrrol-4-yl)methyl 4-methylbenzenesulfonate (8). To a stirred solution of alcohol **7** (0.77 g, 2.92 mmol) in CH_2Cl_2 (15 mL), DMAP (0.71 g, 5.81 mmol) was added. The resulting solution was cooled in an ice-water bath to 0 °C and *p*-toluenesulfonyl chloride (0.78 g, 4.09 mmol) was added in one portion. After 15 min of stirring, the ice-water bath was removed and the stirring was continued at rt for 3 h. The reaction mixture was washed with water (2×15 mL), separated organic layer was dried (Na_2SO_4), filtered and the solvent was evaporated. The crude product was purified by column chromatography (EtOAc/hexane 1:4) to afford tosylate **8** as white solid (1.09 g, 89%). M.p. 65-67 °C; R_f = 0.21 (EtOAc/hexane 1:4); $[\alpha]_D^{20}$ = +89.9 (*c* = 0.70, $CHCl_3$); IR (ATR) *v* = 1369, 1176, 1105, 977, 665 cm^{-1}; ^{1}H NMR (400 MHz, $CDCl_3$) δ = 7.81 (d, *J* = 8.4 Hz, 2H, OTs), 7.34 (d, *J* = 8.4 Hz, 2H, OTs), 7.31–7.18 (m, 5H, Ph), 4.63 (dd, *J* = 6.4, 4.8 Hz, 1H, H-3), 4.58 (dd, *J* = 6.4, 4.4 Hz, 1H, H-2), 4.34 (dd, *J* = 10.1, 6.5 Hz, 1H, H-5a), 4.19 (dd, *J* = 10.1, 5.2 Hz, 1H, H-5b), 3.98 (d, *J* = 13.7 Hz, 1H, NCH_2Ph), 3.28 (d, *J* = 13.7 Hz, 1H, NCH_2Ph), 3.04 (d, *J* = 11.3 Hz, 1H, H-1a), 2.63 (q, *J* = 5.5 Hz, 1H, H-4), 2.47 (s, 3H, CH_3-$C_6H_4SO_2$), 2.12 (dd, *J* = 11.3, 4.4 Hz, 1H, H-1b), 1.40 and 1.26 [2s, each 3H, C(CH_3)$_2$] ppm; ^{13}C NMR (100 MHz, $CDCl_3$) δ = 144.6 (OTs), 137.9 (Ph), 132.8 (OTs), 129.7 (Ph), 128.4 (Ph), 128.2 (Ph), 128.1 (OTs), 127.0 (Ph), 111.5 [*C*(CH_3)], 80.2 (C-3), 77.9 (C-2), 68.8 (C-5), 65.8 (C-4), 59.3 (C-1), 57.6 (NCH_2Ph), 26.1 and 25.3 [C(CH_3)$_2$], 21.6 (CH_3-$C_6H_4SO_2$) ppm; HRMS (ESI, m/z): calculated for $C_{22}H_{27}NO_5S$ $[M+H]^+$: 418.1683; found: 418.1684.

(3a*R*,4*S*,6a*S*)-5-Benzyl-2,2,4-trimethyltetrahydro-3a*H*-[1,3]dioxolo[4,5-*c*]pyrrole (9). To a stirred solution of tosylate **8** (0.56 g, 1.34 mmol) in anhydrous THF (15 mL), lithium triethylborohydride (1.7 M in THF, 4.8 mL, 8.16 mmol) was added dropwise while cooling in an ice-water bath to 0 °C. After 10 min of stirring, the ice-water bath was removed and the reaction mixture was heated at 40 °C overnight. Next, the reaction mixture was cooled in the ice-water bath and was carefully quenched with water (1 mL). The volatiles were evaporated and the residue was partitioned between EtOAc (20 mL) and water (20 mL). Aqueous layer was extracted with additional EtOAc (20 mL), combined extracts were dried (Na_2SO_4), filtered and the solvent was evaporated. The crude product was purified by column chromatography (EtOAc/hexane 1:6) to afford imino-L-lyxitol **9** as thin colorless oil (0.23 g, 68%). R_f = 0.26 (EtOAc/hexane 1:6); $[\alpha]_D^{20}$ = +37.2 (*c* = 0.57, $CHCl_3$) (Ref.[6] $[\alpha]_D^{20}$ = +33.4 (*c* = 0.70, $CHCl_3$)); IR (ATR) *v* = 2932, 2782, 1377, 1206, 1117, 698 cm^{-1}; ^{1}H NMR (400 MHz, $CDCl_3$) δ = 7.35–7.20 (m, 5H, Ph), 4.58 (dd, *J* = 6.4, 4.6 Hz, 1H, H-2), 4.51 (dd, *J* = 6.4, 4.6 Hz, 1H, H-3), 4.04 (d, *J* = 13.5 Hz, 1H, NC*H*$_2$Ph), 3.11 (d, *J* = 13.5 Hz, 1H, NC*H*$_2$Ph), 3.03 (d, *J* = 11.1 Hz, 1H, H-1a), 2.27–2.17 (m, 1H, H-4), 1.98 (dd, *J* = 11.1, 4.6 Hz, 1H, H-1b), 1.57 and 1.35 [2s, each 3H, C(C*H*$_3$)$_2$], 1.25 (d, *J* = 6.3 Hz, 3H, CH_3) ppm; ^{13}C NMR (100 MHz, $CDCl_3$) δ = 138.7 (Ph), 128.5 (Ph), 128.1 (Ph), 126.7 (Ph), 111.0 [*C*(CH$_3$)$_2$], 82.5 (C-3), 78.0 (C-2), 62.8 (C-4), 59.4 (C-1), 56.6 (N*C*H$_2$Ph), 26.3 and 25.7 [C(*C*H$_3$)$_2$], 12.5 (C-5) ppm; HRMS (ESI, m/z): calculated for $C_{15}H_{21}NO_2$ [*M*+H]$^+$: 248.1645; found: 248.1644.

(2*S*,3*R*,4*S*)-1-Benzyl-2-methylpyrrolidine-3,4-diol (2a). To a stirred solution of 2,3-*O*-isopropylidene-L-lyxitol **9** (84 mg, 0.34 mmol) in MeOH (3 mL), 20% HCl (1.5 mL) was added while cooling in an ice-water bath. After 15 min of stirring, the ice-water bath was removed and the stirring was continued overnight at rt. Then, HCl was carefully neutralized with solid Na_2CO_3 (0.6 g). The resulting suspension was filtered and the filtrate was evaporated to dryness. The residue was suspended in MeOH (10 mL), filtered and the filtrate was concentrated. The residue was purified by column chromatography (MeOH/$CHCl_3$ 1:10 containing 0.5% (v/v) of concd. aqueous NH_3) to afford imino-L-lyxitol **2a** as thick colorless oil (45 mg, 64%). R_f = 0.32 (MeOH/$CHCl_3$ 1:10 containing 0.5% (v/v) of concd. aqueous NH_3); $[\alpha]_D^{20}$ = +91.1 (*c* = 0.58, MeOH); IR (ATR) *v* = 3362, 2931, 1452, 1122, 698 cm^{-1}; ^{1}H NMR (400 MHz, CD_3OD) δ = 7.38–7.19 (m, 5H, Ph), 4.13 (ddd, *J* = 6.8, 5.8, 3.3 Hz, 1H, H-2), 3.96 (t, *J* = 5.8 Hz, 1H, H-3), 3.94 (d, *J* = 12.9 Hz, 1H, NC*H*$_2$Ph), 3.24 (d, *J* = 12.9 Hz, 1H, NC*H*$_2$Ph), 2.77 (dd, *J* = 10.8, 3.3 Hz, 1H, H-1a), 2.61–2.52 (m, 1H, H-4), 2.46 (dd, *J* = 10.8, 6.8 Hz, 1H, H-1b), 1.18 (d, *J* = 6.5 Hz, 3H, CH_3) ppm; ^{13}C NMR (100 MHz, CD_3OD) δ = 139.2 (Ph), 130.4 (Ph), 129.2 (Ph), 128.1 (Ph), 74.3 (C-3), 70.4 (C-2), 63.9 (C-4), 60.4 (C-1), 59.0 (N*C*H$_2$Ph), 13.1 (C-5) ppm; HRMS (ESI, m/z): calculated for $C_{12}H_{17}NO_2$ [*M*+H]$^+$: 208.1332; found: 208.1331.

General procedure for the preparation of compound 2b and 2c

A suspension of imino-L-lyxitol (**9**) (200 mg, 0.80 mmol) and 10% palladium on carbon (20 mg, 10% by weight) in MeOH (8 mL) was stirred at rt in a hydrogen atmosphere (balloon) overnight. The catalyst was filtered off and the filtrate was treated with concd. HCl (0.3 mL) at 0°C. Next, the mixture was stirred at 40 °C for 2 h and the solvents were evaporated to dryness to give the corresponding hydrochloride as white solid (120 mg, 96%) which was used in next reactions without further purification and characterization. To a suspension of the hydrochloride (40 mg, 0.26 mmol) in anhydrous DMF (1.5 mL), K_2CO_3 (108 mg, 0.78 mmol) was added followed by corresponding benzyl bromide (1.2 equiv). After stirring at rt overnight, the mixture was partitioned between ethyl acetate (10 mL) and water (10 mL). Organic layer was washed with water (1 × 10 mL), dried (Na_2SO_4), filtered and the solvent was evaporated. The residue was purified by column chromatography (MeOH/$CHCl_3$ 1:20 containing 0.5% (v/v) of concd. aqueous NH_3).

(2*S*,3*R*,4*S*)-1-(4-Bromobenzyl)-2-methylpyrrolidine-3,4-diol (2b). The reaction was carried out according to general procedure with 4-bromobenzyl bromide (78 mg, 0.31 mmol) to afford pyrrolidine **2b** as thick colorless oil (32 mg, 43%). R_f = 0.25 (MeOH/$CHCl_3$ 1:20 containing 0.5% (v/v) of concd. aqueous NH_3); $[\alpha]_D^{20}$ = +42.3 (*c* = 0.42, MeOH); IR (ATR) *v* = 3334, 2931, 1487, 1124, 1070, 1011 cm^{-1}; ^{1}H NMR (400 MHz, CD_3OD) δ = 7.45 (d, *J* = 6.5 Hz, 2H, Ar), 7.27 (d, *J* = 6.5 Hz, 2H, Ar), 4.15 (ddd, *J* = 6.8, 5.8, 3.4 Hz, 1H, H-2), 3.97 (t, *J* = 5.5 Hz, 1H, H-3), 3.91 (d, *J* = 13.2 Hz, 1H, NC*H*$_2$Ar), 3.22 (d, *J* = 13.2 Hz, 1H, NC*H*$_2$Ar), 2.77 (dd, *J* = 10.7, 3.4 Hz, 1H, H-1a), 2.57 (p, *J* = 6.5 Hz, 1H, H-4), 2.44 (dd, *J* = 10.7, 6.8 Hz, 1H, H-1b), 1.17 (d, *J* = 6.5 Hz, 3H, C*H*$_3$) ppm; ^{13}C NMR (100 MHz, CD_3OD) δ = 138.9 (Ar), 132.3 (Ar), 132.2 (Ar), 121.8 (Ar), 74.3 (C-3), 70.5 (C-2), 63.9 (C-4), 60.4 (C-1), 58.3 (N*C*H$_2$Ar), 13.2 (C-5) ppm; HRMS (ESI, m/z): calculated for $C_{12}H_{16}BrNO_2$ [*M*+H]$^+$: 286.0437; found: 286.0435.

(2*S*,3*R*,4*S*)-1-(4-Iodobenzyl)-2-methylpyrrolidine-3,4-diol (2c). The reaction was carried out according to general procedure with 4-iodobenzyl bromide (93 mg, 0.31 mmol) to afford pyrrolidine **2c** as thick colorless oil (38 mg, 44%). R_f = 0.16 ($MeOH/CHCl_3$ 1:20 containing 0.5% (v/v) of concd. aqueous NH_3); $[\alpha]_D^{20}$ = +26.6 (*c* = 0.65, MeOH); IR (ATR) *v* = 3349, 2929, 1482, 1122, 1005, 795 cm^{-1}; ^1H NMR (400 MHz, CD_3OD) δ = 7.65 (d, *J* = 8.3 Hz, 2H, Ar), 7.14 (d, *J* = 8.3 Hz, 2H, Ar), 4.14 (ddd, *J* = 6.8, 5.8, 3.4 Hz, 1H, H-2), 3.96 (t, *J* = 5.5 Hz, 1H, H-3), 3.90 (d, *J* = 13.2 Hz, 1H, NC*H*$_2$Ar), 3.21 (d, *J* = 13.2 Hz, 1H, NC*H*$_2$Ar), 2.77 (dd, *J* = 10.7, 3.4 Hz, 1H, H-1a), 2.57 (qd, *J* = 6.4, 5.0 Hz, 1H, H-4), 2.44 (dd, *J* = 10.7, 6.8 Hz, 1H, H-1b), 1.17 (d, *J* = 6.4 Hz, 3H, C*H*$_3$) ppm; ^{13}C NMR (100 MHz, CD_3OD) δ = 139.5 (Ar), 138.4 (Ar), 132.4 (Ar), 93.0 (Ar), 74.3 (C-3), 70.5 (C-2), 63.9 (C-4), 60.3 (C-1), 58.4 (N*C*H$_2$Ar), 13.2 (C-5) ppm; HRMS (ESI, m/z): calculated for $C_{12}H_{16}INO_2$ $[M+H]^+$: 334.0298; found: 334.0294.

(3a*S*,7*R*,7a*R*)-5-Benzyl-2,2,7-trimethylhexahydro-[1,3]dioxolo[4,5-*c*]pyridine (11) and (3a*R*,4*S*,6a*S*)-5-benzyl-4-ethyl-2,2-dimethyltetrahydro-3a*H*-[1,3]dioxolo[4,5-*c*]pyrrole (12). To a solution of CuI (1.38 g, 7.25 mmol) in anhydrous THF (20 mL), MeMgBr (3M in ether, 6 mL, 18.1 mmol) was added dropwise at −25 °C and the mixture was stirred for 30 min. Then, a solution of tosylate **8** (1.51 g, 3.6 mmol) in anhydrous THF (15 mL) was added dropwise. After 5 min of stirring, the mixture was slowly allowed to warm up to rt and stirring was continued for 5 h. Once the reaction was complete, the mixture was cooled in an ice-water bath and was carefully quenched with saturated solution of NH_4Cl (5 mL). The resulting mixture was partitioned between diethyl ether (100 mL) and water (100 mL). Aqueous layer was extracted with additional diethyl ether (50 mL), combined extracts were dried (Na_2SO_4), filtered and the solvent was evaporated. Particular products were separated by column chromatography (EtOAc/hexane 1:5).

(3a*S*,7*R*,7a*R*)-5-Benzyl-2,2,7-trimethylhexahydro-[1,3]dioxolo[4,5-*c*]pyridine (11): Colorless oil (0.30 g, 32%); R_f = 0.13 (EtOAc/hexane 1:5); $[\alpha]_D^{20}$ = −43.4 (*c* = 0.61, $CHCl_3$); IR (ATR) *v* = 2989, 1241, 1217, 1043, 1019, 751 cm^{-1}; ^1H NMR (400 MHz, $CDCl_3$) δ = 7.34–7.21 (m, 5H, Ph), 4.15 (ddd, *J* = 9.7, 6.7, 4.7 Hz, 1H, H-3*a*), 4.08 (t, *J* = 4.2 Hz, 1H, H-7*a*), 3.50 (d, *J* = 13.2 Hz, 1H, NC*H*$_2$Ph), 3.47 (d, *J* = 13.2 Hz, 1H, NC*H*$_2$Ph), 2.87 (ddd, *J* = 11.1, 6.7, 2.0 Hz, 1H, H-4a), 2.51 (ddd, *J* = 11.0, 4.8, 2.0 Hz, 1H, H-6a), 2.12–2.01 (m, 1H, H-7), 1.97 (dd, *J* = 11.1, 9.7 Hz, 1H, H-4b), 1.88 (t, *J* = 11.0 Hz, 1H), 1.48 and 1.34 [2s, each 3H, C(C*H*$_3$)$_2$], 1.02 (d, *J* = 7.0 Hz, 3H, C*H*$_3$) ppm; ^{13}C NMR (100 MHz, $CDCl_3$) δ = 138.2 (Ph), 128.9 (Ph), 128.2 (Ph), 127.0 (Ph), 108.2 [*C*(CH$_3$)$_2$], 75.7 (C-7a), 73.0 (C-3a), 62.6 (N*C*H$_2$Ph), 55.7 (C-4), 55.2 (C-6), 31.9 (C-7), 28.4 and 26.4 [C(*C*H$_3$)$_2$], 14.8 (*C*H$_3$) ppm; HRMS (ESI, m/z): calculated for $C_{16}H_{23}NO_2$ $[M+H]^+$: 262.1802; found: 262.1799.

(3a*R*,4*S*,6a*S*)-5-Benzyl-4-ethyl-2,2-dimethyltetrahydro-3a*H*-[1,3]dioxolo[4,5-*c*]pyrrole (12): thin colorless oil (0.42 g, 44%); R_f = 0.33 (EtOAc/hexane 1:5); $[\alpha]_D^{20}$ = +117.9 (*c* = 0.62, $CHCl_3$); IR (ATR) *v* = 2963, 2782, 1119, 1025, 736 cm^{-1}; ^1H NMR (400 MHz, $CDCl_3$) δ = 7.34–7.19 (m, 5H, Ph), 4.62 (dd, *J* = 6.5, 4.5 Hz, 1H, H-3*a*), 4.56 (dd, *J* = 6.5, 4.6 Hz, 1H, H-6a), 4.07 (d, *J* = 13.6 Hz, 1H, NC*H*$_2$Ph), 3.09 (d, *J* = 13.6 Hz, 1H, NC*H*$_2$Ph), 3.03 (d, *J* = 11.1 Hz, 1H, H-6*a*), 2.04–1.98 (m, 1H, H-4), 1.96 (dd, *J* = 11.1, 4.6 Hz, 1H, H-6*b*), 1.78–1.68 (m, 2H, C*H*$_2$CH$_3$), 1.56 and 1.34 [2s, each 3H, C(C*H*$_3$)$_2$], 1.04 (t, *J* = 7.4 Hz, 3H, CH$_2$C*H*$_3$) ppm; ^{13}C NMR (100 MHz, $CDCl_3$) δ = 138.8 (Ph), 128.5 (Ph), 128.1 (Ph), 126.7 (Ph), 110.9 [*C*(CH$_3$)$_2$], 80.7 (C-3a), 77.7 (C-6a), 69.8 (C-4), 59.7 (C-6), 56.8 (N*C*H$_2$Ph), 26.3 and 25.8 [C(*C*H$_3$)$_2$], 20.1 (*C*H$_2$CH$_3$), 10.7 (CH$_2$*C*H$_3$) ppm; HRMS (ESI, m/z): calculated for $C_{16}H_{23}NO_2$ $[M+H]^+$: 262.1802; found: 262.1800.

(2*S*,3*R*,4*S*)-1-Benzyl-2-ethylpyrrolidine-3,4-diol (3a). To a stirred solution of acetonide **12** (47 mg, 0.18 mmol) in MeOH (1.2 mL), 20% HCl (0.6 mL) was added while cooling in an ice-water bath. After 15 min of stirring, the ice-water bath was removed and the stirring was continued for 48 h at rt. Then, HCl was carefully neutralized with solid Na_2CO_3 (0.28 g). The resulting suspension was filtered and the filtrate was concentrated. The residue was purified by column chromatography ($MeOH/CHCl_3$ 1:20 containing 0.5% (v/v) of concd. aqueous NH_3) to afford diol **3a** as white solid (27 mg, 68%). M.p. 76–78 °C; R_f = 0.19 ($MeOH/CHCl_3$ 1:20 containing 0.5% (v/v) of concd. aqueous NH_3); $[\alpha]_D^{20}$ = +112.9 (*c* = 0.53, MeOH); IR (ATR) *v* = 3467, 2967, 1352, 1112, 1019, 739 cm^{-1}; ^1H NMR (400 MHz, CD_3OD) 7.35–7.20 (m, 5H, Ph), 4.12 (ddd, *J* = 7.2, 5.5, 4.3 Hz, 1H, H-4), 4.06 (dd, *J* = 5.5, 4.5 Hz, 1H, H-3), 3.97 (d, *J* = 13.0 Hz, 1H, NC*H*$_2$Ph), 3.24 (d, *J* = 13.0 Hz, 1H, NC*H*$_2$Ph), 2.78 (dd, *J* = 10.8, 4.3 Hz, 1H, H-5a), 2.50 (dd, *J* = 10.8, 7.2 Hz, 1H, H-5b), 2.39–2.32 (m, 1H, H-2), 1.81–1.60 (m, 2H, C*H*$_2$CH$_3$), 0.99 (t, *J* = 7.4 Hz, 3H, CH$_2$C*H*$_3$) ppm; ^{13}C NMR (100 MHz, CD_3OD) δ = 139.5 (Ph), 130.4 (Ph), 129.2 (Ph), 128.1 (Ph), 72.4 (C-3), 70.8 (C-2), 70.5 (C-4), 60.1 (C-5), 59.5 (N*C*H$_2$Ph), 21.4 (*C*H$_2$CH$_3$), 11.2 (CH$_2$*C*H$_3$) ppm; HRMS (ESI, m/z): calculated for $C_{13}H_{19}NO_2$ $[M+H]^+$: 222.1489; found: 222.1486.

General procedure for the preparation of compound 3b and 3c

A suspension of *N*-benzylpyrrolidine (**12**) (200 mg, 0.86 mmol) and 10% palladium on carbon (20 mg, 10% by weight) in MeOH (8 mL) was stirred at rt in a hydrogen atmosphere (balloon) overnight. The catalyst was filtered off and the filtrate was treated with concd. HCl (0.3 mL) at 0 °C. Next, the mixture was stirred at 40 °C for 2 h and the solvents were evaporated to give corresponding hydrochloride as white solid (122 mg, 95%) which was used in next reactions without further purification and characterization. To a suspension of the hydrochloride (60 mg, 0.36 mmol) in anhydrous DMF (2 mL), K_2CO_3 (148 mg, 1.07 mmol) was added followed by the addition of corresponding benzyl bromide (1.2 equiv). After stirring at rt overnight, the mixture was partitioned between ethyl acetate (10 mL) and water (10 mL). Organic layer was washed with water (1 × 10 mL), dried (Na_2SO_4), filtered and the solvent was evaporated. The residue was purified by column chromatography (MeOH/$CHCl_3$ 1:20 containing 0.5% (v/v) of concd. aqueous NH_3).

(2*S*,3*R*,4*S*)-2-Ethyl-1-(4-bromobenzyl)pyrrolidine-3,4-diol (3b). The reaction was carried out according to general procedure with 4-bromobenzyl bromide (107 mg, 0.43 mmol) to afford pyrrolidine **3b** as thick yellowish oil (47 mg, 43%). R_f = 0.24 (MeOH/$CHCl_3$ 1:20 containing 0.5% (v/v) of concd. aqueous NH_3); $[\alpha]_D^{20}$ = +65.6 (*c* = 0.62, MeOH); IR (ATR) *v* = 3345, 2963, 1468, 1123, 1011, 802 cm^{-1}; ^1H NMR (400 MHz, $CDCl_3$) δ = 7.43 (d, *J* = 8.3 Hz, 2H, Ar), 7.16 (d, *J* = 8.3 Hz, 2H, Ar), 4.18–4.06 (m, 2H, H-3, H-4), 3.96 (d, *J* = 13.2 Hz, 1H, NCH_2Ar), 3.10 (d, *J* = 13.2 Hz, 1H, NCH_2Ar), 2.77 (dd, *J* = 10.8, 2.5 Hz, 1H, H-5a), 2.56 (br s, 2H, 2 × O*H*), 2.37–2.26 (m, 2H, H-2, H-5b), 1.77–1.55 (m, 2H, CH_2CH$_3$), 1.03 (t, *J* = 7.4 Hz, 3H, CH$_2$CH_3) ppm; ^{13}C NMR (100 MHz, $CDCl_3$) δ = 137.4 (Ar), 131.4 (Ar), 130.4 (Ar), 120.8 (Ar), 71.9 (C-3), 69.8 (C-4), 68.7 (C-2), 59.8 (C-5), 56.8 (NCH_2Ar), 20.6 (CH_2CH$_3$), 10.7 (CH$_2$$CH_3$) ppm; HRMS (ESI, m/z): calculated for $C_{13}H_{18}BrNO_2$ $[M+H]^+$: 300.0594; found: 300.0589.

(2*S*,3*R*,4*S*)-2-Ethyl-1-(4-iodobenzyl)pyrrolidine-3,4-diol (3c). The reaction was carried out according to general procedure with 4-iodobenzyl bromide (127 mg, 0.43 mmol) to afford pyrrolidine **3c** as thick yellowish oil (85 mg, 63%). R_f = 0.27 (MeOH/$CHCl_3$ 1:20 containing 0.5% (v/v) of concd. aqueous NH_3); $[\alpha]_D^{20}$ = +68.3 (*c* = 0.51, MeOH); IR (ATR) *v* = 3354, 2961, 1483, 1122, 1006, 797 cm^{-1}; ^1H NMR (400 MHz, $CDCl_3$) δ = 7.63 (d, *J* = 8.2 Hz, 2H, Ar), 7.03 (d, *J* = 8.2 Hz, 2H, Ar), 4.16–4.07 (m, 2H, H-3, H-4), 3.95 (d, *J* = 13.3 Hz, 1H, NCH_2Ar), 3.09 (d, *J* = 13.3 Hz, 1H, NCH_2Ar), 2.77 (dd, *J* = 10.8, 2.5 Hz, 1H, H-5a), 2.36–2.26 (m, 2H, H-2, H-5b), 1.75–1.54 (m, 2H, CH_2CH$_3$), 1.03 (t, *J* = 7.4 Hz, 3H, CH$_2$CH_3); ^{13}C NMR (100 MHz, $CDCl_3$) δ = 138.1 (Ar), 137.4 (Ar), 130.7 (Ar), 92.3 (Ar), 72.0 (C-3), 69.8 (C-4), 68.7 (C-2), 59.8 (C-5), 56.9 (NCH_2Ar), 20.6 (CH_2CH$_3$), 10.7 (CH$_2$$CH_3$) ppm; HRMS (ESI, m/z): calculated for $C_{13}H_{18}INO_2$ $[M+H]^+$: 348.0455; found: 348.0453.

Synthesis of alcohol 16 from trityl derivative 6

(3a*R*,4*S*,6a*S*)-Benzyl 2,2-dimethyl-4-(trityloxymethyl)dihydro-3a*H*-[1,3]dioxolo[4,5-*c*]pyrrole-5(4*H*)carboxylate (14). A suspension of imino-L-lyxitol **6** (16.10 g, 31.8 mmol) and 10% palladium on carbon (1.61 g, 10% by weight) in MeOH (340 mL) was stirred at rt in a hydrogen atmosphere (balloon) overnight. The catalyst was filtered off and the filtrate was evaporated. The residue (11.99 g, 28.8 mmol) was dissolved in CH_2Cl_2 (200 mL) and the resulting solution was treated with Et_3N (7.25 g, 10 mL, 71.6 mmol) followed by cooling in an ice-water bath to 0 °C. Next, benzyl chloroformate (45% in toluene, 9.84 g, 21.4 mL, 57.7 mmol) was added dropwise and the reaction mixture was allowed to stir at rt for 2 h. The reaction mixture was washed with water (2 × 200 mL), organic layer was dried (Na_2SO_4), filtered and the solvent was evaporated. The crude product was purified by column chromatography (EtOAc/hexane 1:4) to afford *N*-benzyloxycarbonyl derivative **14** as colorless oil (14.88 g, 85%) which crystallized in the frigde. M.p. 113–115 °C (Ref.[7] m.p. 105 °C); R_f = 0.20 (EtOAc/hexane 1:4); $[\alpha]_D^{20}$ = +32.1 (*c* = 1.0, $CHCl_3$) (Ref.[7] $[\alpha]_D^{20}$ = +30.9 (*c* = 1.2, $CHCl_3$)); IR (ATR) *v* = 1701, 1421, 1200, 748, 696 cm^{-1}; ^1H NMR (400 MHz, $CDCl_3$) δ = 7.52–7.45 (m, 5H, Ar_I), 7.36–7.19 (m, 15H, Ar), 5.19 (d, *J* = 12.3 Hz, 1H, OCH_2Ph), 5.03 (d, *J* = 12.3 Hz, 1H, OCH_2Ph), 4.83 (t, *J* = 6.7 Hz, 1H, H-3), 4.72 (td, *J* = 7.1, 4.3 Hz, 1H, H-2), 4.31 (q, *J* = 6.7 Hz, 1H, H-4), 3.96 (dd, *J* = 12.3, 7.1 Hz, 1H, H-1a), 3.42 (br t, *J* = 8.1 Hz, 1H, H-5a), 3.34 (dd, *J* = 9.1, 6.7 Hz, 1H, H-5b), 3.26 (dd, *J* = 12.3, 4.3 Hz, 1H, H-1b), 1.32 and 1.27 [2s, each 3H, C(CH_3)$_2$] ppm; ^{13}C NMR (100 MHz, $CDCl_3$) δ = 154.7 (C=O), 144.1 (Ph_3), 136.5 (Ph), 128.8 (Ph_3), 128.4 (Ph), 128.0 (Ph), 127.9 (Ph), 127.6 (Ph_3), 126.8 (Ph_3), 113.1 [C(CH$_3$)$_2$], 86.8 (CPh$_3$), 79.5 (C-3), 77.7 (C-2), 67.0 (OCH_2Ph), 61.3 (C-5), 58.8 (C-4), 50.4 (C-1), 26.1 and 25.0 [C(CH_3)$_2$] ppm; HRMS (ESI, m/z): calculated for $C_{35}H_{35}NO_5$ $[M+Na]^+$: 572.2407; found: 572.2392.

(3a*R*,4*S*,6a*S*)-Benzyl 4-(hydroxymethyl)-2,2-dimethyldihydro-3a*H*-[1,3]dioxolo[4,5-*c*]pyrrole-5(4*H*)carboxylate (16). A solution of trityl ether **14** (14.88 g, 27.7 mmol) in a mixture of MeOH/CH_2Cl_2 (1:30, (v/v), 280 mL) and *p*-toluenesulfonic acid monohydrate (206 mg, 1.08 mmol) was stirred for 20 min at rt. Next, *p*-toluenesulfonic acid was neutralized with concd. aqueous NH_3 (2 mL). The resulting mixture was dried (Na_2SO_4), filtered and the solvent was evaporated. The crude product was purified by column chromatography (EtOAc/hexane 1:1) to afford alcohol **16** as colorless oil (7.59 g, 92%). R_f = 0.24 (EtOAc/hexane 1:1); $[\alpha]_D^{20}$ = +35.2 (*c* = 0.93, $CHCl_3$) (Ref.[8] $[\alpha]_D^{25}$ = +33.0 (*c* = 1.00, $CHCl_3$)); IR (ATR) *v* = 3379, 1682, 1417, 1210, 1083, 1026 cm^{-1}; ^1H NMR (400 MHz, $CDCl_3$) δ = 7.44–7.29 (m, 5H, Ph), 5.17 (d, *J* = 12.4 Hz, 1H, OC*H*$_2$Ph), 5.13 (d, *J* = 12.3 Hz, 1H, OC*H*$_2$Ph), 4.82 (br s, 1H), 4.73 (br s, 1H), 4.38 (br s, 1H, OH), 3.91 (br s, 3H), 3.65 (br s, 2H), 1.50 and 1.34 [2s, each 3H, C(C*H*$_3$)$_2$] ppm; ^{13}C NMR (100 MHz, $CDCl_3$) δ = 156.3 (C=O), 136.1 (Ph), 128.5 (Ph), 128.2 (Ph), 128.0 (Ph), 112.4 [*C*(CH_3)$_2$], 80.4, 77.6, 67.4 (O*C*H_2Ph), 63.5, 62.1, 51.9, 26.3 and 24.7 [C(*C*H_3)$_2$] ppm; HRMS (ESI, m/z): calculated for $C_{16}H_{21}NO_5$ $[M+H]^+$: 308.1492; found: 308.1490.

Synthesis of alcohol 16 from silyl derivative 13

(3a*R*,4*S*,6a*S*)-Benzyl 4-((*tert*-butyldimethylsilyloxy)methyl)-2,2-dimethyldihydro-3a*H*-[1,3]dioxolo[4,5-*c*]pyrrole-5(4*H*)-carboxylate (15). A suspension of imino-L-lyxitol **13** (6.18 g, 16.4 mmol) and 10% palladium on carbon (0.62 g, 10% by weight) in MeOH (150 mL) was stirred at rt in a hydrogen atmosphere (balloon) for 4h. The catalyst was then filtered off and washed with MeOH (50 mL). The solvent was evaporated and the residue (4.70 g, 16.3 mmol) was dissolved in CH_2Cl_2 (60 mL) and the resulting solution was treated with Et_3N (6.76 g, 9.31 mL, 66.8 mmol) followed by cooling in an ice-water bath to 0 °C. Next, benzyl chloroformate (7.83 g, 6.55 mL, 45.9 mmol) was added dropwise and the reaction mixture was allowed to stir at rt for 4 h. The reaction mixture was washed with water (2×100 mL), organic layer was dried (Na_2SO_4), filtered and the solvent was evaporated. The crude product was purified by column chromatography (hexane/EtOAc 40:1→15:1) to afford **15** as colorless oil (5.39 g, 78%). R_f = 0.35 (EtOAc/hexane 1:3); $[\alpha]_D^{20}$ = −15.4 (*c* = 0.25, $CHCl_3$); IR (ATR) *v* = 1706, 1410, 1211, 1085, 836 cm^{-1}; ^1H NMR (400 MHz, $CDCl_3$) δ = 7.36–7.30 (m, 5H, Ph.), 5.12 (d, *J* = 12.4 Hz, 1H, OC*H*$_2$Ph), 5.08 (d, *J* = 12.4 Hz, 1H, OC*H*$_2$Ph), 4.76 (t, *J* = 6.0 Hz, 1H, H-3), 4.71-4.66 (m, 1H, H-2), 4.04-3.99 (m, 2H, H-4, H-5a), 3.90–3.81 (m, 2H, H-1a, H-5b), 3.36 (dd, *J* = 4.7 Hz, *J* = 12.1, 1H, H-1b), 1.50 and 1.33 [2s, each 3H, C(C*H*$_3$)$_2$], 0.88 [br s, 9H, (CH_3)$_3$], 0.03 [s, 6H, Si(CH_3)$_2$] ppm; ^{13}C NMR (100 MHz, $CDCl_3$) δ = 155.2 (C=O), 136.7 (Ph), 128.4 (Ph), 128.2 (Ph), 128.1 (Ph), 112.8 (*C*(CH_3)$_2$), 79.8 (C-3), 77.4 (C-2), 67.2 (O*C*H_2Ph), 61.1 (C-4), 60.4 (C-5), 51.9 (C-1), 27.1 [C(*C*H_3)$_2$], 26.0 and 25.4 [C(*C*H_3)$_2$], 18.5 [*C*(CH_3)$_3$], −5.2 and −5.3 [Si(CH_3)$_2$] ppm; HRMS (ESI, m/z): calculated for $C_{22}H_{35}NO_5Si$ $[M+H]^+$: 422.2357; found: 422.2357.

(3a*R*,4*S*,6a*S*)-Benzyl 4-(hydroxymethyl)-2,2-dimethyldihydro-3a*H*-[1,3]dioxolo[4,5-*c*]pyrrole-5(4*H*)-carboxylate (16). Silyl ether **15** (5.30 g, 12.6 mmol) was dissolved in THF (75 mL), cooled down in an ice-water bath to 0 °C followed by the addition of 1M TBAF in THF (4.93g, 18.86 mL, 18.8 mmol). The reaction mixture was stirred at rt for 2h. Then it was poured into cold satd. $NaHCO_3$/EtOAc (1:1, 200 mL), organic layer was separated, aqueous layer was washed with EtOAc (3×50 mL), the organic phases were combined, dried (Na_2SO_4) and concentrated. The purification of the crude product by column chromatography (hexane/EtOAc 3:1→1:1) afforded alcohol **16** as colorless oil (3.17 g, 82%). All spectral characteristics and properties were in accordance with **16** prepared from trityl derivative **6**.

(3a*R*,4*S*,6a*S*)-Benzyl 4-((*R*)-1-hydroxyethyl)-2,2-dimethyldihydro-3a*H*-[1,3]dioxolo[4,5-*c*]pyrrole-5(4*H*)-carboxylate (18). To a stirred solution of alcohol **16** (1.25 g, 4.07 mmol) in CH_2Cl_2 (50 mL), Dess-Martin periodinane (2.59 g, 6.10 mmol) was added and stirring was continued for 1 h at rt. The reaction mixture was washed twice with a mixture of satd. $NaHCO_3$ solution (25 mL) and 25% solution of $Na_2S_2O_3$ (25 mL). Organic layer was separated, dried (Na_2SO_4), filtered and the solvent was evaporated. Crude aldehyde **17** (1.24 g, 4.06 mmol) was dissolved in dry Et_2O (55 mL) and the resulting solution was cooled to 0 °C in an ice-water bath. Next, MeMgBr (4.7 mL, 14.21 mmol, 3 M in ether) was added dropwise at 0 °C. The ice-water bath was removed and the reaction mixture was stirred for 1–1.5 h at rt. Then, the reaction mixture was cooled in the ice-water bath and carefully quenched with satd. solution of NH_4Cl (20 mL). To the resulting mixture was added water (30 mL), organic layer was separated and the aqueous layer was extracted with additional diethyl ether (30 mL). Combined extracts were dried (Na_2SO_4), filtered and the solvent was evaporated. The crude product was purified by column chromatography (EtOAc/hexane 1:2) to afford alcohol **18** as colorless

oil (0.90 g, 69%). R_f = 0.22 (EtOAc/hexane1:2); $[\alpha]_D^{20}$ = +8.1 (*c* = 0.67 $CHCl_3$); IR (ATR) *v* = 3366, 1679, 1418, 1210, 1026 cm^{-1}; ^1H NMR (400 MHz, $CDCl_3$) δ = 7.45–7.29 (m, 5H, Ph), 5.16 (s, 2H, OCH_2Ph), 4.75–4.61 (m, 2H), 4.24 (pd, *J* = 6.4, 3.7 Hz, 1H, H-5), 3.74–3.64 (m, 3H), 1.49 and 1.34 [2s, each 3H, C(CH_3)$_2$], 1.36 (d, *J* = 6.4 Hz, 3H, CH_3) ppm; ^{13}C NMR (100 MHz, $CDCl_3$) δ = 156.4 (C=O), 136.2 (Ph), 128.5 (Ph), 128.1 (Ph), 127.9 (Ph), 112.4 [$C(CH_3)_2$], 80.3, 77.0, 67.5 (OCH_2Ph), 66.6, 52.5 (2×C), 26.8 and 24.9 [C(CH_3)$_2$], 20.4 (CH_3) ppm; HRMS (ESI, m/z): calculated for $C_{17}H_{23}NO_5$ $[M+H]^+$: 322.1649; found: 322.1636.

General procedure for the preparation of compounds 19
A suspension of benzyl carbamate **18** (170 mg, 0.53 mmol) and 10% palladium on carbon (17 mg, 10% by weight) in MeOH (9 mL) was stirred at rt in a hydrogen atmosphere (balloon) for 2 h. The catalyst was filtered off and the filtrate was evaporated at reduced pressure. The residue (90 mg) was dissolved in anhydrous DMF (4.5 mL), anhydrous K_2CO_3 (93 mg, 0.67 mmol) was added followed by corresponding benzyl bromide (1 equiv) while cooling in an ice-water bath. The reaction mixture was stirred at rt overnight. Then, the mixture was partitioned between ethyl acetate (20 mL) and water (20 mL). Organic layer was washed with water (2×20 mL), dried (Na_2SO_4), filtered and the solvent was evaporated. The residue was purified by column chromatography (MeOH/$CHCl_3$ 1:60).

(*R*)-1-((3a*R*,4*S*,6a*S*)-5-Benzyl-2,2-dimethyltetrahydro-3a*H*-[1,3]dioxolo[4,5-*c*]pyrrol-4-yl)ethanol (19a). The reaction was carried out according to general procedure with benzyl bromide (63 µL, 91 mg, 0.53 mmol) to afford pyrrolidine **19a** as colorless oil (65 mg, 42%). R_f = 0.16 (MeOH/$CHCl_3$ 1:60); $[\alpha]_D^{20}$ = +59.7 (*c* = 0.84, $CHCl_3$); IR (ATR) *v* = 3344, 2938, 1374, 1099, 1026 cm^{-1}; ^1H NMR (400 MHz, $CDCl_3$) δ = 7.42–7.21 (m, 5H, Ph), 4.67 (dd, *J* = 6.5, 4.8 Hz, 1H, H-3), 4.58 (dd, *J* = 6.5, 4.9 Hz, 1H, H-2), 4.36 (d, *J* = 13.3 Hz, 1H, NCH_2Ph), 4.24 (p, *J* = 6.3 Hz, 1H, H-5), 3.22 (d, *J* = 13.3 Hz, 1H, NCH_2Ph), 3.07 (d, *J* = 11.4 Hz, 1H, H-1a), 2.58 (s, 1H), 2.36 (t, *J* = 4.9 Hz, 1H, H-4), 2.16 (dd, *J* = 11.4, 4.9 Hz, 1H, H-1b), 1.56 and 1.32 [2s, each 3H, C(CH_3)$_2$], 1.44 (d, *J* = 6.6 Hz, 3H, CH_3) ppm; ^{13}C NMR (100 MHz, $CDCl_3$) δ = 138.9 (Ph), 128.4 (Ph), 128.3 (Ph), 126.9 (Ph), 111.2 [$C(CH_3)_2$], 81.8 (C-3), 77.7 (C-2), 71.7 (C-4), 66.5 (C-5), 59.1 (C-1), 58.7 (NCH_2Ph), 26.2 and 24.9 [C(CH_3)$_2$], 22.2 (CH_3) ppm; HRMS (ESI, m/z): calculated for $C_{16}H_{23}NO_3$ $[M+H]^+$: 278.1751; found: 278.1745.

(*R*)-1-((3a*R*,4*S*,6a*S*)-5-(4-Bromobenzyl)-2,2-dimethyltetrahydro-3a*H*-[1,3]dioxolo[4,5-*c*]pyrrol-4-yl)ethanol (19b). The reaction was carried out according to general procedure with 4-bromobenzyl bromide (132 mg, 0.53 mmol) to afford pyrrolidine **19b** as colorless oil (126 mg, 67%). R_f = 0.17 (MeOH/$CHCl_3$ 1:60); $[\alpha]_D^{20}$ = +53.6 (*c* = 0.66, $CHCl_3$); IR (ATR) *v* = 3439, 2933, 1330, 1206, 1098, 1011 cm^{-1}; ^1H NMR (400 MHz, $CDCl_3$) δ = 7.43 (d, *J* = 8.3 Hz, 2H, Ar), 7.25 (d, *J* = 8.3 Hz, 2H, Ar), 4.64 (dd, *J* = 6.5, 4.8 Hz, 1H, H-3), 4.56 (dd, *J* = 6.5, 4.8 Hz, 1H, H-2), 4.33 (d, *J* = 13.5 Hz, 1H, NCH_2Ar), 4.22 (qd, *J* = 6.5, *J* = 5.1 Hz, 1H, H-5), 3.15 (d, *J* = 13.5 Hz, 1H, NCH_2Ar), 3.01 (d, *J* = 11.3 Hz, 1H, H-1a), 2.31 (t, *J* = 5.0 Hz, 1H, H-4), 2.10 (dd, *J* = 11.3, 4.8 Hz, 1H, H-1b), 1.53 and 1.30 [2s, each 3H, C(CH_3)$_2$], 1.42 (d, *J* = 6.6 Hz, 3H, CH_3) ppm; ^{13}C NMR (100 MHz, $CDCl_3$) δ = 138.1 (Ar), 131.3 (Ar), 130.0 (Ar), 120.6 (Ar), 111.1 [$C(CH_3)_2$], 81.7 (C-3), 77.5 (C-2), 71.7 (C-4), 66.8 (C-5), 59.2 (C-1), 57.9 (NCH_2Ar), 26.2 and 24.9 [C(CH_3)$_2$], 22.2 (CH_3) ppm; HRMS (ESI, m/z): calculated for $C_{16}H_{22}BrNO_3$ $[M+H]^+$: 356.0856; found: 356.0857.

(*R*)-1-((3a*R*,4*S*,6a*S*)-5-(4-Iodobenzyl)-2,2-dimethyltetrahydro-3a*H*-[1,3]dioxolo[4,5-*c*]pyrrol-4-yl)ethanol (19c). The reaction was carried out according to general procedure with 4-iodobenzyl bromide (157 mg, 0.53 mmol) to afford pyrrolidine **19c** as colorless oil (140 mg, 76%). R_f = 0.16 (MeOH/$CHCl_3$ 1:60); $[\alpha]_D^{20}$ = +182.8 (*c* = 0.10, $CHCl_3$); IR (ATR) *v* = 3426, 2930, 1370, 1207, 1101, 1007 cm^{-1}; ^1H NMR (400 MHz, $CDCl_3$) δ = 7.63 (d, *J* = 8.3 Hz, 2H, Ar), 7.11 (d, *J* = 8.2 Hz, 2H, Ar), 4.63 (dd, *J* = 4.8 Hz, *J* = 6.5 Hz, 1H, H-3), 4.55 (dd, *J* = 4.8 Hz, *J* = 6.4 Hz, 1H, H-2), 4.32 (d, *J* = 13.5 Hz, 1H, NCH_2Ar), 4.22 (qd, *J* = 5.3 Hz, *J* = 6.6 Hz, 1H, H-5), 3.17 (d, *J* = 13.5 Hz, 1H, NCH_2Ar), 3.01 (d, *J* = 11.3 Hz, 1H, H-1a), 2.33 (t, *J* = 5.0 Hz, 1H, H-4), 2.11 (dd, *J* = 4.8 Hz, *J* = 11.4 Hz, 1H, H-1b), 1.52 and 1.29 [2s, each 3H, C(CH_3)$_2$], 1.40 (d, *J* = 6.6 Hz, 3H, CH_3) ppm; ^{13}C NMR (100 MHz, $CDCl_3$) δ = 138.8 (Ar), 137.6 (Ar), 130.7 (Ar), 111.3 [$C(CH_3)_2$], 92.4 (Ar), 81.8 (C-3), 77.7 (C-2), 71.9 (C-4), 66.9 (C-5), 59.3 (C-1), 58.1 [NCH_2Ar], 26.4 and 25.1 [C(CH_3)$_2$], 22.4 (CH_3) ppm; HRMS (ESI, m/z): calculated for $C_{16}H_{22}INO_3$ $[M+H]^+$: 404.0717; found: 404.0711.

General procedure for the preparation of compounds 4
To a stirred solution of corresponding acetonide **19** in MeOH (1 mL/~30 mg of acetonide), 20% HCl (0.5 mL) was added while cooling in an ice-water bath. After 15 min of stirring, the ice-water bath was removed and the stirring was continued at rt for 48 h. Then, HCl was carefully neutralized with solid

Na_2CO_3 (1.5 equiv regarding HCl used). The resulting suspension was filtered and the filtrate was concentrated. The residue was suspended in MeOH (5 mL), filtered and the filtrate was evaporated to dryness. The residue was purified by column chromatography (MeOH/$CHCl_3$ 1:20 containing 0.5% (v/v) of concd. aqueous NH_3).

(2*S*,3*R*,4*S*)-1-Benzyl-2-((*R*)-1-hydroxyethyl)pyrrolidine-3,4-diol (4a). The reaction was carried out according to general procedure with acetonide **19a** (27 mg, 0.097 mmol) to afford triol **4a** as colorless oil (17 mg, 72%). R_f = 0.27 (MeOH/$CHCl_3$ 1:10 containing 0.5% (v/v) of concd. aqueous NH_3); $[\alpha]_D^{20}$ = +24.4 (*c* = 0.30, MeOH); IR (ATR) *v* = 3334, 2928, 1452, 1119, 1027 cm^{-1}; ^1H NMR (400 MHz, CD_3OD) δ = 7.34–7.19 (m, 5H, Ph), 4.20 (dd, *J* = 7.2, 4.8 Hz, 1H, H-3), 4.18 (d, *J* = 13.4 Hz, 1H, NCH_2Ph), 4.06 (dq, *J* = 6.5, 4.6 Hz, 1H, H-5), 4.01 (td, *J* = 5.4, 3.9 Hz, 1H, H-2), 3.57 (d, *J* = 13.4 Hz, 1H, NCH_2Ph), 2.87 (dd, *J* = 10.9, 3.9 Hz, 1H, H-1a), 2.82 (dd, *J* = 7.2, 4.6 Hz, 1H), 2.58 (dd, *J* = 10.9, 5.4 Hz, 1H, H-1b), 1.29 (d, *J* = 6.5 Hz, 3H, CH_3) ppm; ^{13}C NMR (100 MHz, CD_3OD) δ = 140.5 (Ph), 129.9 (Ph), 129.2 (Ph), 128.0 (Ph), 74.0 (C-3), 72.2 (C-2), 71.3 (C-4), 67.7 (C-5), 63.1 (NCH_2Ph), 58.8 (C-1), 21.4 (CH_3) ppm; HRMS (ESI, m/z): calculated for $C_{13}H_{19}NO_3$ $[M+H]^+$: 238.1438; found: 238.1436.

(2*S*,3*R*,4*S*)-1-(4-Bromobenzyl)-2-((*R*)-1-hydroxyethyl)pyrrolidine-3,4-diol (4b). The reaction was carried out according to general procedure with acetonide **19b** (41 mg, 0.11 mmol) to afford triol **4a** as thick colorless oil (20 mg, 55%) which slowly crystallized in a refrigerator. M.p. 86–88 °C; R_f = 0.14 (MeOH/$CHCl_3$ 1:20 containing 0.5% (v/v) of concd. aqueous NH_3); $[\alpha]_D^{20}$ = +43.4 (*c* = 0.23, MeOH); IR (ATR) *v* = 3292, 1485, 1128, 1067, 1008 cm^{-1}; ^1H NMR (400 MHz, CD_3OD) δ = 7.45 (d, *J* = 8.3 Hz, 2H, Ar), 7.33 (d, *J* = 8.3 Hz, 2H, Ar), 4.20 (dd, *J* = 7.1, 4.7 Hz, 1H, H-3), 4.16 (d, *J* = 13.7 Hz, 1H, NCH_2Ar), 4.10–4.00 (m, 2H, H-2, H-5), 3.54 (d, *J* = 13.7 Hz, 1H, NCH_2Ar), 2.87 (dd, *J* = 10.8, 4.0 Hz, 1H, H-1a), 2.80 (dd, *J* = 7.1, 4.7 Hz, 1H, H-4), 2.54 (dd, *J* = 10.8, 5.5 Hz, 1H, H-1b), 1.28 (d, *J* = 6.5 Hz, 3H, CH_3) ppm; ^{13}C NMR (100 MHz, CD_3OD) δ = 140.1 (Ar), 132.3 (Ar), 131.6 (Ar), 121.5 (Ar), 74.0 (C-3), 72.1 (C-2), 71.4 (C-4), 67.9 (C-5), 62.4 (NCH_2Ar), 58.9 (C-1), 21.3 (CH_3) ppm; HRMS (ESI, m/z): calculated for $C_{13}H_{18}BrNO_3$ $[M+H]^+$: 316.0543; found: 316.0545.

(2*S*,3*R*,4*S*)-1-(4-Iodobenzyl)-2-((*R*)-1-hydroxyethyl)pyrrolidine-3,4-diol (4c). The reaction was carried out according to general procedure with acetonide **19c** (140 mg, 0.35 mmol) to afford triol **4c** as yellowish oil (103 mg, 82%). R_f = 0.14 (MeOH/$CHCl_3$ 1:20 containing 0.5% (v/v) of concd. aqueous NH_3); $[\alpha]_D^{20}$ = +39.0 (*c* = 0.21, MeOH); IR (ATR) *v* = 3336, 2926, 1482, 1121, 1006 cm^{-1}; ^1H NMR (400 MHz, CD_3OD) δ = 7.67 (d, *J* = 8.3 Hz, 2H, Ar), 7.22 (d, *J* = 8.2 Hz, 2H, Ar), 4.23 (dd, *J* = 4.7 Hz, *J* = 7.1 Hz, 1H, H-3), 4.17 (d, *J* = 13.8 Hz, 1H, NCH_2Ar), 4.10-4.04 (m, 2H, H-2, H-5), 3.55 (d, *J* = 13.8 Hz, 1H, NCH_2Ar), 2.89 (dd, *J* = 3.9 Hz, *J* = 10.8 Hz, 1H, H-1a), 2.82 (dd, *J* = 4.6 Hz, *J* = 7.1 Hz, 1H, H-4), 2.55 (dd, *J* = 5.4 Hz, *J* = 10.8 Hz, 1H, H-1b), 1.30 (d, *J* = 6.5 Hz, 3H, CH_3) ppm; ^{13}C NMR (100 MHz, CD_3OD) δ = 140.7 (Ar), 138.4 (Ar), 131.9 (Ar), 92.7 (Ar), 74.0 (C-3), 72.2 (C-2), 71.4 (C-4), 67.9 (C-5), 62.6 (NCH_2Ar), 59.0 (C-1), 21.3 (CH_3) ppm; HRMS (ESI, m/z): calculated for $C_{13}H_{18}INO_3$ $[M+H]^+$: 364.0404; found: 364.0399.

(3a*R*,3b*S*,4*S*,8a*S*)-4-Methyl-2,2-dimethyltetrahydro-4*H*,6*H*-[1,3]dioxolo[4',5':3,4]pyrrolo[1,2-*c*]oxazol-6-one (20). To a solution of alcohol **18** (502 mg, 1.56 mmol) and pyridine (0.31 mL, 3.90 mmol) in anhydrous CH_2Cl_2 (10 mL), triflic anhydride (0.53 mL, 3.13 mmol) was added dropwise while cooling in an ice-water bath to 0 °C. The reaction mixture was allowed to stir in the ice-water bath at 0 °C for 1.5 h. Next, the mixture was diluted with $CHCl_3$ (25 mL) and was washed with water (25 mL). Aqueous layer was extracted with $CHCl_3$ (25 mL). Combined extracts were dried (Na_2SO_4), filtered and the solvent was evaporated. The crude product was purified by column chromatography (EtOAc/hexane 2:1) to afford carbamate **20** as white crystals (228 mg, 72%). M.p. 99–101 °C (EtOAc/hexanes 1:2); R_f = 0.22 (EtOAc/hexane2:1); $[\alpha]_D^{20}$ = +9.1 (*c* = 0.21 $CHCl_3$); IR (ATR) *v* = 1740, 1392, 1209, 1070, 1037 cm^{-1}; ^1H NMR (400 MHz, $CDCl_3$) δ = 4.86 (p, *J* = 6.8 Hz, 1H, H-4), 4.79 (td, *J* = 5.3, 1.3 Hz, 1H, H-8), 4.61 (dd, *J* = 5.1, 3.3 Hz, 1H, H-3a), 3.87 (dd, *J* = 13.3, 1.3 Hz, 1H, H-7'), 3.64 (dd, *J* = 6.9, 3.3 Hz, 1H, H-3b), 3.13 (dd, *J* = 13.3, 5.5 Hz, 1H, H-7'), 1.65 (d, *J* = 6.6 Hz, 3H, CH_3), 1.46 and 1.28 [2s, each 3H, $C(CH_3)_2$] ppm; ^{13}C NMR (100 MHz, $CDCl_3$) δ = 161.1 (C=O), 112.8 [$C(CH_3)_2$], 81.8 (C-8), 79.9 (C-3a), 72.1 (C-4), 65.8 (C-3b), 52.5 (C-7), 26.5 and 24.4 [$C(CH_3)_2$], 14.5 (CH_3) ppm; HRMS (ESI, m/z): calculated for $C_{10}H_{15}NO_4$ $[M+H]^+$: 214.1074; found: 214.1073.

(*S*)-1-((3a*R*,4*S*,6a*S*)-2,2-Dimethyltetrahydro-3a*H*-[1,3]dioxolo[4,5-*c*]pyrrol-4-yl)ethanol (21). A solution of carbamate **20** (228 mg, 1.12 mmol) in EtOH (20 mL) and 10% NaOH (4 mL) was heated under reflux for 24 h. After cooling down, the ethanol was evaporated and the residue was partitioned

between $CHCl_3$ (20 mL) and brine (20 mL). Aqueous layer was extracted with additional $CHCl_3$ (20 mL). Combined extracts were dried (Na_2SO_4), filtered and the solvent was evaporated. The crude product was purified by column chromatography (MeOH/$CHCl_3$ 1:20 containing 0.5% (v/v) of concd. aqueous NH_3) to afford pyrrolidine **21** as white crystals (155 mg, 74%). M.p. 107–109 °C; R_f = 0.14 (MeOH/$CHCl_3$ 1:20 containing 0.5% (v/v) of concd. aqueous NH_3); $[\alpha]_D^{20}$ = +73.5 (*c* = 0.60 $CHCl_3$); IR (ATR) *v* = 3249, 3185, 2975, 1367, 1088, 900 cm^{-1}; ^1H NMR (400 MHz, $CDCl_3$) δ = 4.75 (dd, *J* = 5.6, 4.1 Hz, 1H, H-3a), 4.69 (dd, *J* = 5.6, 3.8 Hz, 1H, H-6a), 4.04 (td, *J* = 6.6, 5.1 Hz, 1H, C*H*OH), 3.15 (d, *J* = 13.4 Hz, 1H, H-6), 2.65 (dd, *J* = 13.4, 3.8 Hz, 1H, H-6'), 2.56 (dd, *J* = 5.2, 4.1 Hz, 1H, H-4), 1.47 and 1.32 [2s, each 3H, C(CH_3)$_2$], 1.39 (d, *J* = 6.6 Hz, 3H, CH_3) ppm; ^{13}C NMR (100 MHz, $CDCl_3$) δ = 110.8 [*C*(CH_3)$_2$], 82.2 (C-6a), 82.1 (C-3a), 67.9 (C-4), 67.3 (*C*HOH), 52.5 (C-6), 25.6 and 23.5 [C(*C*H_3)$_2$], 21.5 (*C*H_3) ppm; HRMS (ESI, m/z): calculated for $C_9H_{17}NO_3$ $[M+H]^+$: 188.1281; found: 188.1282.

General procedure for the preparation of compounds 22

To a solution of pyrrolidine **21** (41 mg, 0.22 mmol) in anhydrous DMF (1.5 mL), anhydrous K_2CO_3 (51 mg, 0.37 mmol) was added followed by the addition of corresponding benzyl bromide (1.3 equiv) while cooling in an ice-water bath. The reaction mixture was stirred at rt overnight. Then, the mixture was partitioned between ethyl acetate (12 mL) and water (12 mL). Organic layer was washed with water (2×12 mL), dried (Na_2SO_4), filtered and the solvent was evaporated. Crude products were purified by column chromatography (MeOH/$CHCl_3$ 1:60).

(*S*)-1-((3a*R*,4*S*,6a*S*)-5-Benzyl-2,2-dimethyltetrahydro-3a*H*-[1,3]dioxolo[4,5-*c*]pyrrol-4-yl)ethanol (22a). The reaction was carried out according to general procedure using benzyl bromide (34 µL, 49 mg, 0.28 mmol) to afford benzylpyrrolidine derivative **22a** as colorless oil (52 mg, 86%). R_f = 0.25 (MeOH/$CHCl_3$ 1:60); $[\alpha]_D^{20}$ = +95.9 (*c* = 0.14, $CHCl_3$); IR (ATR) *v* = 3517, 2932, 1371, 1207, 1099, 1079 cm^{-1}; ^1H NMR (400 MHz, $CDCl_3$) δ = 7.38–7.23 (m, 5H, Ph), 4.79 (dd, *J* = 6.4, 4.2 Hz, 1H, H-3a), 4.56 (dd, *J* = 6.4, 5.0 Hz, 1H, H-6a), 4.26 (m, 1H, C*H*OH), 4.23 (d, *J* = 13.1 Hz, 1H, NCH_2Ph), 3.14 (d, *J* = 13.1 Hz, 1H, NCH_2Ph), 3.06 (d, *J* = 11.0 Hz, 1H, H-6), 2.13–2.05 (m, 2H, H-4, H-6'), 1.56 and 1.32 [2s, each 3H, C(CH_3)$_2$], 1.46 (d, *J* = 6.6 Hz, 3H, CH_3) ppm; ^{13}C NMR (100 MHz, $CDCl_3$) δ = 137.9 (Ph), 128.8 (Ph), 128.2 (Ph), 126.9 (Ph), 111.2 [*C*(CH_3)$_2$], 81.1 (C-3a), 77.2 (C-6a), 70.1 (C-4), 64.3 (*C*HOH), 58.7 (C-6), 55.7 (N*C*H_2Ph), 26.2 and 24.7 [C(*C*H_3)$_2$], 20.6 (*C*H_3) ppm; HRMS (ESI, m/z): calculated for $C_{16}H_{23}NO_3$ $[M+H]^+$: 278.1751; found: 278.1742.

(*S*)-1-((3a*R*,4*S*,6a*S*)-5-(4-Bromobenzyl)-2,2-dimethyltetrahydro-3a*H*-[1,3]dioxolo[4,5-*c*]pyrrol-4-yl)ethanol (22b). The reaction was carried out according to general procedure using 4-bromobenzyl bromide (71 mg, 0.28 mmol) to afford (4-bromobenzyl)pyrrolidine derivative **22b** as colorless oil (66 mg, 85%). R_f 0.28 (MeOH/$CHCl_3$ 1:60); $[\alpha]_D^{20}$ = +89.9 (*c* = 0.24, $CHCl_3$); IR (ATR) *v* = 3363, 2942, 1381, 1101, 1012 cm^{-1}; ^1H NMR (400 MHz, $CDCl_3$) δ = 7.43 (d, *J* = 8.3 Hz, 2H, Ar), 7.22 (d, *J* = 8.3 Hz, 2H, Ar), 4.78 (dd, *J* = 6.4, 4.1 Hz, 1H, H-3a), 4.55 (dd, *J* = 6.4, 4.9 Hz, 1H, H-6a), 4.25–4.11 (br s, 1H, C*H*OH), 4.15 (d, *J* = 13.1 Hz, 1H, NCH_2Ar), 3.44 (br s, 1H, OH), 3.02 (d, *J* = 13.1 Hz, 1H, NCH_2Ar), 3.00 (d, *J* = 10.9 Hz, 1H, H-6), 2.08 (t, *J* = 4.1 Hz, 1H, H-4), 2.04 (dd, *J* = 10.9, 4.9 Hz, 1H, H-6'), 1.53 and 1.31 [2s, each 3H, C(CH_3)$_2$], 1.44 (d, *J* = 6.6 Hz, 3H, CH_3); ^{13}C NMR (100 MHz, $CDCl_3$) δ = 137.3 (Ar), 131.3 (Ar), 130.4 (Ar), 120.7 (Ar), 111.3 [*C*(CH_3)$_2$], 81.1 (C-3a), 77.2 (C-6a), 70.2 (C-4), 64.4 (*C*HOH), 58.8 (C-6), 55.1 (N*C*H_2Ph), 26.2 and 24.7 [C(*C*H_3)$_2$], 20.7 (*C*H_3); HRMS (ESI, m/z): calculated for $C_{16}H_{22}BrNO_3$ $[M+H]^+$: 356.0856; found: 356.0856.

(*S*)-1-((3a*R*,4*S*,6a*S*)-5-(4-Iodobenzyl)-2,2-dimethyltetrahydro-3a*H*-[1,3]dioxolo[4,5-*c*]pyrrol-4-yl)ethanol (22c). The reaction was carried out according to general procedure using 4-iodobenzyl bromide (83 mg, 0.28 mmol) to afford (4-iodobenzyl)pyrrolidine derivative **22c** as colorless oil (72 mg, 81 %). R_f 0.27 (MeOH/$CHCl_3$ 1:60); $[\alpha]_D^{20}$ = +34.4 (*c* = 0.47, $CHCl_3$); IR (ATR) *v* = 3543, 2917, 1373, 1141, 1077, 1006 cm^{-1}; ^1H NMR (400 MHz, $CDCl_3$) δ = 7.67 (d, *J* = 8.1 Hz, 2H, Ar), 7.09 (d, *J* = 8.1 Hz, 2H, Ar), 4.77 (dd, *J* = 4.2 Hz, *J* = 6.4 Hz, 1H, H-3a), 4.54 (dd, *J* = 5.0 Hz, *J* = 6.3 Hz, 1H, H-6a), 4.23–4.14 (br s, 1H, C*H*OH), 4.14 (d, *J* = 13.2 Hz, 1H, NCH_2Ar), 3.43 (br s, 1H, OH), 3.02 (d, *J* = 13.2 Hz, 1H, NCH_2Ar), 2.99 (d, *J* = 10.8 Hz, 1H, H-6), 2.06 (t, *J* = 3.8 Hz, 1H, H-4), 2.03 (dd, *J* = 4.9 Hz, *J* = 10.9 Hz, 1H, H-6′), 1.52 and 1.30 [2s, each 3H, C(CH_3)$_2$], 1.42 (d, *J* = 6.6 Hz, 3H, CH_3); ^{13}C NMR (100 MHz, $CDCl_3$) δ = 138.2 (Ar), 137.4 (Ar), 130.9 (Ar), 111.5 [*C*(CH_3)$_2$], 92.4 (Ar), 81.2 (C-3a), 77.4 (C-6a), 70.4 (C-4), 64.6 (*C*HOH), 59.0 (C-6), 55.4 (N*C*H_2Ar), 26.4 and 24.9 [C(*C*H_3)$_2$], 20.9 (*C*H_3); HRMS (ESI, m/z): calculated for $C_{16}H_{22}INO_3$ $[M+H]^+$: 404.0717; found: 404.0716.

General procedure for the preparation of compounds 5

To a stirred solution of corresponding acetonide in MeOH (1 mL/~40 mg of acetonide), 20% HCl (0.5 mL/~40 mg of acetonide) was added while cooling in an ice-water bath. After 15 min of stirring, the ice-water bath was removed and the stirring was continued at rt for 48 h. Then, HCl was carefully neutralized with solid Na_2CO_3 (1.5 equiv regarding HCl used). The resulting suspension was filtered and the filtrate was evaporated to dryness. The residue was suspended in MeOH (5 mL), filtered and the filtrate was concentrated. The residue was purified by column chromatography (MeOH/$CHCl_3$ 1:20 containing 0.5% (v/v) of concd. aqueous NH_3).

(2*S*,3*R*,4*S*)-1-Benzyl-2-((*S*)-1-hydroxyethyl)pyrrolidine-3,4-diol (5a). The reaction was carried out according to general procedure with acetonide **22a** (40 mg, 0.14 mmol) to afford pyrrolidine **5a** as yellowish oil (20 mg, 60%). R_f 0.20 (MeOH/$CHCl_3$ 1:20 containing 0.5% (v/v) of concd. aqueous NH_3); $[\alpha]_D^{20}$ = +125.7 (*c* = 0.12, MeOH); IR (ATR) *v* = 3354, 2928, 1452, 1113, 1088, 1027 cm^{-1}; ^1H NMR (400 MHz, CD_3OD) δ = 7.41–7.16 (m, 5H, Ph), 4.33 (dd, *J* = 6.5, 4.8 Hz, 1H, H-3), 4.08 (dq, *J* = 6.6, 4.8 Hz, 1H, C*H*OH), 4.04 (m, 1H, H-4), 4.00 (d, *J* = 13.3 Hz, 1H, NCH_2Ph), 3.43 (d, *J* = 13.3 Hz, 1H, NCH_2Ph), 2.87 (dd, *J* = 10.7, 4.1 Hz, 1H, H-5), 2.63 (dd, *J* = 6.5, 4.8 Hz, 1H, H-2), 2.52 (dd, *J* = 10.7, 5.7 Hz, 1H, H-5'), 1.34 (d, *J* = 6.6 Hz, 3H, CH_3); ^{13}C NMR (100 MHz, CD_3OD) δ = 140.2 (Ph), 129.9 (Ph), 129.2 (Ph), 128.0 (Ph), 74.7 (C-3), 71.7 (C-4), 70.7 (C-2), 68.9 (*C*HOH), 60.7 (NCH_2Ph), 58.6 (C-5), 20.5 (CH_3); HRMS (ESI, m/z): calculated for $C_{13}H_{19}NO_3$ [*M*+H]$^+$: 238.1438; found: 238.1437.

(2*S*,3*R*,4*S*)-1-(4-Bromobenzyl)-2-((*S*)-1-hydroxyethyl)pyrrolidine-3,4-diol (5b). The reaction was carried out according to general procedure with acetonide **22b** (52 mg, 0.14 mmol) to afford pyrrolidine **5b** as yellowish solid (32 mg, 70%). M.p. 80–82 °C; R_f = 0.27 (MeOH/$CHCl_3$ 1:20 containing 0.5% (v/v) of concd. aqueous NH_3); $[\alpha]_D^{20}$ = +49.5 (*c* = 0.36, MeOH); IR (ATR) *v* = 3444, 3278, 1336, 1101, 1008 cm^{-1}; ^1H NMR (400 MHz, CD_3OD) δ = 7.44 (d, *J* = 8.3 Hz, 2H, Ar), 7.29 (d, *J* = 8.3 Hz, 2H, Ar), 4.34 (dd, *J* = 6.5, 4.8 Hz, 1H, H-3), 4.12–4.02 (m, 2H, H-4, C*H*OH), 3.97 (d, *J* = 13.6 Hz, 1H, NCH_2Ar), 3.39 (d, *J* = 13.6 Hz, 1H, NCH_2Ar), 2.86 (dd, *J* = 10.6, 4.0 Hz, 1H, H-5), 2.62 (dd, *J* = 6.5, 4.8 Hz, 1H, H-2), 2.47 (dd, *J* = 10.6, 5.7 Hz, 1H, H-5'), 1.33 (d, *J* = 6.6 Hz, 3H, CH_3) ppm; ^{13}C NMR (100 MHz, CD_3OD) δ = 139.8 (Ar), 132.3 (Ar), 131.6 (Ar), 121.6 (Ar), 74.7 (C-3), 71.8 (C-4), 70.7 (C-2), 68.9 (*C*HOH), 60.0 (NCH_2Ar), 58.7 (C-5), 20.5 (CH_3) ppm; HRMS (ESI, m/z): calculated for $C_{13}H_{18}BrNO_3$ [*M*+H]$^+$: 316.0543; found: 316.0545.

(2*S*,3*R*,4*S*)-1-(4-Iodobenzyl)-2-((*S*)-1-hydroxyethyl)pyrrolidine-3,4-diol (5c). The reaction was carried out according to general procedure with acetonide **22c** (60 mg, 0.15 mmol) to afford pyrrolidine **5c** as yellowish oil (38 mg, 70%). R_f = 0.27 (MeOH/$CHCl_3$ 1:20 containing 0.5% (v/v) of concd. aqueous NH_3); $[\alpha]_D^{20}$ = +52.3 (*c* = 0.26, MeOH); IR (ATR) *v* = 3331, 2943, 1449, 1414, 1022 cm^{-1}; ^1H NMR (400 MHz, CD_3OD) δ = 7.68 (d, *J* = 8.2 Hz, 2H, Ar), 7.19 (d, *J* = 8.2 Hz, 2H, Ar), 4.36 (dd, *J* = 4.9 Hz, *J* = 6.5 Hz, 1H, H-3), 4.14–4.06 (m, 2H, H-4, C*H*OH), 4.01 (d, *J* = 13.6 Hz, 1H, NCH_2Ar), 3.42 (d, *J* = 13.7 Hz, 1H, NCH_2Ar), 2.90 (dd, *J* = 4.1 Hz, *J* = 10.7 Hz, 1H, H-5), 2.66 (dd, *J* = 4.8 Hz, *J* = 6.4 Hz, 1H, H-2), 2.52 (dd, *J* = 5.7 Hz, *J* = 10.7 Hz, 1H, H-5′), 1.36 (d, *J* = 6.6 Hz, 3H, CH_3) ppm; ^{13}C NMR (100 MHz, CD_3OD) δ = 140.3 (Ar), 138.5 (Ar), 131.9 (Ar), 92.8 (Ar), 74.7 (C-3), 71.8 (C-4), 70.8 (C-2), 68.9 (*C*HOH), 60.1 (NCH_2Ar), 58.7 (C-5), 20.5 (CH_3) ppm; HRMS (ESI, m/z): calculated for $C_{13}H_{18}INO_3$ [*M*+H]$^+$: 364.0404; found: 364.0400.

Biochemical evaluation

The purification and characterization of recombinant *Drosophila melanogaster* Golgi α-mannosidase II (GMIIb) and lysosomal α-mannosidase II (LManII) was carried out as already described.[9] Activity of prepared mannosidases was measured using *p*-nitrophenyl-α-D-mannopyranoside (*p*NP-Man; Sigma-Aldrich) as a substrate at 2 mM concentration prepared from a 100 mM stock in dimethyl sulfoxide by dilution in 50 mM acetate buffer at optimal pH for the enzymes (Jack bean α-mannosidase (JBMan) at pH 5.0, Golgi α-mannosidase (GMIIb) at pH 6.0 and lysosomal α-mannosidase (LManII) at pH 5.2) and 0.5 µL of the enzyme (0.05 µg of protein for Jack bean α-mannosidase), in a total volume of 50 µL for 1–2 h at 37 °C. GMIIb was assayed in the presence of 0.5 mM $CoCl_2$. Pyrrolidines **2**–**5** used in biochemical evaluation were lyophilized before use. The inhibitors **2**–**5** were pre-incubated with the enzyme in the buffer for 5 min at rt and the reaction was started by addition of the substrate. The reactions were terminated with two volumes (0.1 mL) of 0.5 M sodium carbonate and the production of *p*-nitrophenol was measured at 405 nm using a multimode reader Mithras LB943 (Berthold Technologies). The IC_{50} value was determined with 2 mM *p*NP-Man. All biochemical assays were performed in 96 well microplates in triplicate.

References

1. Palatinus, L.; Chapuis, G. J. *Appl. Crystallogr*., **2007**, *40*, 786–790.
2. Sheldrick, G. M. *Acta Crystallogr. Sect. C*, **2015**, *A71*, 3–8.
3. Dolomanov, O. V.; Bourhis, L. J.; Gildea, R. J.; Howard, J. A. K.; Puschmann, H. *J. Appl. Crystallogr*., **2009**, *42*, 339–341.
4. Parsons, S.; Flack, H. D. *Acta Crystallogr. Sect. B*, **2013**, *B69*, 249–259.
5. Kim, D.-K.; Kim, G.; Kim, Y.-W. *J. Chem. Soc. Perkin Trans. 1*, **1996**, 803–808.
6. Díez, D.; Beneitez, M. T.; Marcos, I. S.; Garrido, N. M.; Basabe, P.; Urones, J. G. *Tetrahedron-Asymmetry*, **2002**, *13*, 639–646.
7. Ikota, N.; Inaba, H. *Chem. Pharm. Bull*., **1996**, *44*, 587–589.
8. Trajkovic, M.; Balanac, V.; Ferjancic, Z.; Saicic, R. N. *RSC Adv*., **2014**, *4*, 53722–53724.
9. Nemčovičová, **I**.; Šesták, S.; Rendic, D.; Plšková, M.; Mucha, J.; Wilson, **I**. B. H. *Glycoconjugate J*., **2013**, *30*, 899–909.
