# Supplementary material for: Synthesis of 1,4-imino-L-lyxitols modified at C-5 and their evaluation as inhibitors of GH38 α-mannosidases
Source: Beilstein J Org Chem. 2018 Aug 17;14:2156–62. doi: 10.3762/bjoc.14.189 (PMC6122390; doi:10.3762/bjoc.14.189)
Supplement: File 2 — Copies of 1H and 13C NMR spectra of all prepared compounds. [file Beilstein_J_Org_Chem-14-2156-s002.pdf]

Supporting Information

for

Synthesis of 1,4-imino-L-lyxitols modified at C-5 and their evaluation as inhibitors of GH38 α-mannosidases

Maroš Bella[1], Sergej Šesták[1], Ján Moncoľ[2], Miroslav Koóš[1] and Monika Poláková*[1]

Address: [1]Department of Glycochemistry, Institute of Chemistry, Slovak Academy of Sciences Dúbravská cesta 9, SK-845 38, Bratislava, Slovakia and [2]Department of Inorganic Chemistry, Faculty of Chemical and Food Technology, Radlinského 9, SK-812 37 Bratislava, Slovakia

Email: Monika Polákova - chemonca@savba.sk

*Corresponding author

Copies of ^{1}H and ^{13}C NMR spectra of all prepared compounds

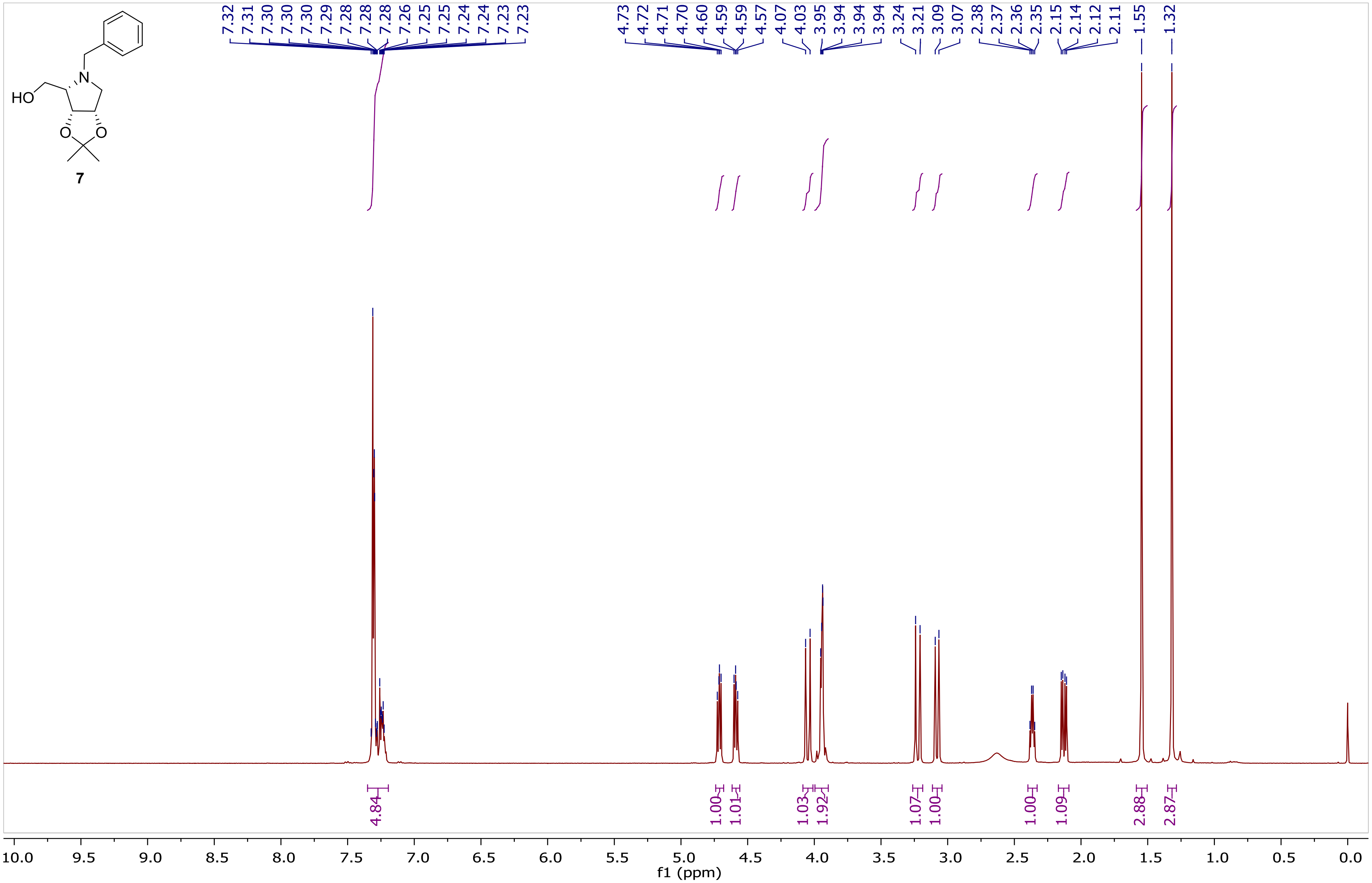
HO
N
O
O
7
7.32 7.31 7.30 7.30 7.30 7.29 7.28 7.28 7.28 7.26 7.25 7.25 7.24 7.24 7.23 7.23
4.73 4.72 4.71 4.70 4.60 4.59 4.59 4.57 4.07 4.03 3.95 3.94 3.94 3.94 3.24 3.21 3.09 3.07 2.38 2.37 2.36 2.35 2.15 2.14 2.12 2.11 1.55 1.32
4.84
1.00 1.01 1.03 1.92 1.07 1.00 1.00 1.09 2.88 2.87
10.0 9.5 9.0 8.5 8.0 7.5 7.0 6.5 6.0 5.5 5.0 4.5 4.0 3.5 3.0 2.5 2.0 1.5 1.0 0.5 0.0
f1 (ppm)

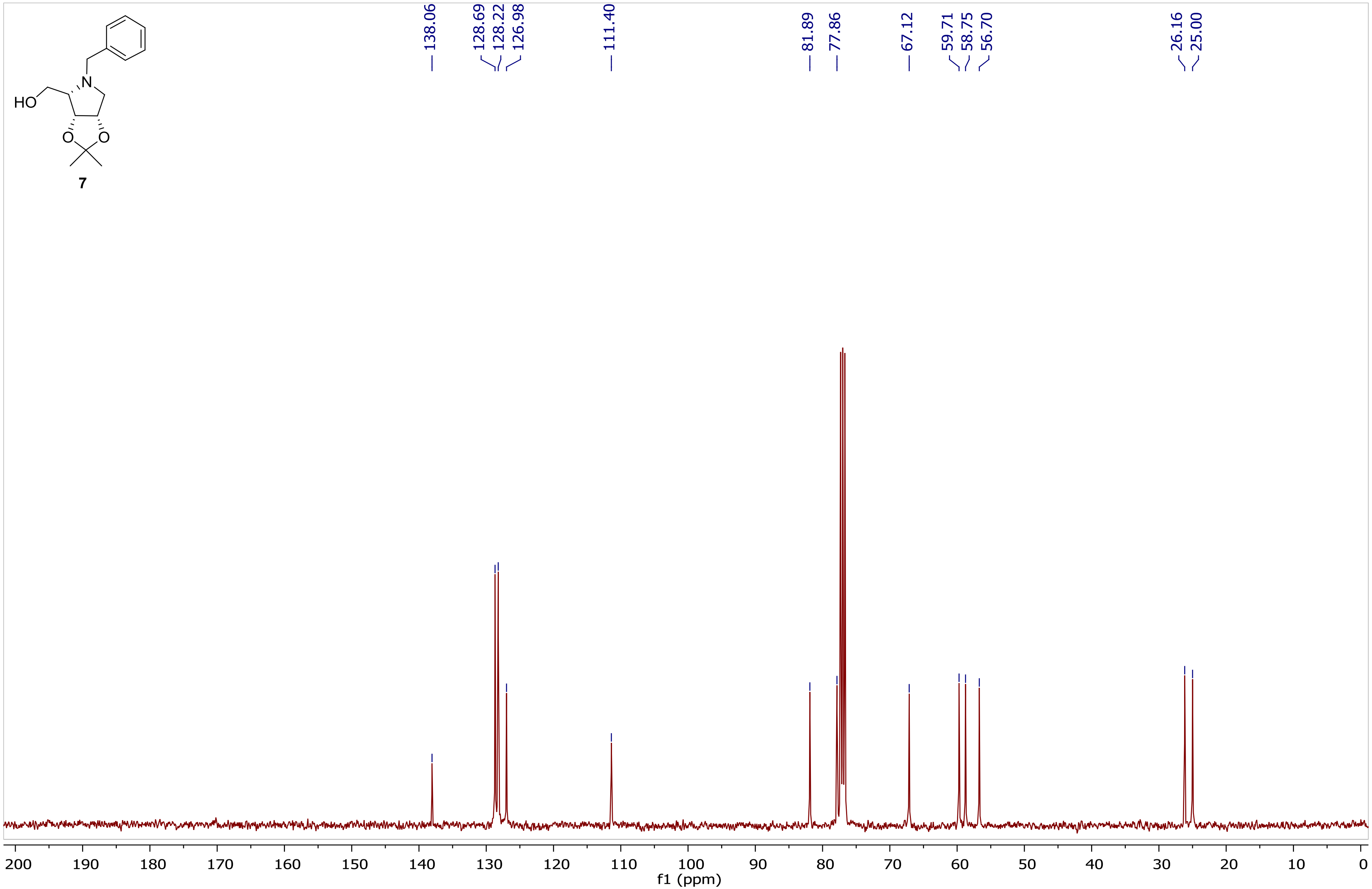

HO
N
O
O
7
138.06
128.69
128.22
126.98
111.40
81.89
77.86
67.12
59.71
58.75
56.70
26.16
25.00
200 190 180 170 160 150 140 130 120 110 100 90 80 70 60 50 40 30 20 10 0
f1 (ppm)

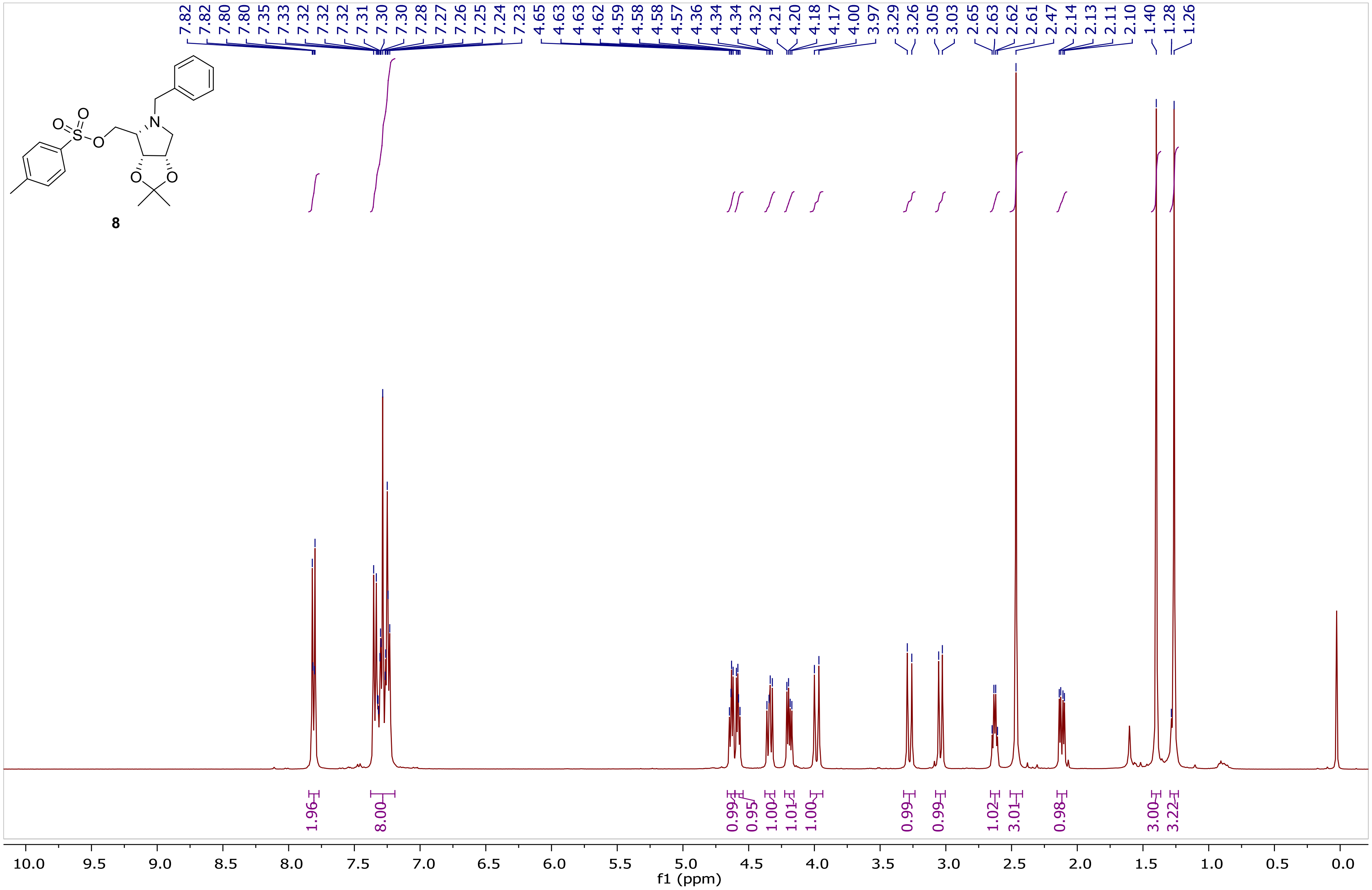
7.82 7.82 7.80 7.80 7.35 7.33 7.32 7.32 7.32 7.31 7.30 7.30 7.28 7.27 7.26 7.25 7.24 7.23 4.65 4.63 4.63 4.62 4.59 4.58 4.58 4.57 4.36 4.34 4.34 4.32 4.21 4.20 4.18 4.17 4.00 3.97 3.29 3.26 3.05 3.03 2.65 2.63 2.62 2.61 2.47 2.14 2.13 2.11 2.10 1.40 1.28 1.26
8
1.96 8.00 0.99 0.95 1.00 1.01 1.00 0.99 0.99 1.02 3.01 0.98 3.00 3.22
10.0 9.5 9.0 8.5 8.0 7.5 7.0 6.5 6.0 5.5 5.0 4.5 4.0 3.5 3.0 2.5 2.0 1.5 1.0 0.5 0.0
f1 (ppm)

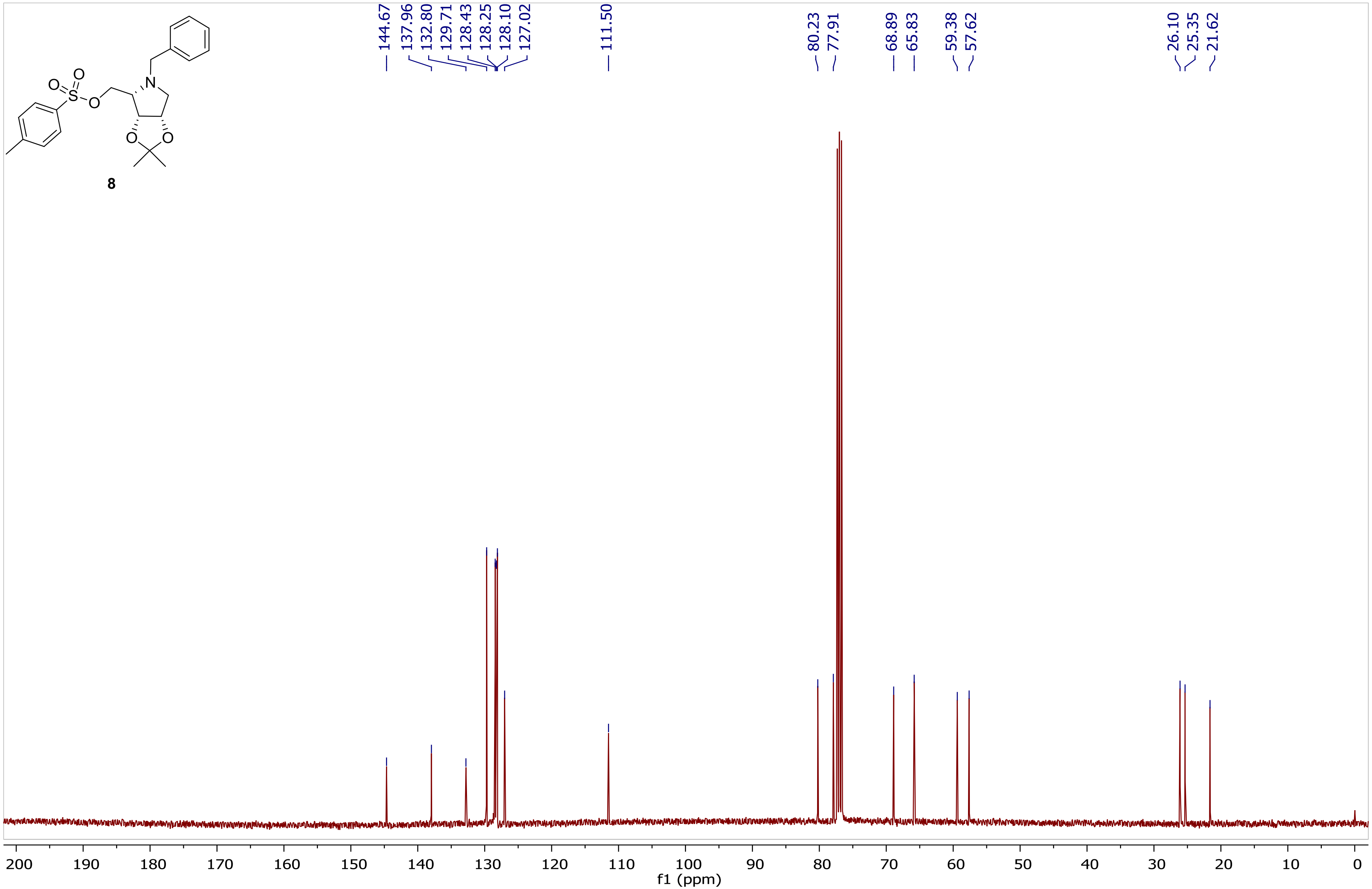
144.67
137.96
132.80
129.71
128.43
128.25
128.10
127.02
111.50
80.23
77.91
68.89
65.83
59.38
57.62
26.10
25.35
21.62
8
200 190 180 170 160 150 140 130 120 110 100 90 80 70 60 50 40 30 20 10 0
f1 (ppm)

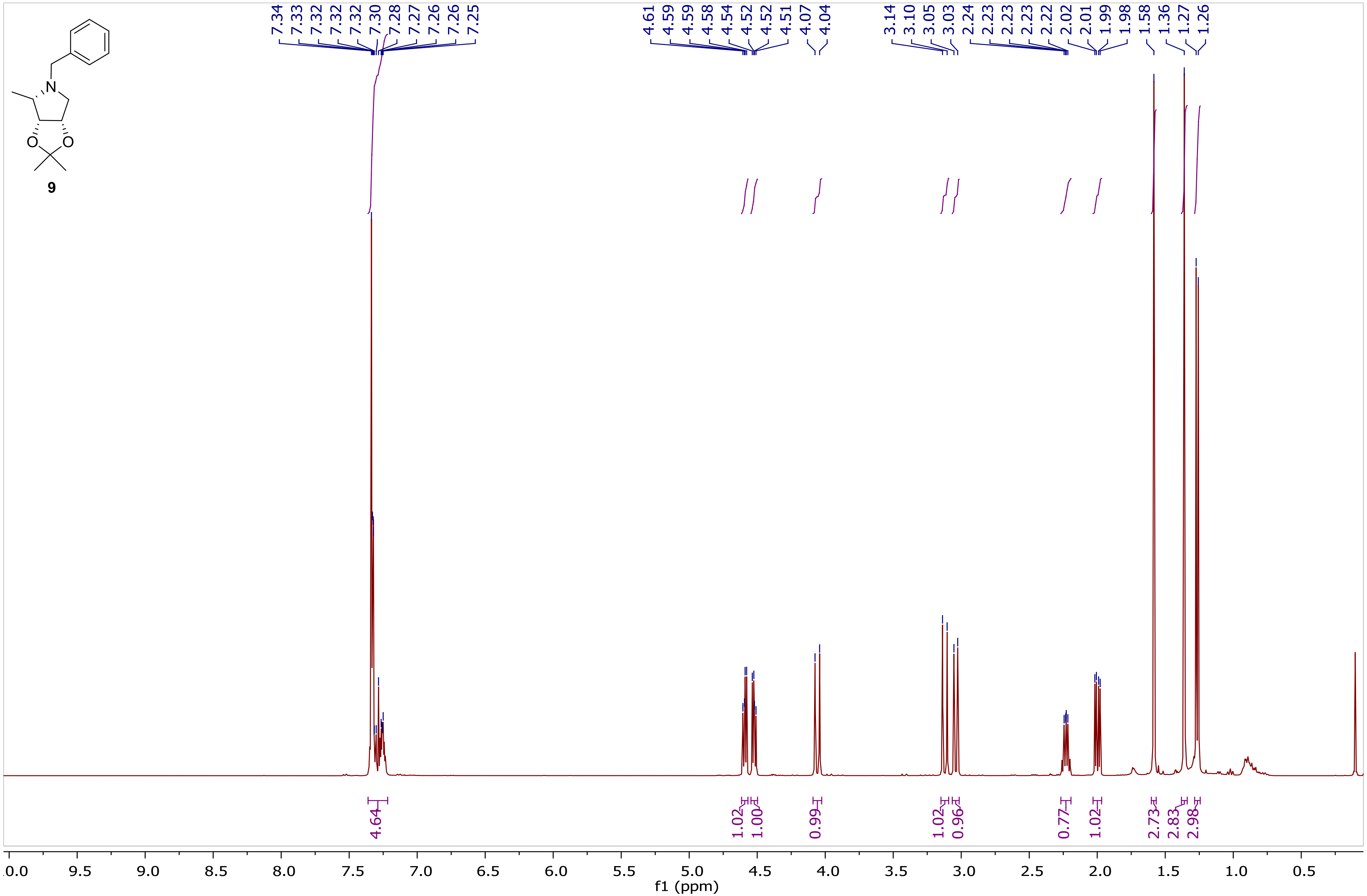
7.34 7.33 7.32 7.32 7.32 7.30 7.28 7.27 7.26 7.26 7.25
4.61 4.59 4.59 4.58 4.54 4.52 4.52 4.51 4.07 4.04
3.14 3.10 3.05 3.03 2.24 2.23 2.23 2.23 2.22 2.02 2.01 1.99 1.98 1.58 1.36 1.27 1.26
N
O
O
9
4.64
1.02 1.00
0.99
1.02 0.96
0.77
1.02
2.73 2.83 2.98
.0.0 9.5 9.0 8.5 8.0 7.5 7.0 6.5 6.0 5.5 5.0 4.5 4.0 3.5 3.0 2.5 2.0 1.5 1.0 0.5
f1 (ppm)

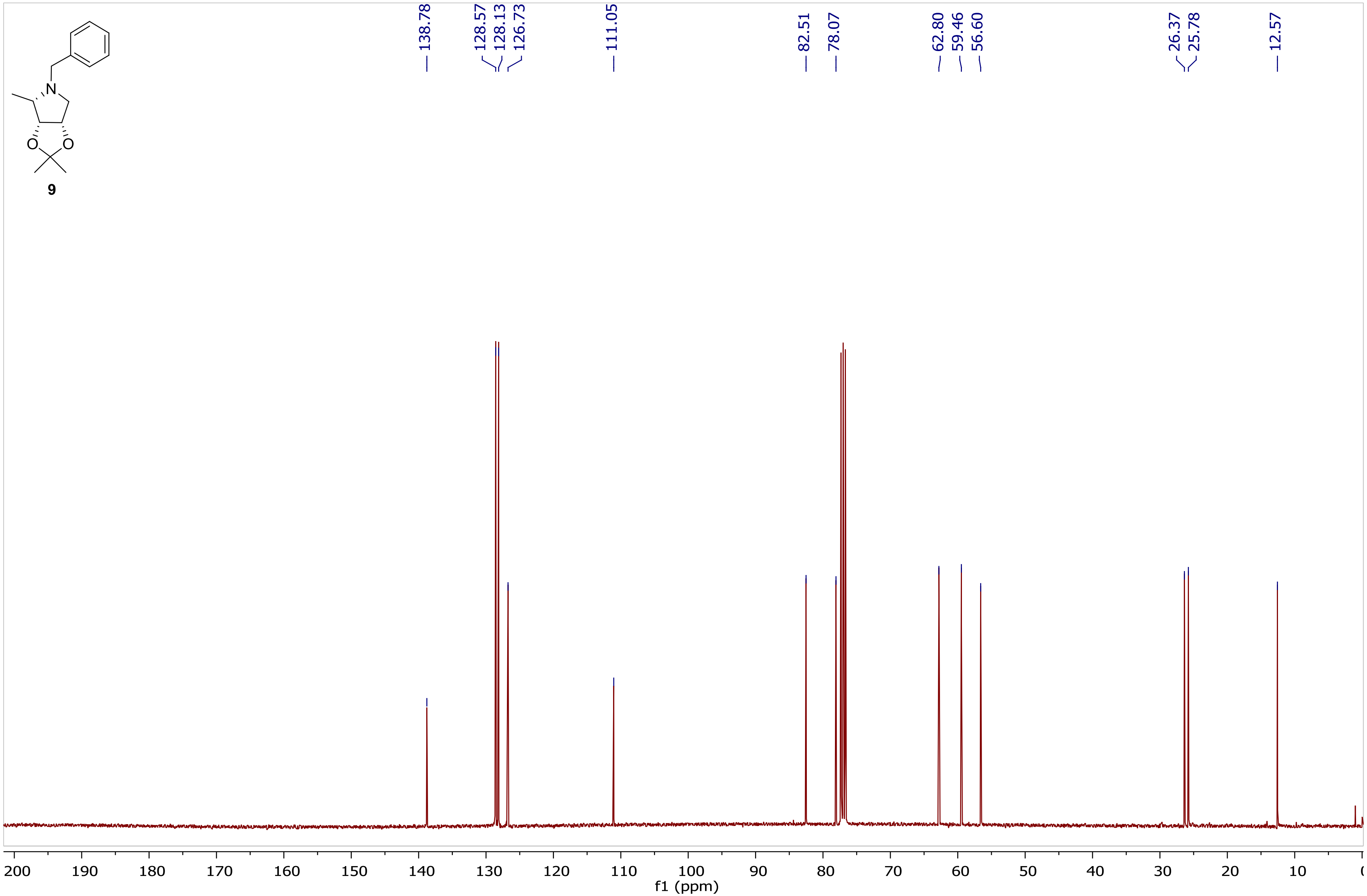
138.78
128.57
128.13
126.73
111.05
82.51
78.07
62.80
59.46
56.60
26.37
25.78
12.57
9
200 190 180 170 160 150 140 130 120 110 100 90 80 70 60 50 40 30 20 10
f1 (ppm)

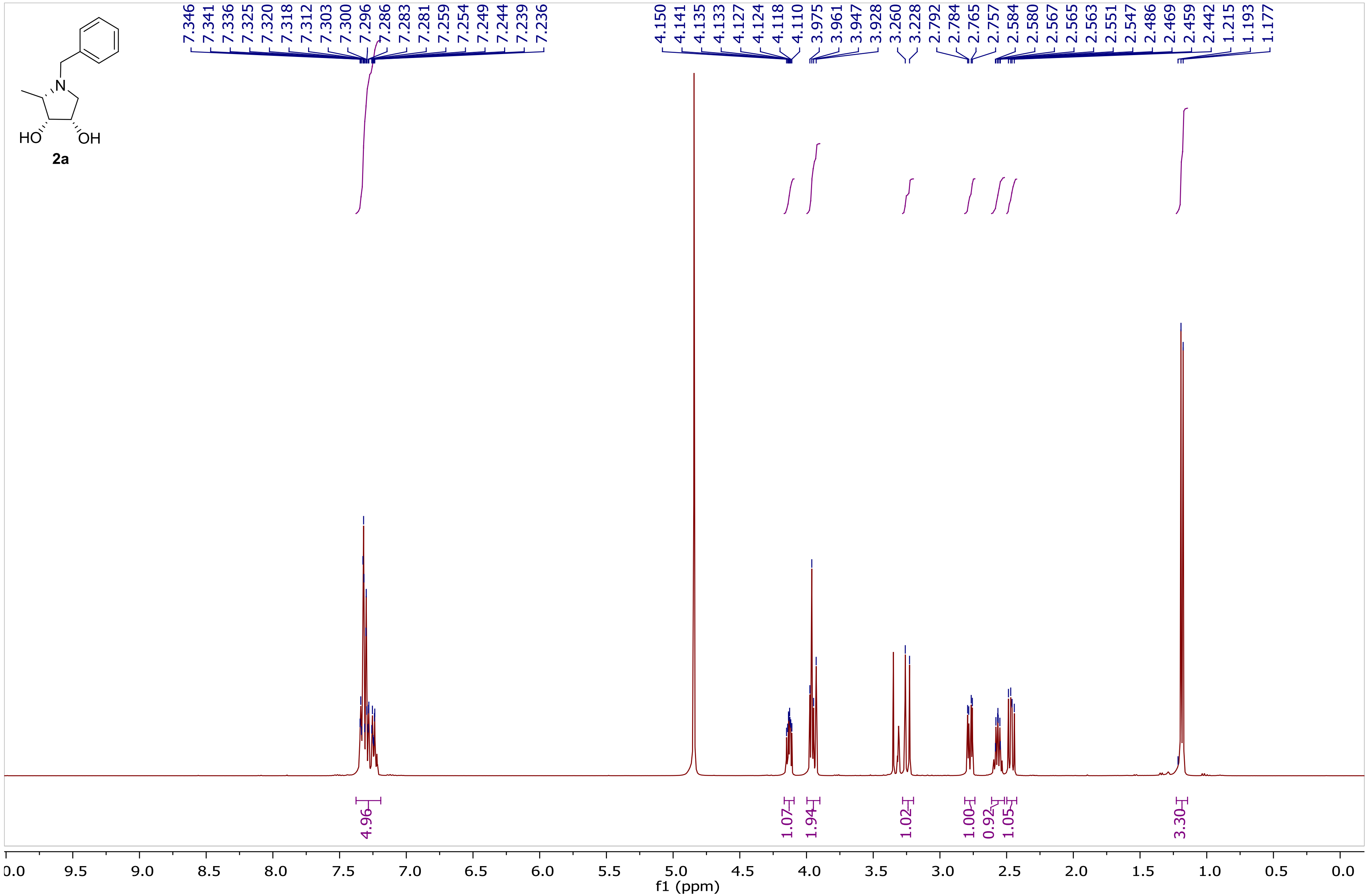
2a
7.346 7.341 7.336 7.325 7.320 7.318 7.312 7.303 7.300 7.296 7.286 7.283 7.281 7.259 7.254 7.249 7.244 7.239 7.236
4.150 4.141 4.135 4.133 4.127 4.124 4.118 4.110 3.975 3.961 3.947 3.928 3.260 3.228 2.792 2.784 2.765 2.757 2.584 2.580 2.567 2.565 2.563 2.551 2.547 2.486 2.469 2.459 2.442 1.215 1.193 1.177
4.96
1.07
1.94
1.02
1.00
0.92
1.05
3.30
10.0 9.5 9.0 8.5 8.0 7.5 7.0 6.5 6.0 5.5 5.0 4.5 4.0 3.5 3.0 2.5 2.0 1.5 1.0 0.5 0.0
f1 (ppm)

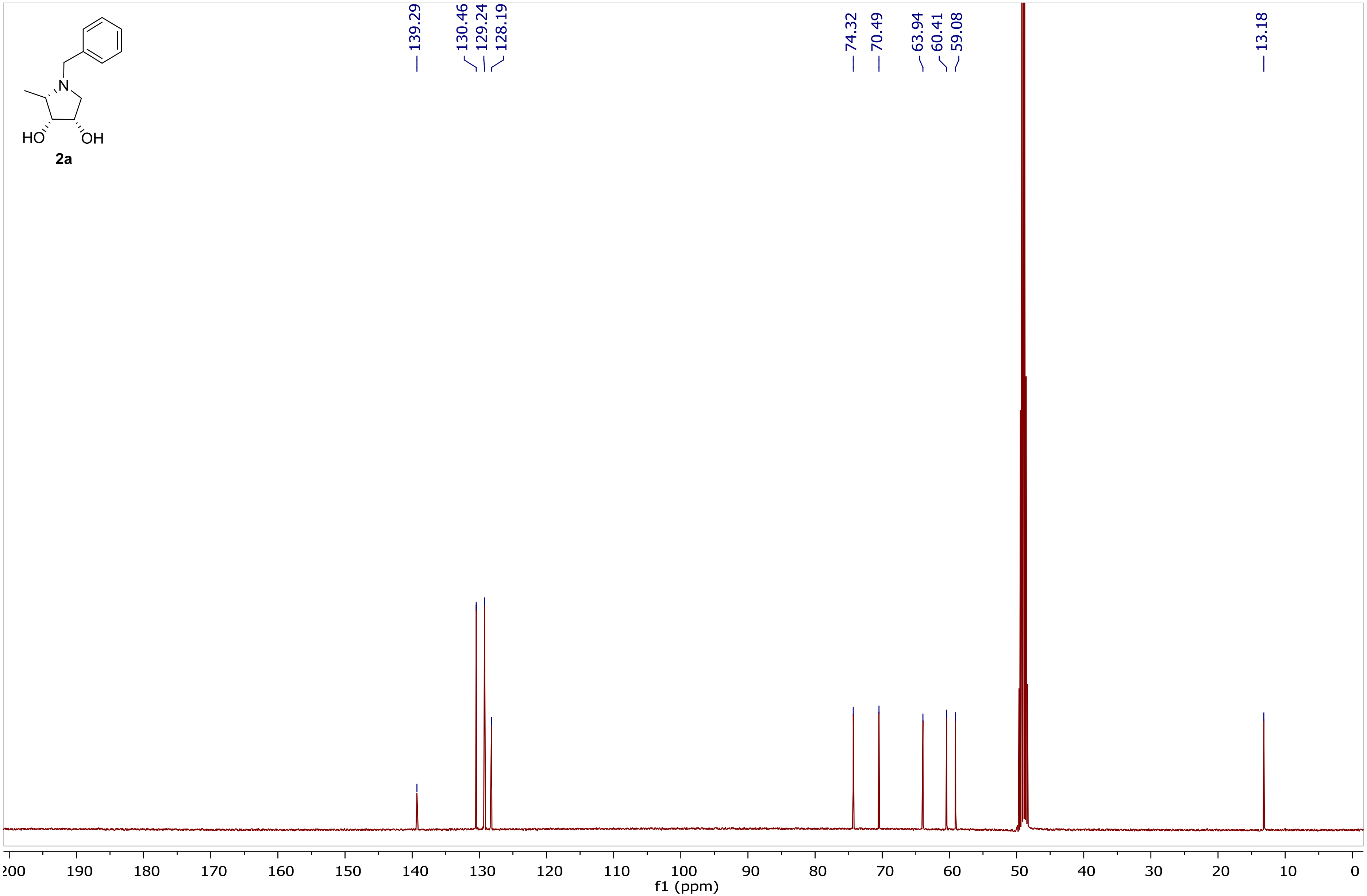
139.29
130.46
129.24
128.19
74.32
70.49
63.94
60.41
59.08
13.18
HO
OH
N
2a
200 190 180 170 160 150 140 130 120 110 100 90 80 70 60 50 40 30 20 10 0
f1 (ppm)

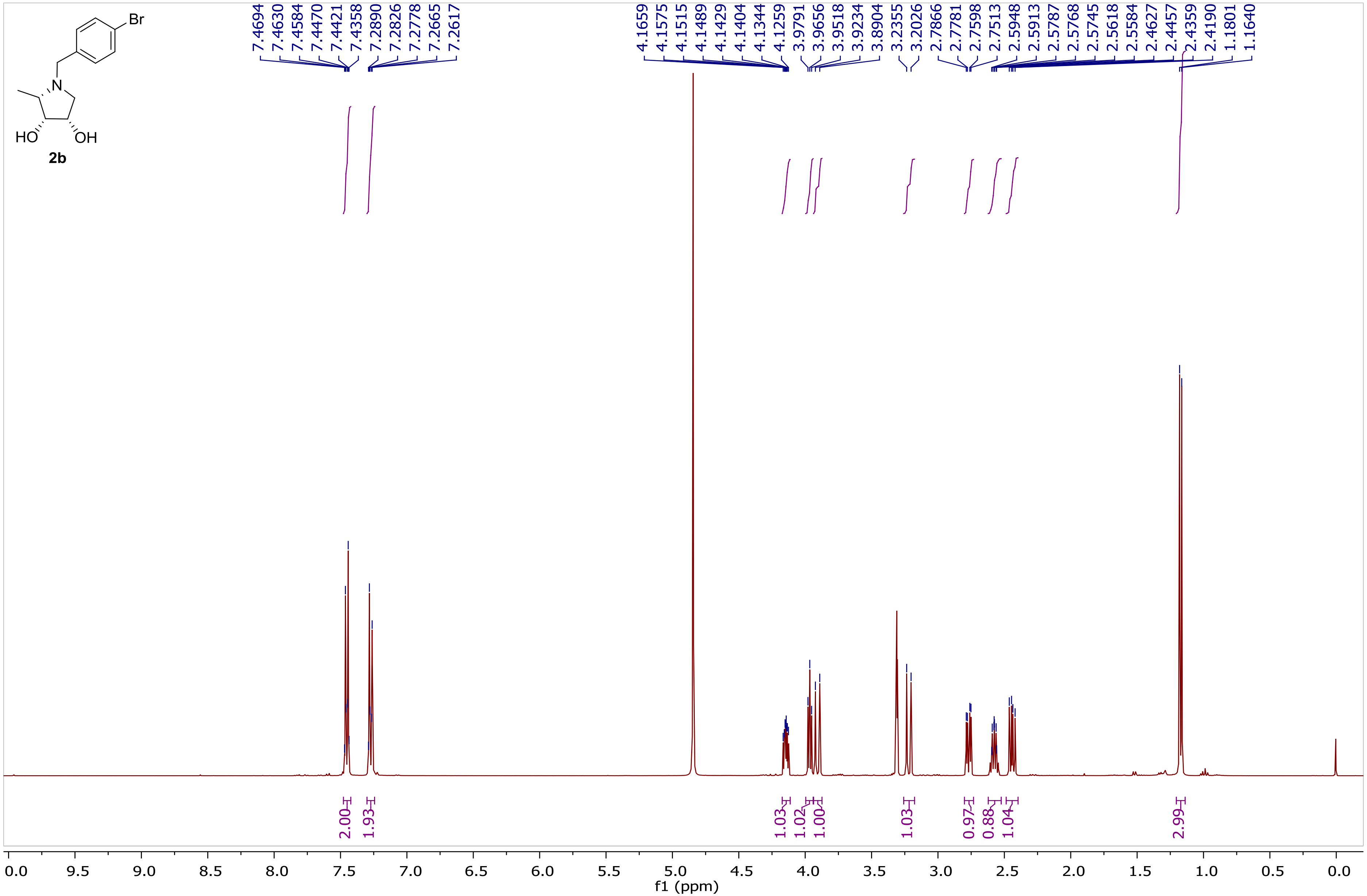
Br
N
HO
OH
2b
7.4694
7.4630
7.4584
7.4470
7.4421
7.4358
7.2890
7.2826
7.2778
7.2665
7.2617
4.1659
4.1575
4.1515
4.1489
4.1429
4.1404
4.1344
4.1259
3.9791
3.9656
3.9518
3.9234
3.8904
3.2355
3.2026
2.7866
2.7781
2.7598
2.7513
2.5948
2.5913
2.5787
2.5768
2.5745
2.5618
2.5584
2.4627
2.4457
2.4359
2.4190
1.1801
1.1640
2.00
1.93
1.03
1.02
1.00
1.03
0.97
0.88
1.04
2.99
0.0 9.5 9.0 8.5 8.0 7.5 7.0 6.5 6.0 5.5 5.0 4.5 4.0 3.5 3.0 2.5 2.0 1.5 1.0 0.5 0.0
f1 (ppm)

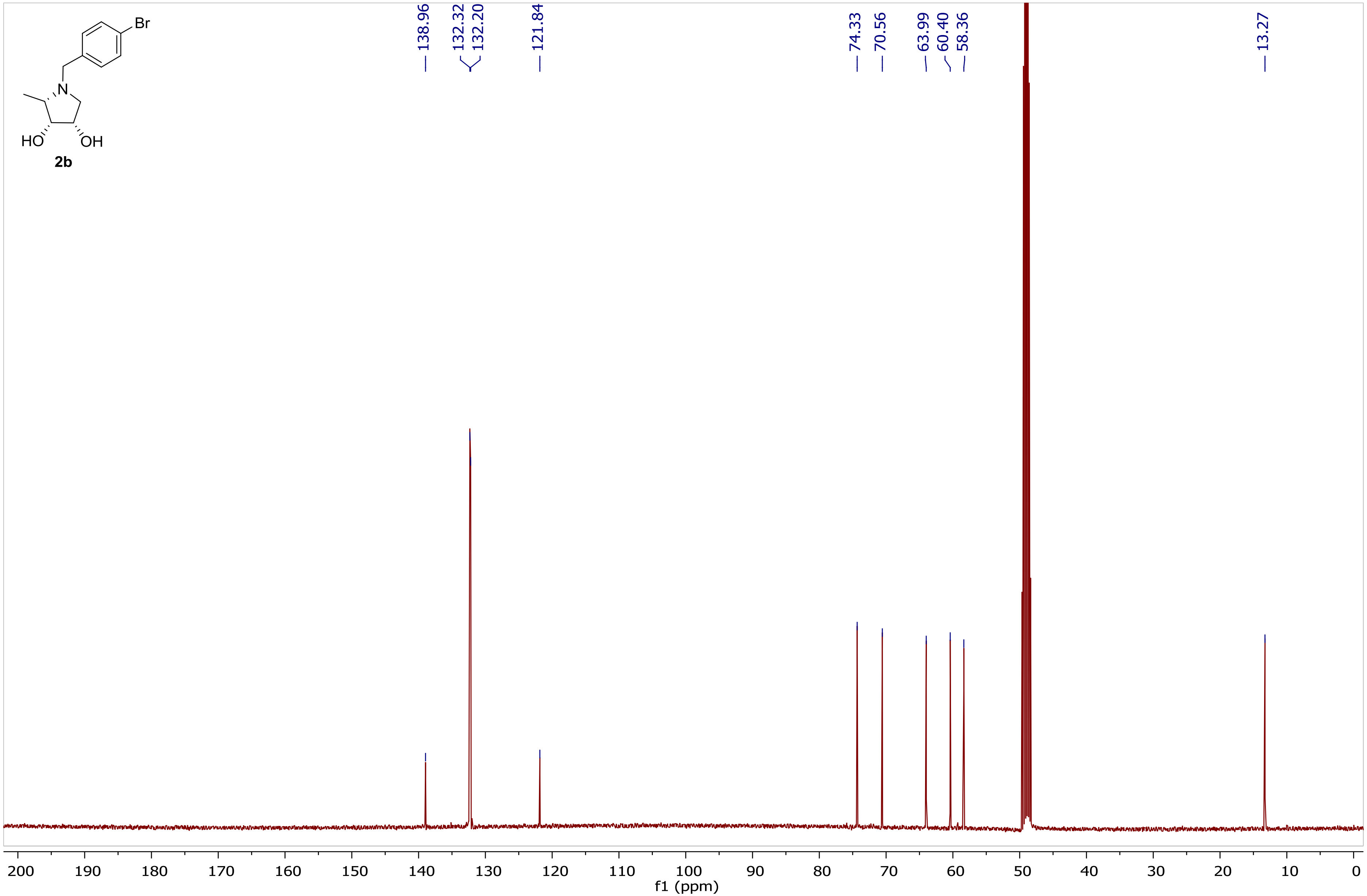
Br
N
HO
OH
2b
138.96
132.32
132.20
121.84
74.33
70.56
63.99
60.40
58.36
13.27
200 190 180 170 160 150 140 130 120 110 100 90 80 70 60 50 40 30 20 10 0
f1 (ppm)

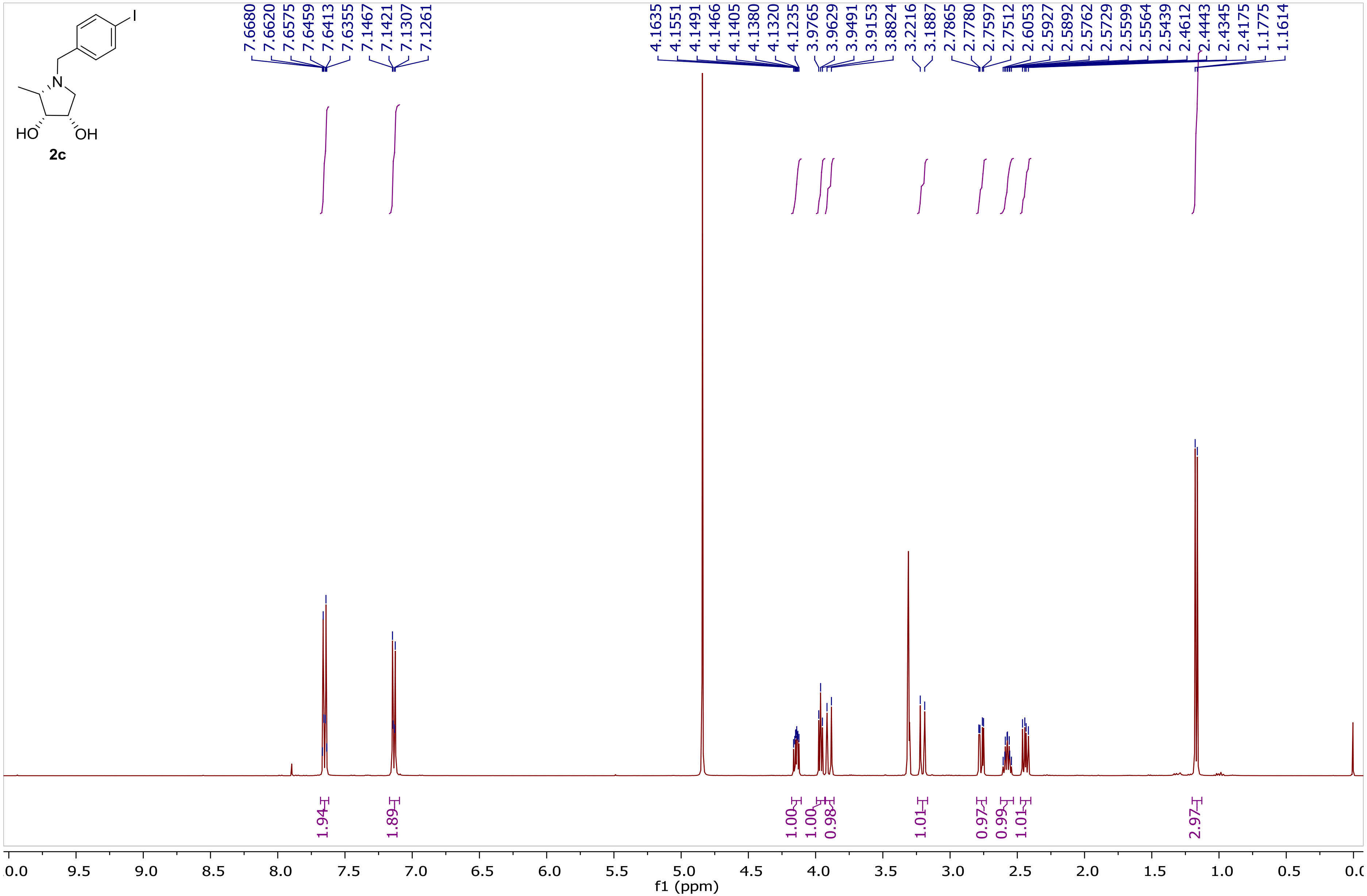
2c
7.6680
7.6620
7.6575
7.6459
7.6413
7.6355
7.1467
7.1421
7.1307
7.1261
4.1635
4.1551
4.1491
4.1466
4.1405
4.1380
4.1320
4.1235
3.9765
3.9629
3.9491
3.9153
3.8824
3.2216
3.1887
2.7865
2.7780
2.7597
2.7512
2.6053
2.5927
2.5892
2.5762
2.5729
2.5599
2.5564
2.5439
2.4612
2.4443
2.4345
2.4175
1.1775
1.1614
1.94
1.89
1.00
1.00
0.98
1.01
0.97
0.99
1.01
2.97
f1 (ppm)

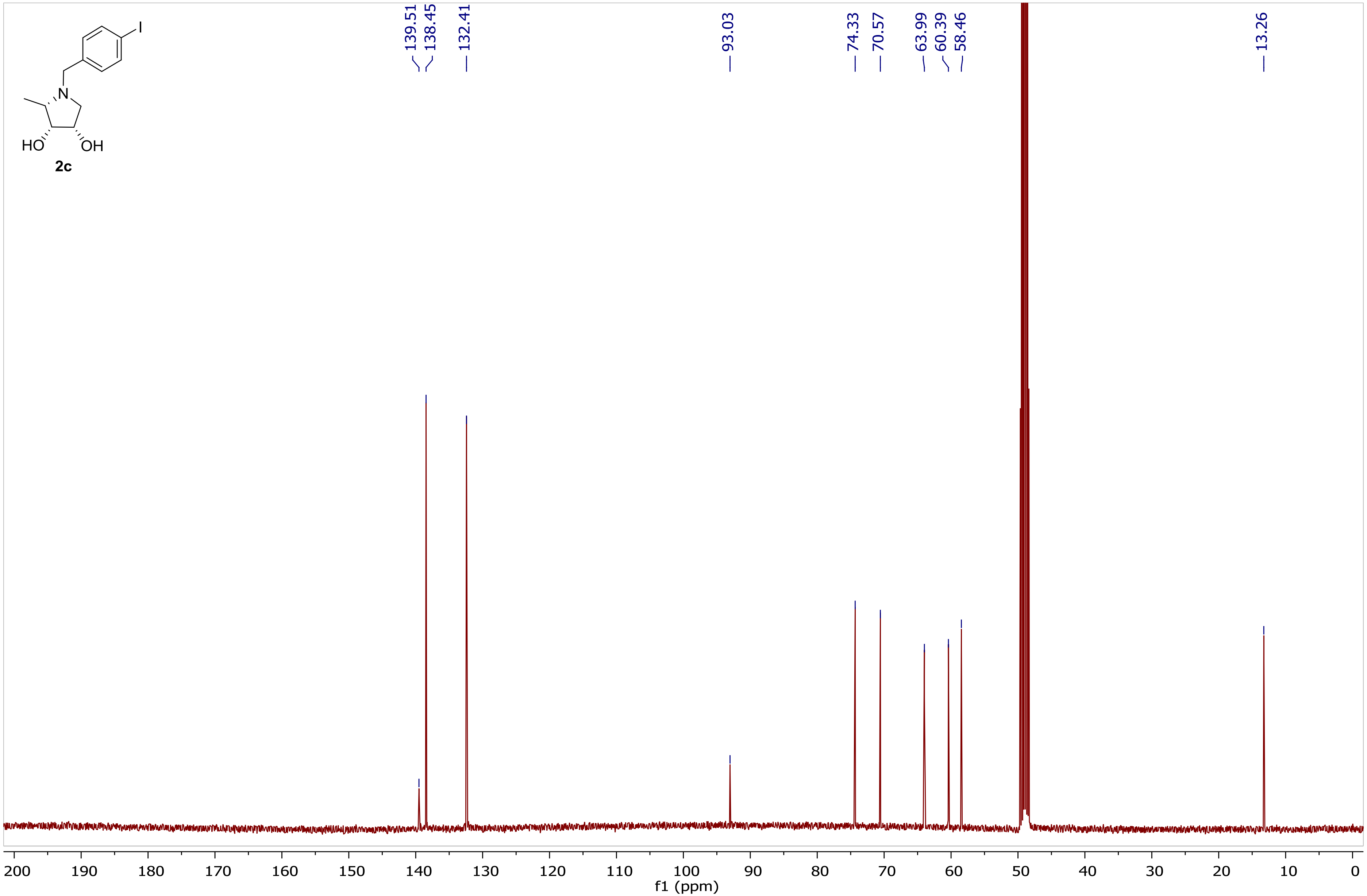

I
N
HO
OH
2c
139.51
138.45
132.41
93.03
74.33
70.57
63.99
60.39
58.46
13.26
200 190 180 170 160 150 140 130 120 110 100 90 80 70 60 50 40 30 20 10 0
f1 (ppm)

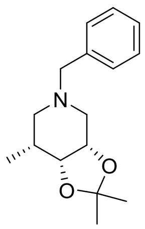
N
O
O

11

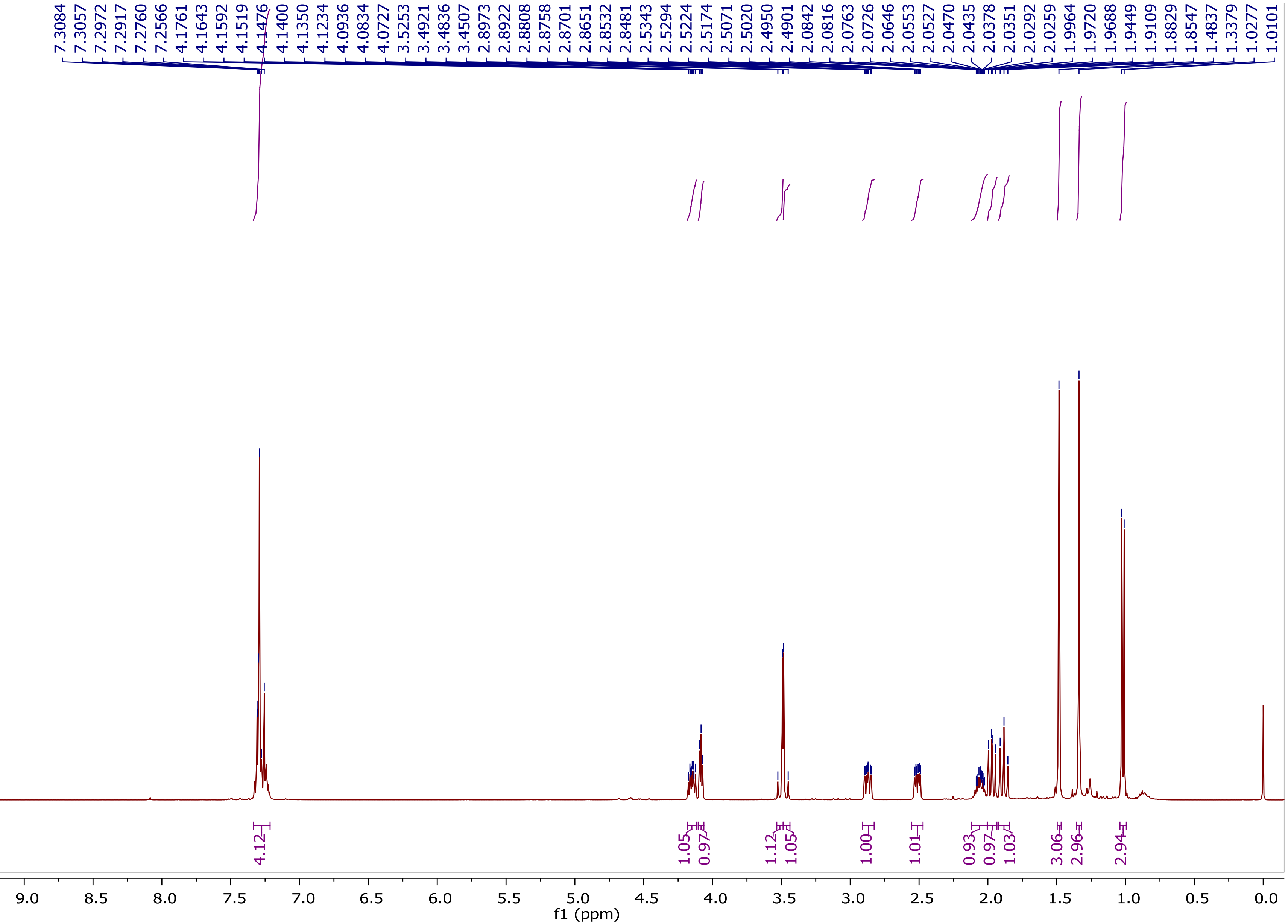
7.3084
7.3057
7.2972
7.2917
7.2760
7.2566
4.1761
4.1643
4.1592
4.1519
4.1476
4.1400
4.1350
4.1234
4.0936
4.0834
4.0727
3.5253
3.4921
3.4836
3.4507
2.8973
2.8922
2.8808
2.8758
2.8701
2.8651
2.8532
2.8481
2.5343
2.5294
2.5224
2.5174
2.5071
2.5020
2.4950
2.4901
2.0842
2.0816
2.0763
2.0726
2.0646
2.0553
2.0527
2.0470
2.0435
2.0378
2.0351
2.0292
2.0259
1.9964
1.9720
1.9688
1.9449
1.9109
1.8829
1.8547
1.4837
1.3379
1.0277
1.0101
4.12
1.05
0.97
1.12
1.05
1.00
1.01
0.93
0.97
1.03
3.06
2.96
2.94
0.0
9.5
9.0
8.5
8.0
7.5
7.0
6.5
6.0
5.5
5.0
4.5
4.0
3.5
3.0
2.5
2.0
1.5
1.0
0.5
0.0
f1 (ppm)

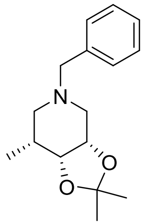

11

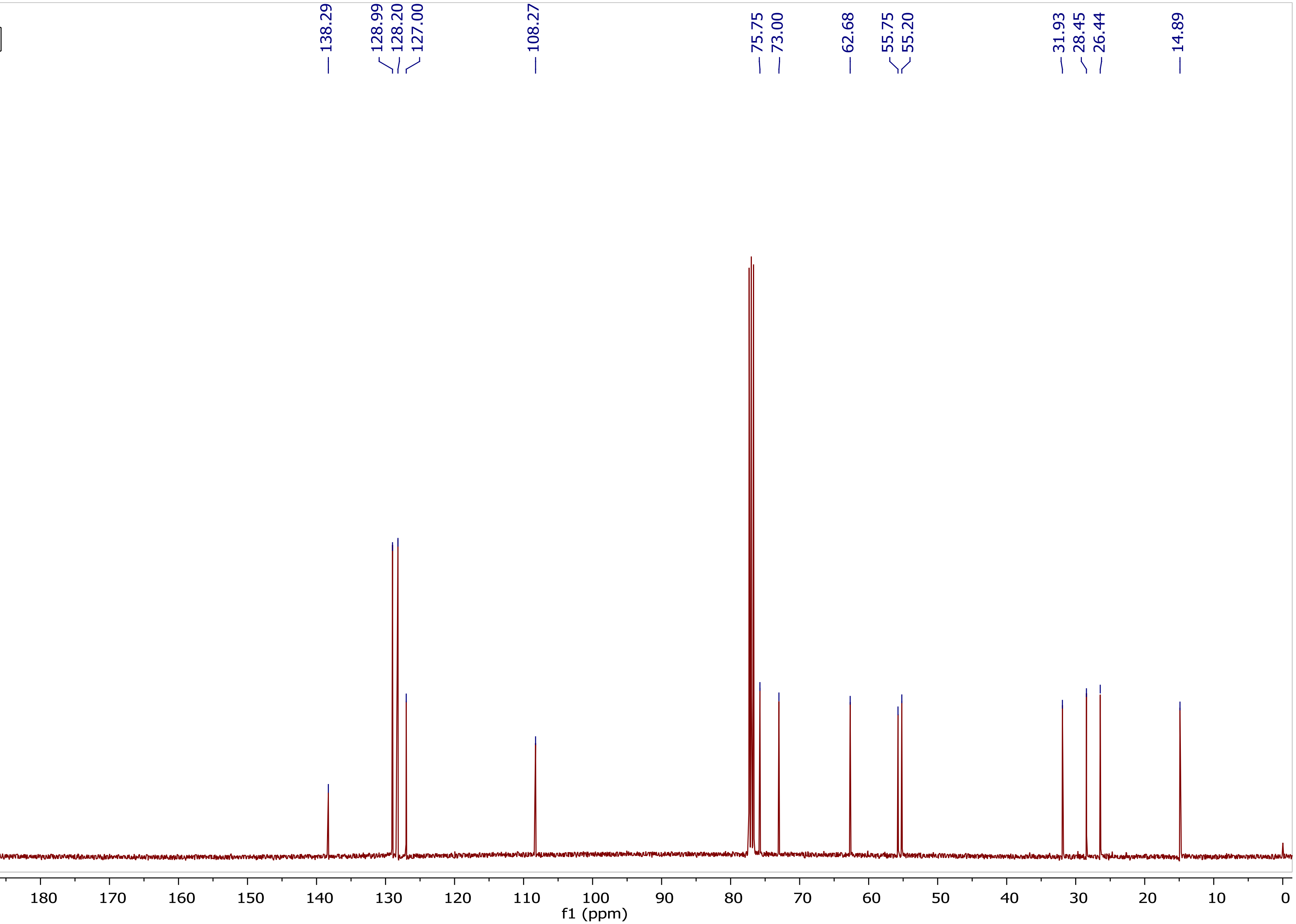
138.29
128.99
128.20
127.00
108.27
75.75
73.00
62.68
55.75
55.20
31.93
28.45
26.44
14.89
200 190 180 170 160 150 140 130 120 110 100 90 80 70 60 50 40 30 20 10 0
f1 (ppm)

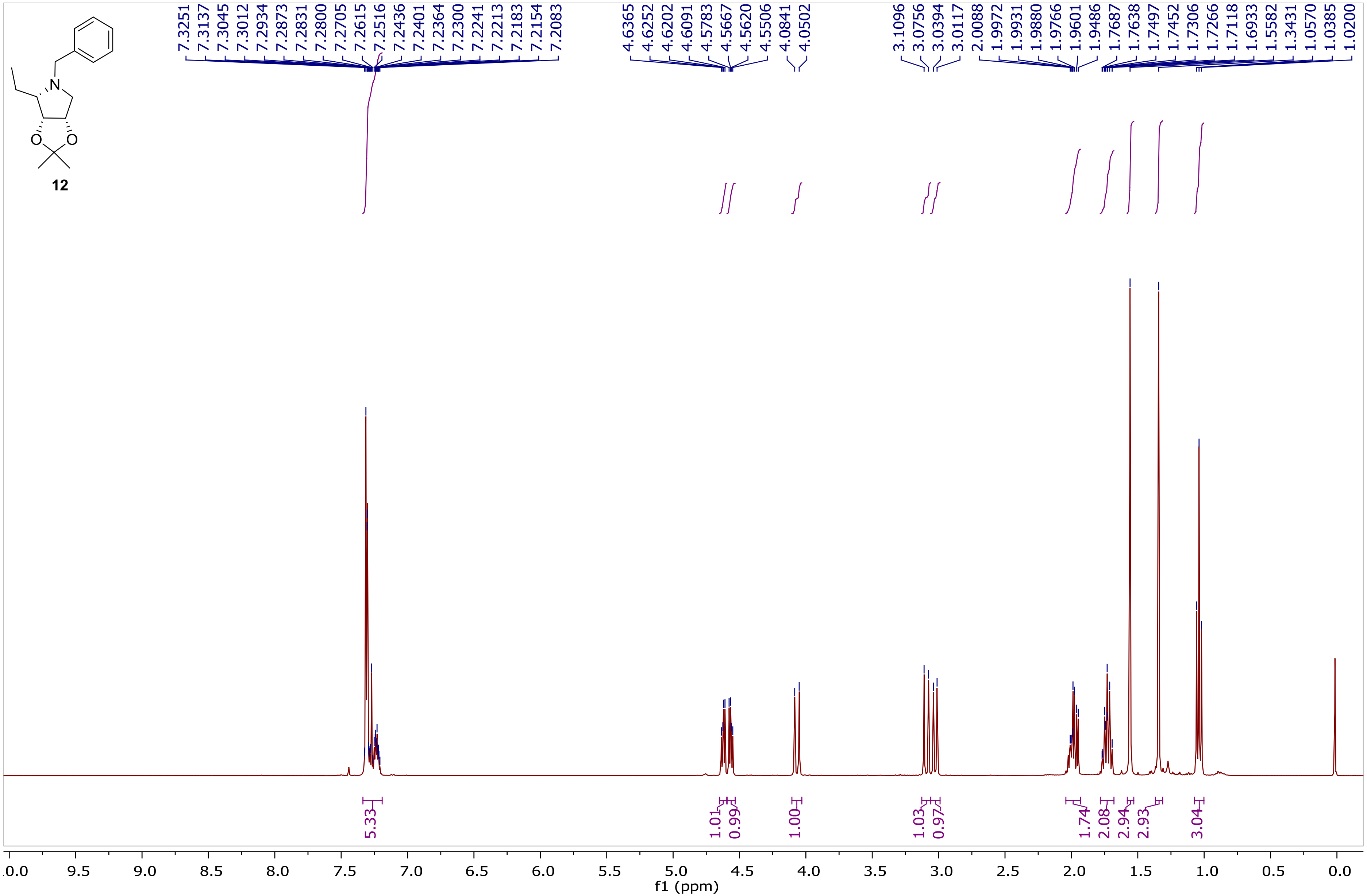
N
O
O
12
7.3251
7.3137
7.3045
7.3012
7.2934
7.2873
7.2831
7.2800
7.2705
7.2615
7.2516
7.2436
7.2401
7.2364
7.2300
7.2241
7.2213
7.2183
7.2154
7.2083
4.6365
4.6252
4.6202
4.6091
4.5783
4.5667
4.5620
4.5506
4.0841
4.0502
3.1096
3.0756
3.0394
3.0117
2.0088
1.9972
1.9931
1.9880
1.9766
1.9601
1.9486
1.7687
1.7638
1.7497
1.7452
1.7306
1.7266
1.7118
1.6933
1.5582
1.3431
1.0570
1.0385
1.0200
5.33
1.01
0.99
1.00
1.03
0.97
1.74
2.08
2.94
2.93
3.04
f1 (ppm)

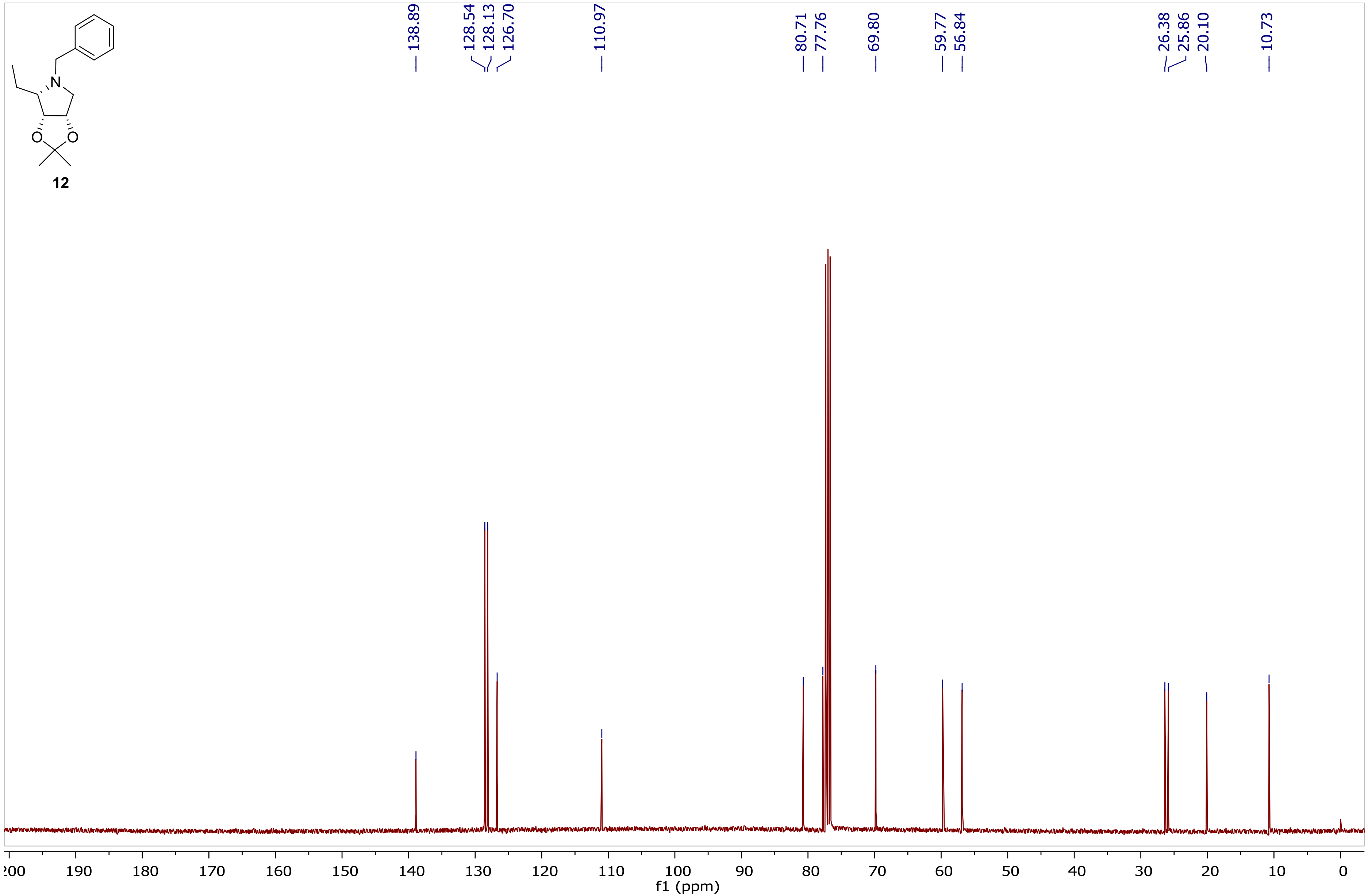
138.89
128.54
128.13
126.70
110.97
80.71
77.76
69.80
59.77
56.84
26.38
25.86
20.10
10.73
N
O
O
12
200 190 180 170 160 150 140 130 120 110 100 90 80 70 60 50 40 30 20 10 0
f1 (ppm)

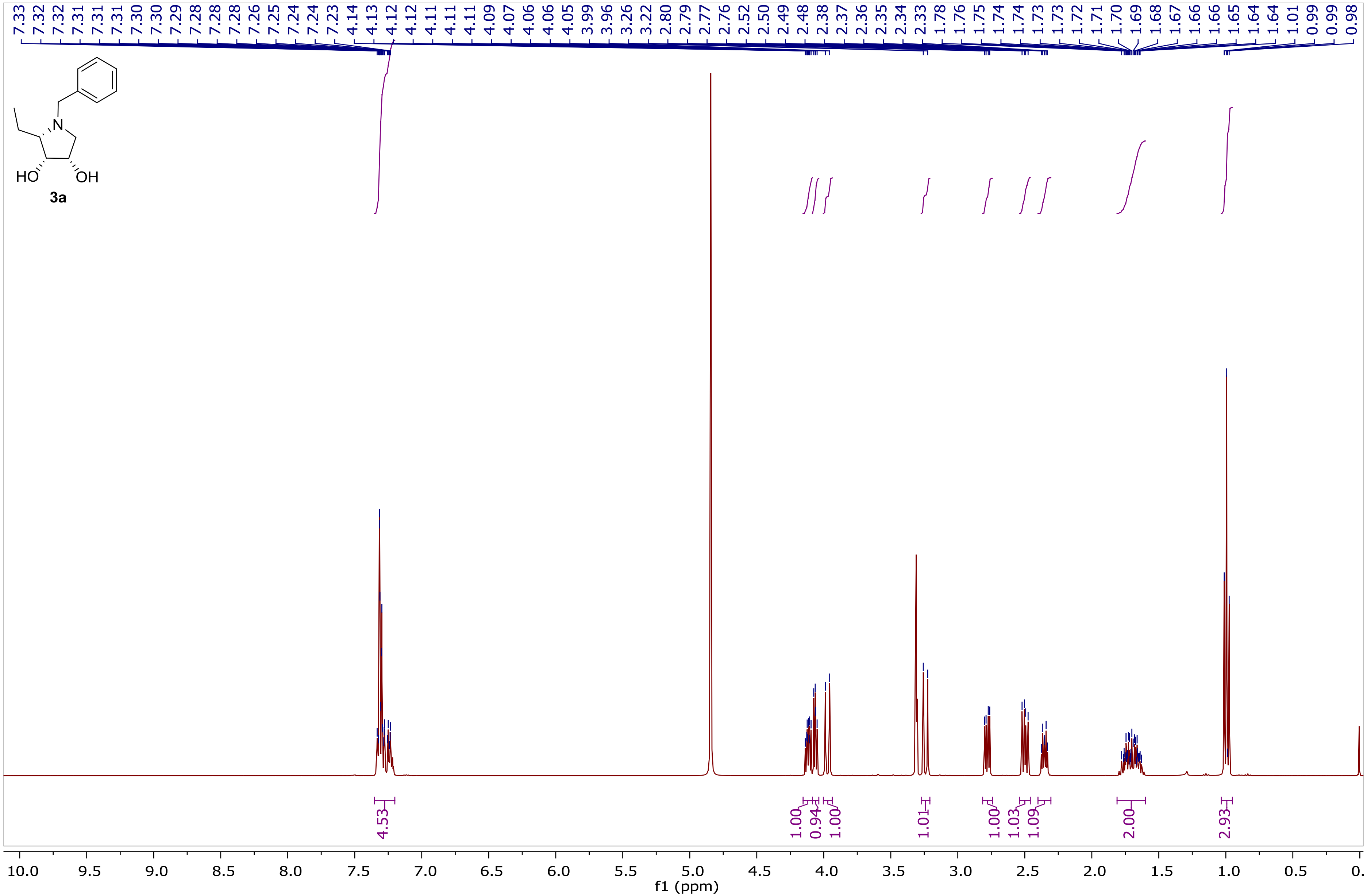
HO
OH
N
3a
f1 (ppm)

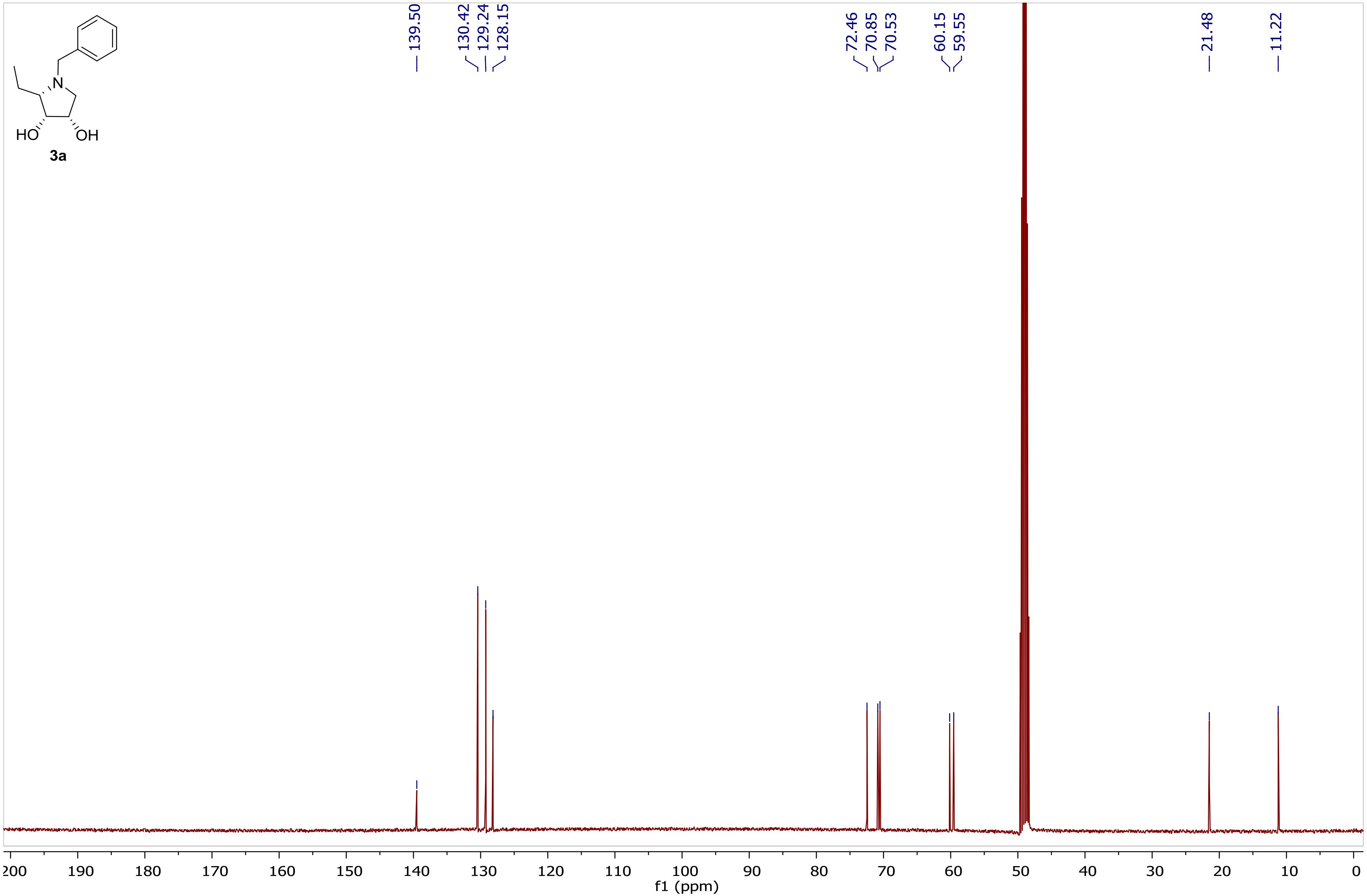
3a
HO
OH
N
139.50
130.42
129.24
128.15
72.46
70.85
70.53
60.15
59.55
21.48
11.22
200 190 180 170 160 150 140 130 120 110 100 90 80 70 60 50 40 30 20 10 0
f1 (ppm)

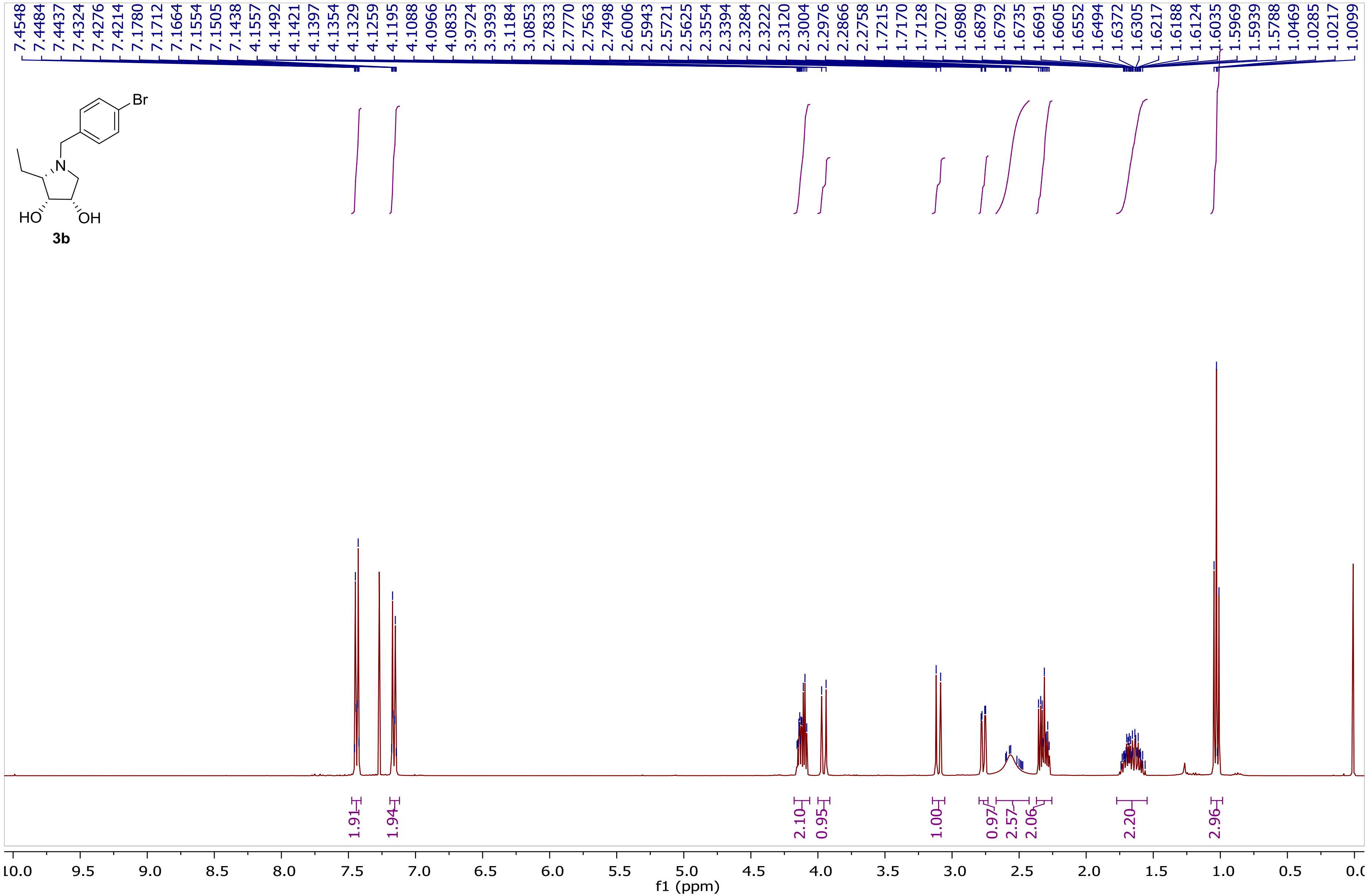

7.4548
7.4484
7.4437
7.4324
7.4276
7.4214
7.1780
7.1712
7.1664
7.1554
7.1505
7.1438
4.1557
4.1492
4.1421
4.1397
4.1354
4.1329
4.1259
4.1195
4.1088
4.0966
4.0835
3.9724
3.9393
3.1184
3.0853
2.7833
2.7770
2.7563
2.7498
2.6006
2.5943
2.5721
2.5625
2.3554
2.3394
2.3284
2.3222
2.3120
2.3004
2.2976
2.2866
2.2758
1.7215
1.7170
1.7128
1.7027
1.6980
1.6879
1.6792
1.6735
1.6691
1.6605
1.6552
1.6494
1.6372
1.6305
1.6217
1.6188
1.6124
1.6035
1.5969
1.5939
1.5788
1.0469
1.0285
1.0217
1.0099
Br
N
HO
OH
3b
1.91
1.94
2.10
0.95
1.00
0.97
2.57
2.06
2.20
2.96
f1 (ppm)

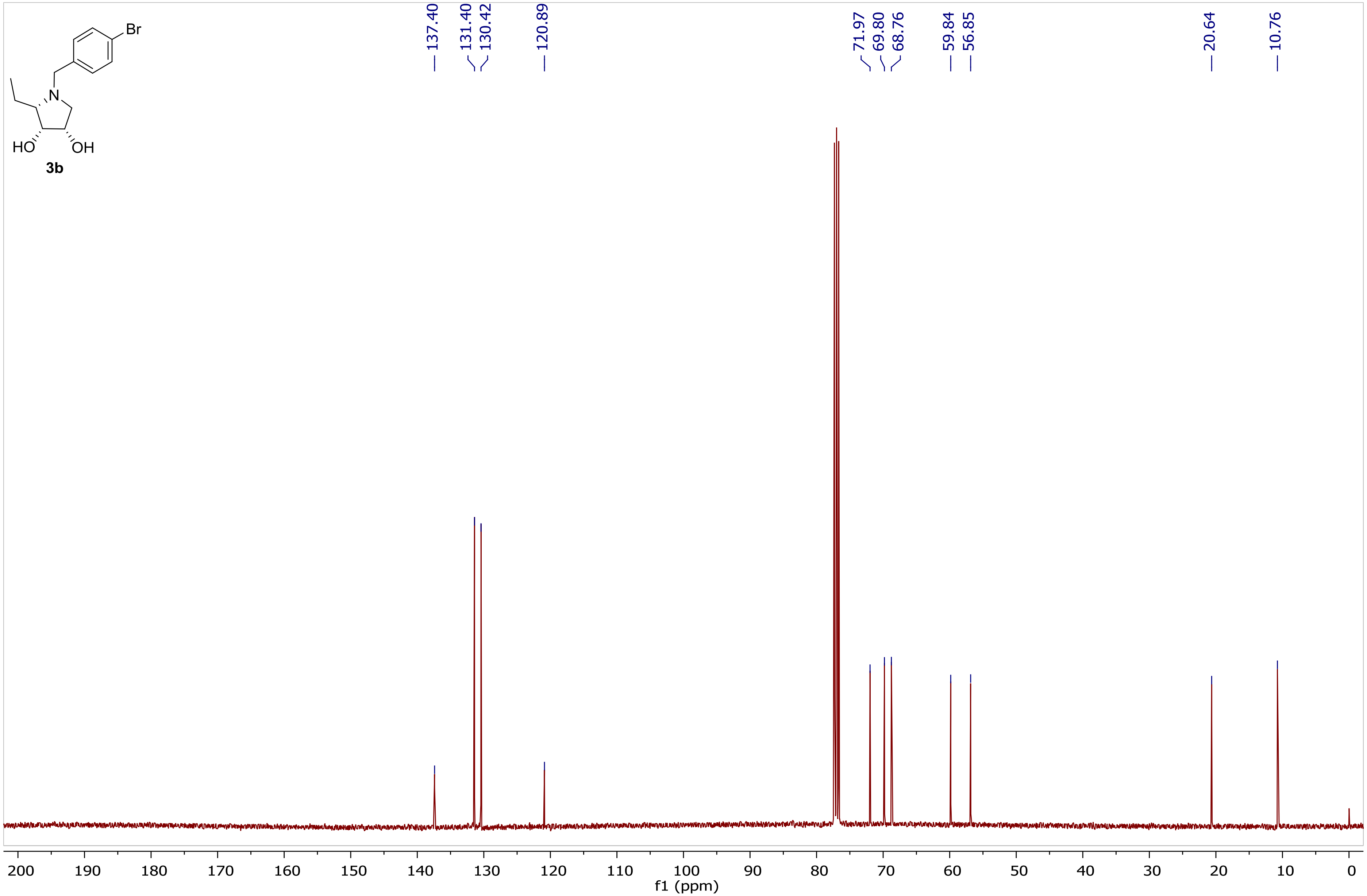
Br
N
HO
OH
3b
137.40
131.40
130.42
120.89
71.97
69.80
68.76
59.84
56.85
20.64
10.76
200 190 180 170 160 150 140 130 120 110 100 90 80 70 60 50 40 30 20 10 0
f1 (ppm)

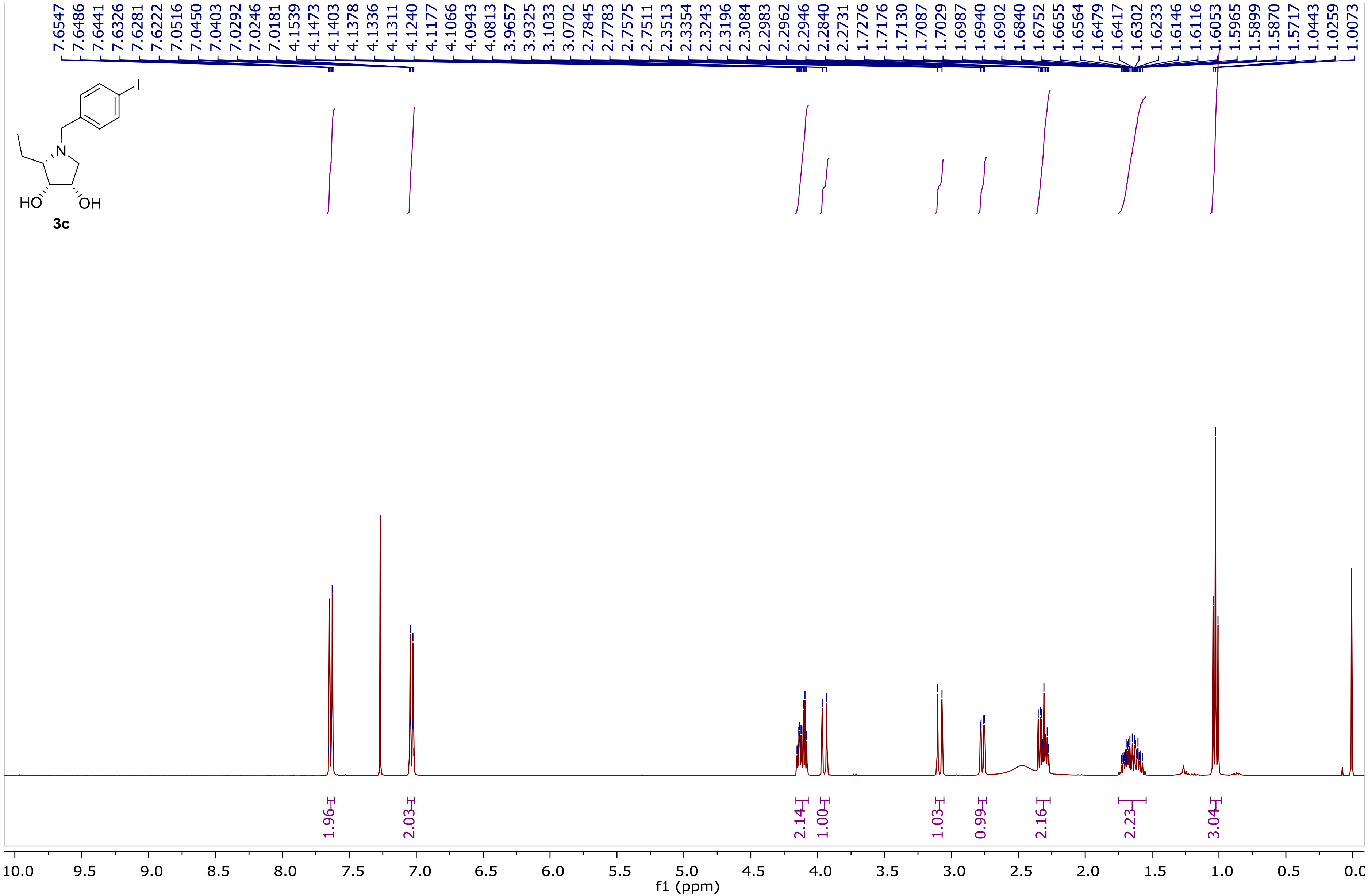
7.6547 7.6486 7.6441 7.6326 7.6281 7.6222 7.0516 7.0450 7.0403 7.0292 7.0246 7.0181 4.1539 4.1473 4.1403 4.1378 4.1336 4.1311 4.1240 4.1177 4.1066 4.0943 4.0813 3.9657 3.9325 3.1033 3.0702 2.7845 2.7783 2.7575 2.7511 2.3513 2.3354 2.3243 2.3196 2.3084 2.2983 2.2962 2.2946 2.2840 2.2731 1.7276 1.7176 1.7130 1.7087 1.7029 1.6987 1.6940 1.6902 1.6840 1.6752 1.6655 1.6564 1.6479 1.6417 1.6302 1.6233 1.6146 1.6116 1.6053 1.5965 1.5899 1.5870 1.5717 1.0443 1.0259 1.0073
I
N
HO
OH
3c
1.96
2.03
2.14
1.00
1.03
0.99
2.16
2.23
3.04
10.0 9.5 9.0 8.5 8.0 7.5 7.0 6.5 6.0 5.5 5.0 4.5 4.0 3.5 3.0 2.5 2.0 1.5 1.0 0.5 0.0
f1 (ppm)

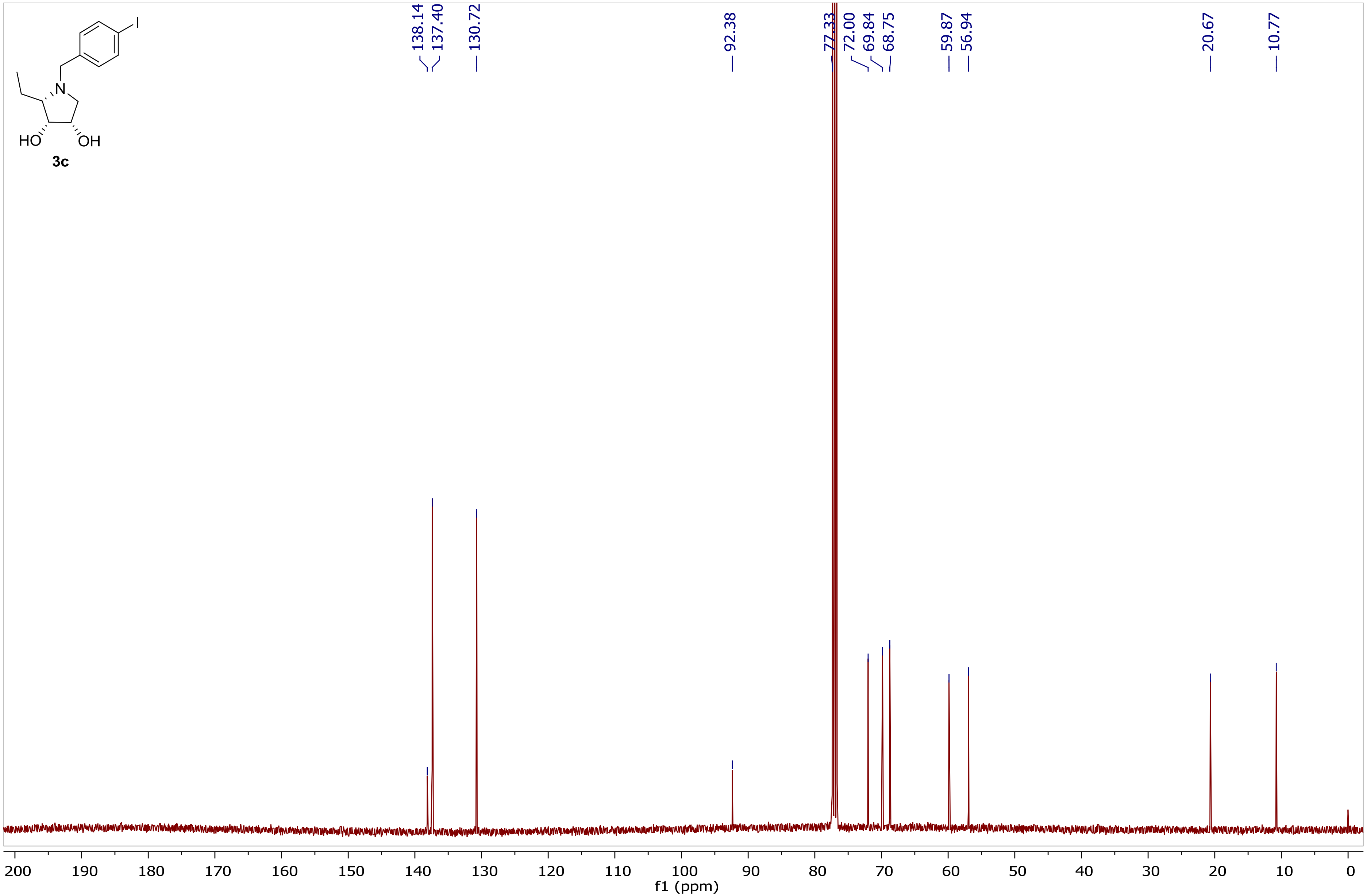

HO
OH
N
I
3c
138.14
137.40
130.72
92.38
77.33
72.00
69.84
68.75
59.87
56.94
20.67
10.77
200 190 180 170 160 150 140 130 120 110 100 90 80 70 60 50 40 30 20 10 0
f1 (ppm)

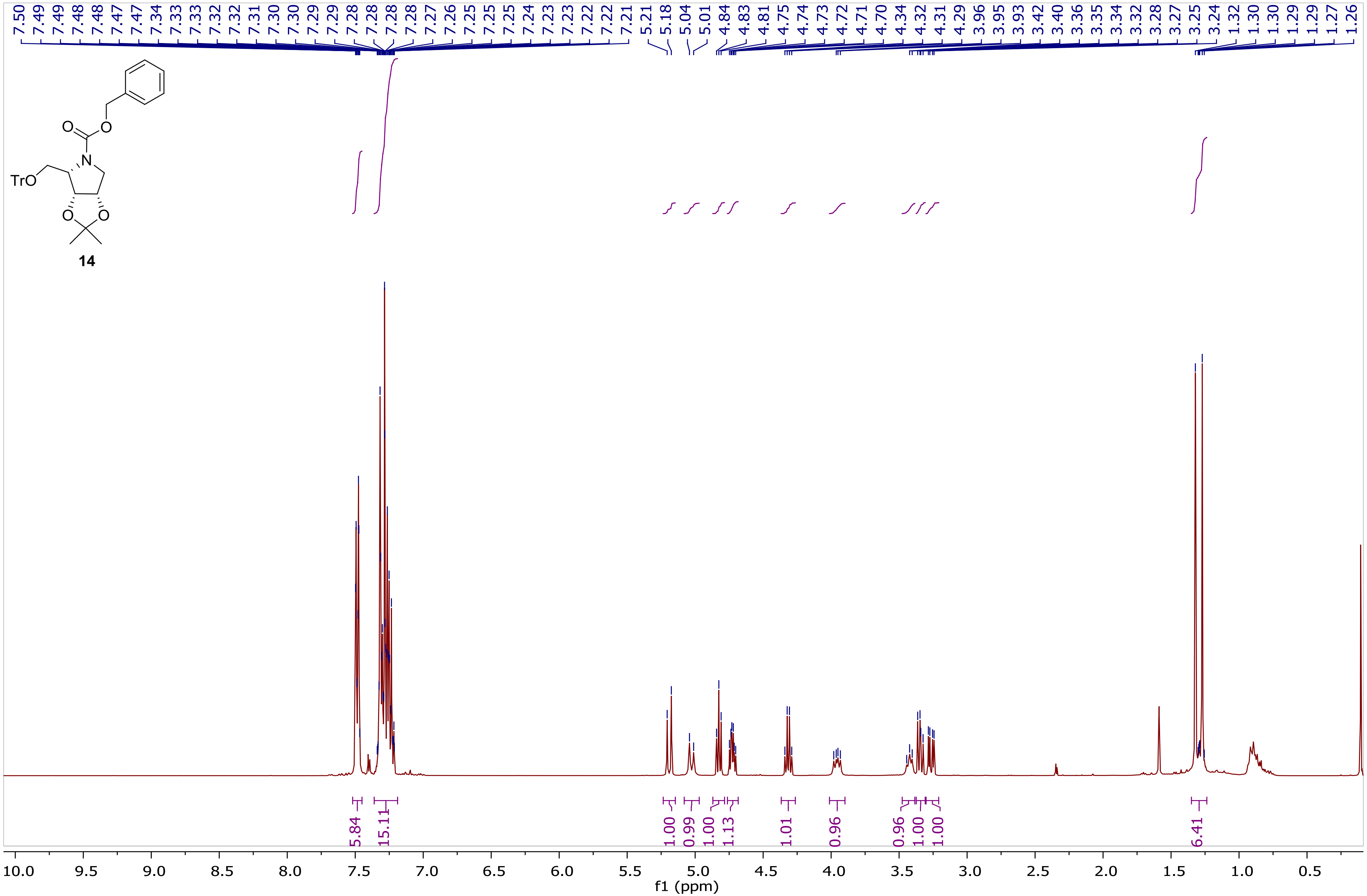

7.50
7.49
7.49
7.48
7.48
7.47
7.47
7.34
7.33
7.33
7.32
7.32
7.31
7.30
7.30
7.29
7.29
7.28
7.28
7.28
7.28
7.27
7.26
7.25
7.25
7.25
7.24
7.23
7.23
7.22
7.22
7.21
5.21
5.18
5.04
5.01
4.84
4.83
4.81
4.75
4.74
4.73
4.72
4.71
4.70
4.34
4.32
4.31
4.29
3.96
3.95
3.93
3.42
3.40
3.36
3.35
3.34
3.32
3.28
3.27
3.25
3.24
1.32
1.30
1.30
1.29
1.29
1.27
1.26
TrO
O
O
N
O
O
5.84
15.11
1.00
0.99
1.00
1.13
1.01
0.96
0.96
1.00
1.00
6.41
10.0
9.5
9.0
8.5
8.0
7.5
7.0
6.5
6.0
5.5
5.0
4.5
4.0
3.5
3.0
2.5
2.0
1.5
1.0
0.5
f1 (ppm)

14

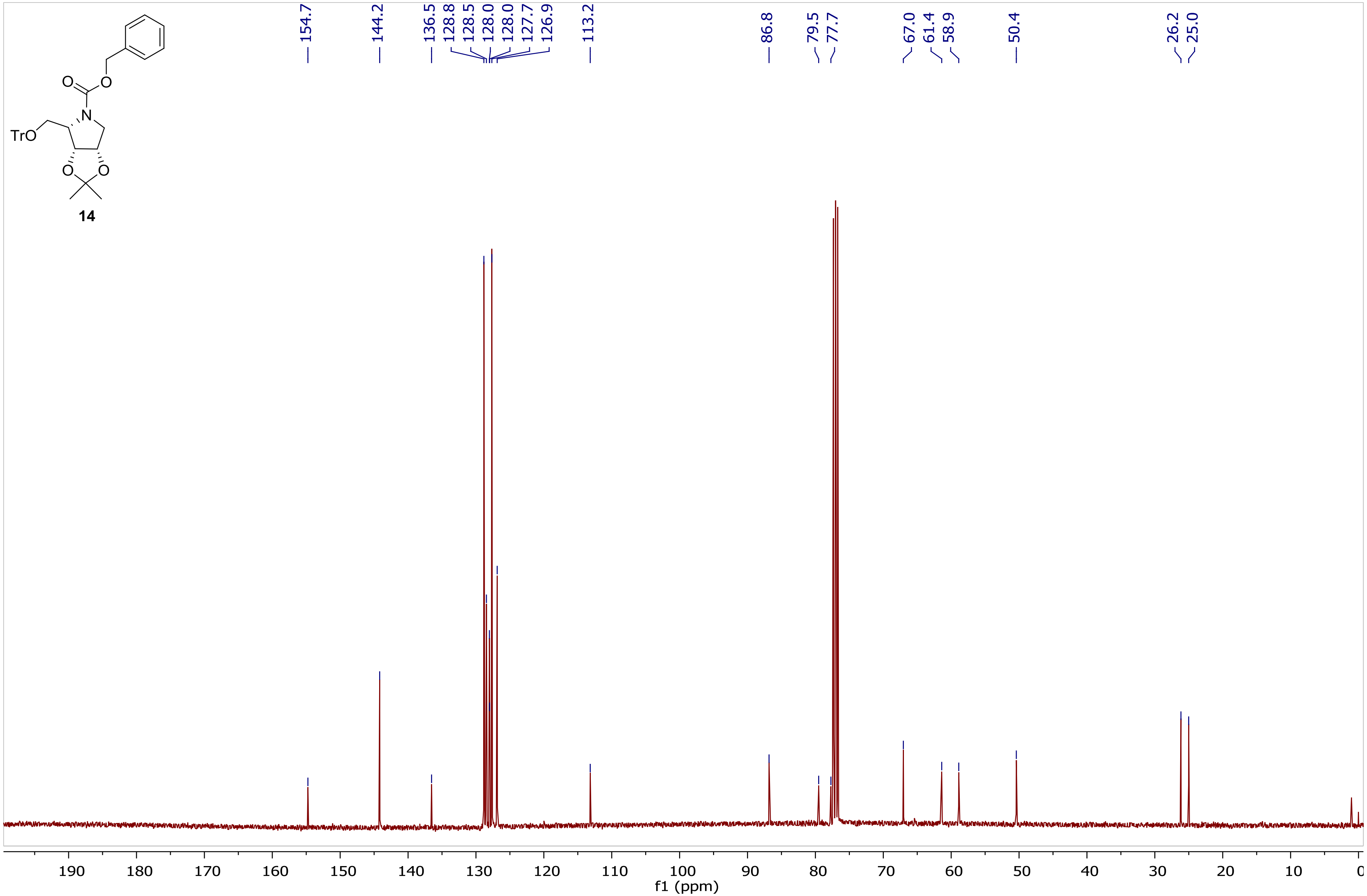

154.7
144.2
136.5
128.8
128.5
128.0
128.0
127.7
126.9
113.2
86.8
79.5
77.7
67.0
61.4
58.9
50.4
26.2
25.0
TrO
N
O
O
O
O
14
190 180 170 160 150 140 130 120 110 100 90 80 70 60 50 40 30 20 10 0
f1 (ppm)

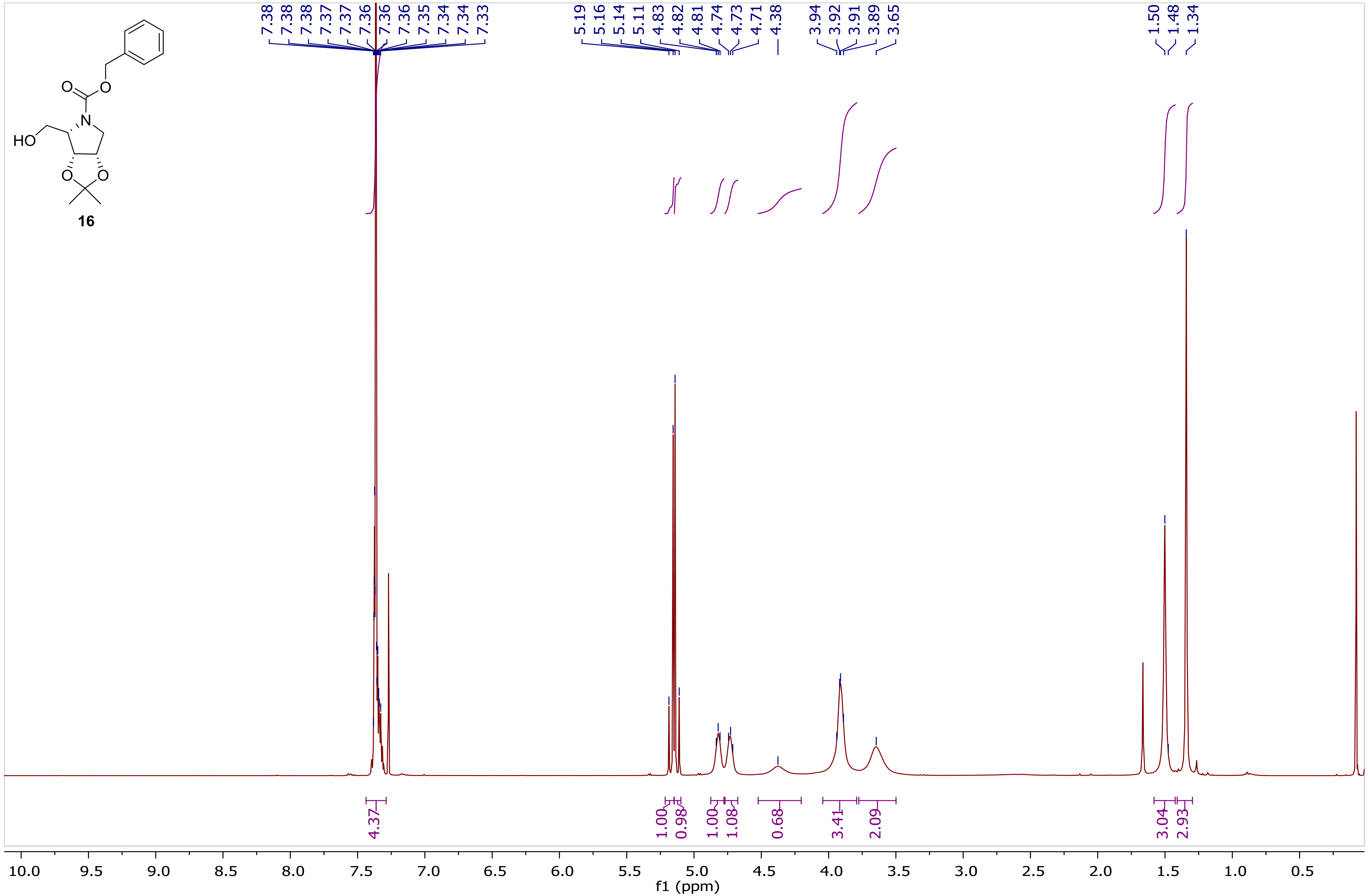
7.38
7.38
7.38
7.37
7.37
7.36
7.36
7.36
7.35
7.34
7.34
7.33
5.19
5.16
5.14
5.11
4.83
4.82
4.81
4.74
4.73
4.71
4.38
3.94
3.92
3.91
3.89
3.65
1.50
1.48
1.34
HO
O
N
O
O
O
16
4.37
1.00
0.98
1.00
1.08
0.68
3.41
2.09
3.04
2.93
10.0
9.5
9.0
8.5
8.0
7.5
7.0
6.5
6.0
5.5
5.0
4.5
4.0
3.5
3.0
2.5
2.0
1.5
1.0
0.5
f1 (ppm)

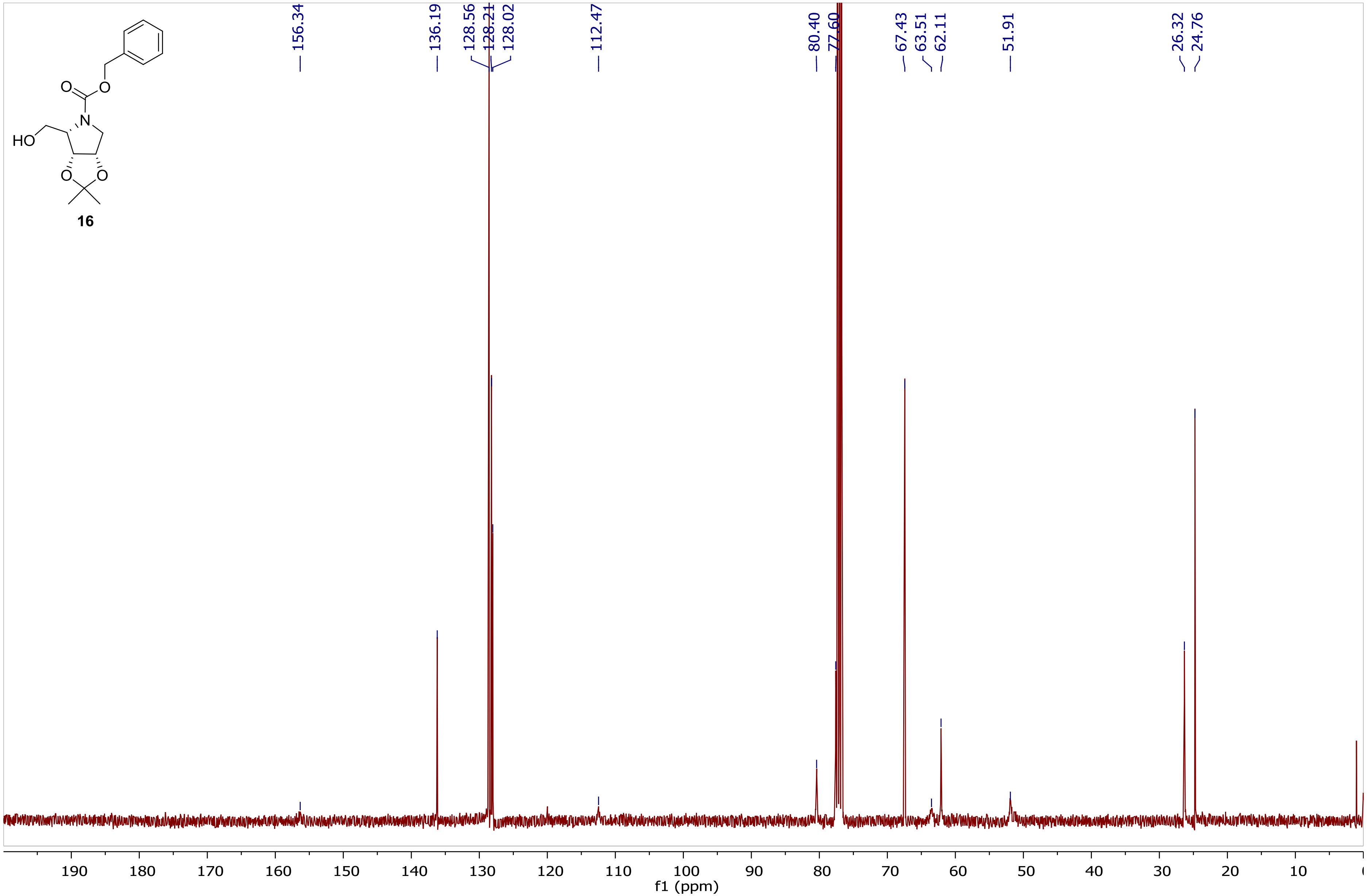
156.34
136.19
128.56
128.21
128.02
112.47
80.40
77.60
67.43
63.51
62.11
51.91
26.32
24.76
HO
O
O
N
O
O
16
190 180 170 160 150 140 130 120 110 100 90 80 70 60 50 40 30 20 10
f1 (ppm)

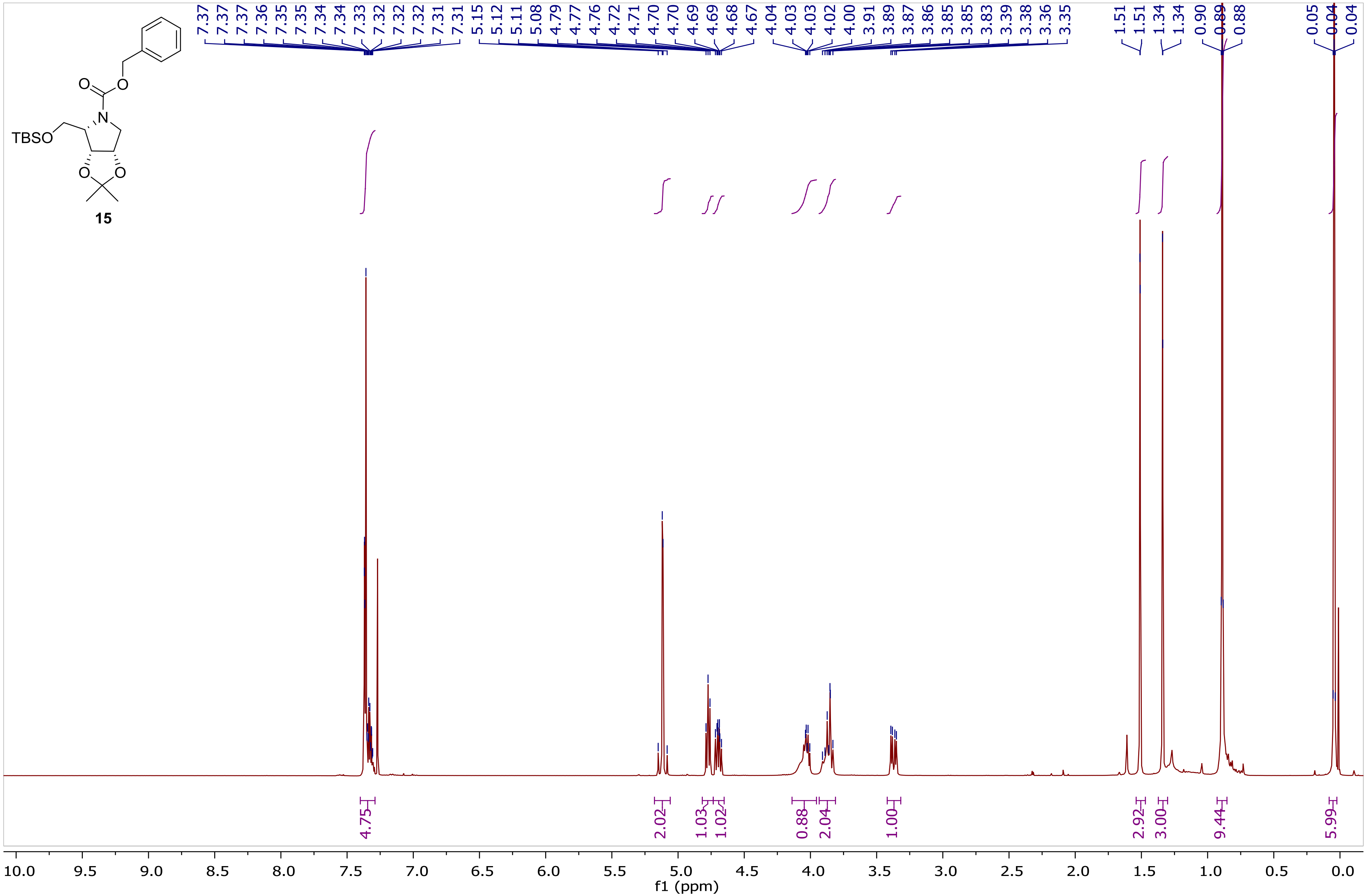
7.37 7.37 7.37 7.36 7.35 7.35 7.34 7.34 7.33 7.32 7.32 7.32 7.31 7.31 5.15 5.12 5.11 5.08 4.79 4.77 4.76 4.72 4.71 4.70 4.70 4.69 4.69 4.68 4.67 4.04 4.03 4.03 4.02 4.00 3.91 3.89 3.87 3.86 3.85 3.85 3.83 3.39 3.38 3.36 3.35
1.51 1.51 1.34 1.34 0.90 0.89 0.88 0.05 0.04 0.04
TBSO
N
O
15
4.75
2.02
1.03
1.02
0.88
2.04
1.00
2.92
3.00
9.44
5.99
10.0 9.5 9.0 8.5 8.0 7.5 7.0 6.5 6.0 5.5 5.0 4.5 4.0 3.5 3.0 2.5 2.0 1.5 1.0 0.5 0.0
f1 (ppm)

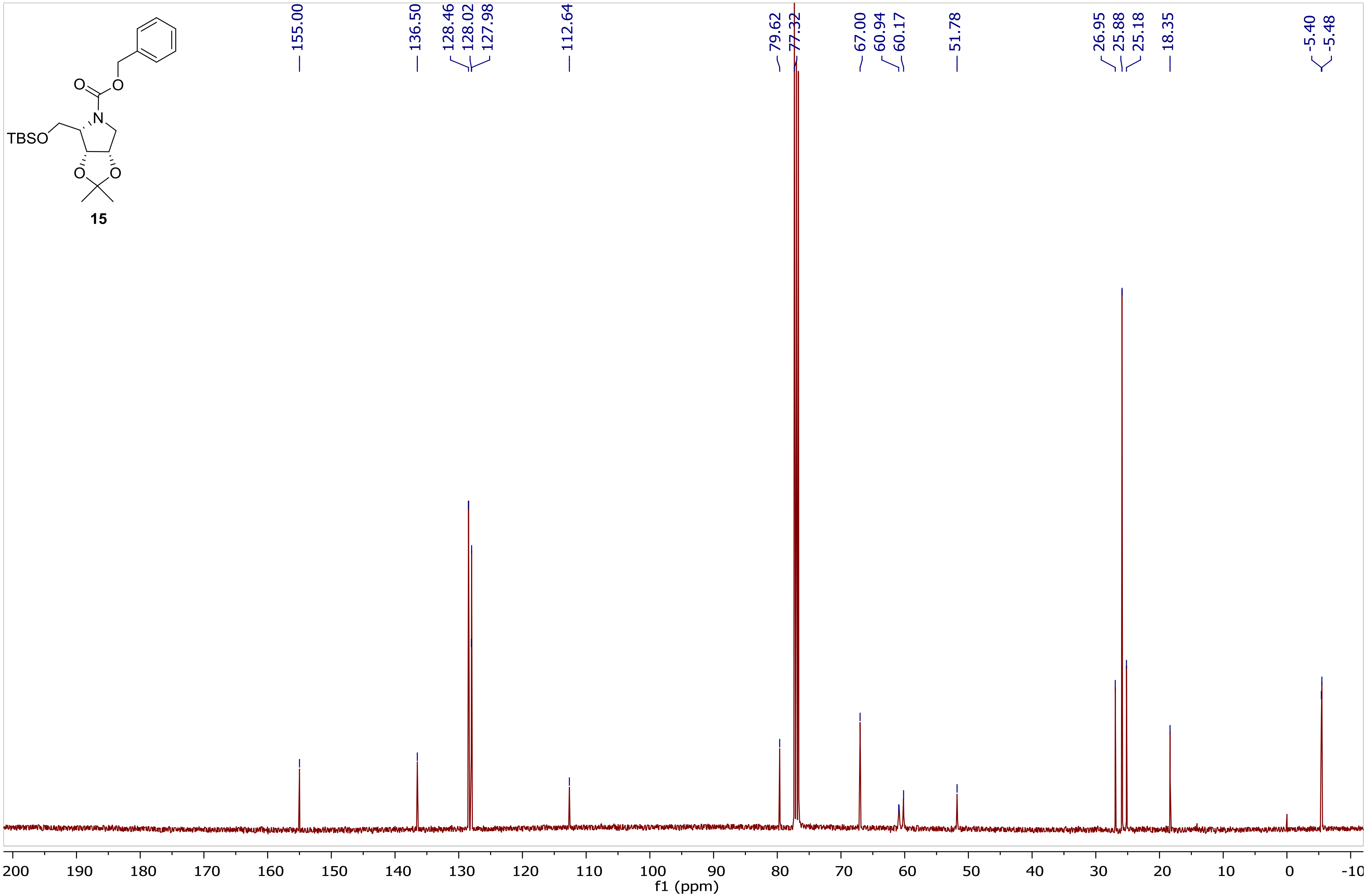

155.00
136.50
128.46
128.02
127.98
112.64
79.62
77.32
67.00
60.94
60.17
51.78
26.95
25.88
25.18
18.35
-5.40
-5.48
TBSO
15
200 190 180 170 160 150 140 130 120 110 100 90 80 70 60 50 40 30 20 10 0 -10
f1 (ppm)

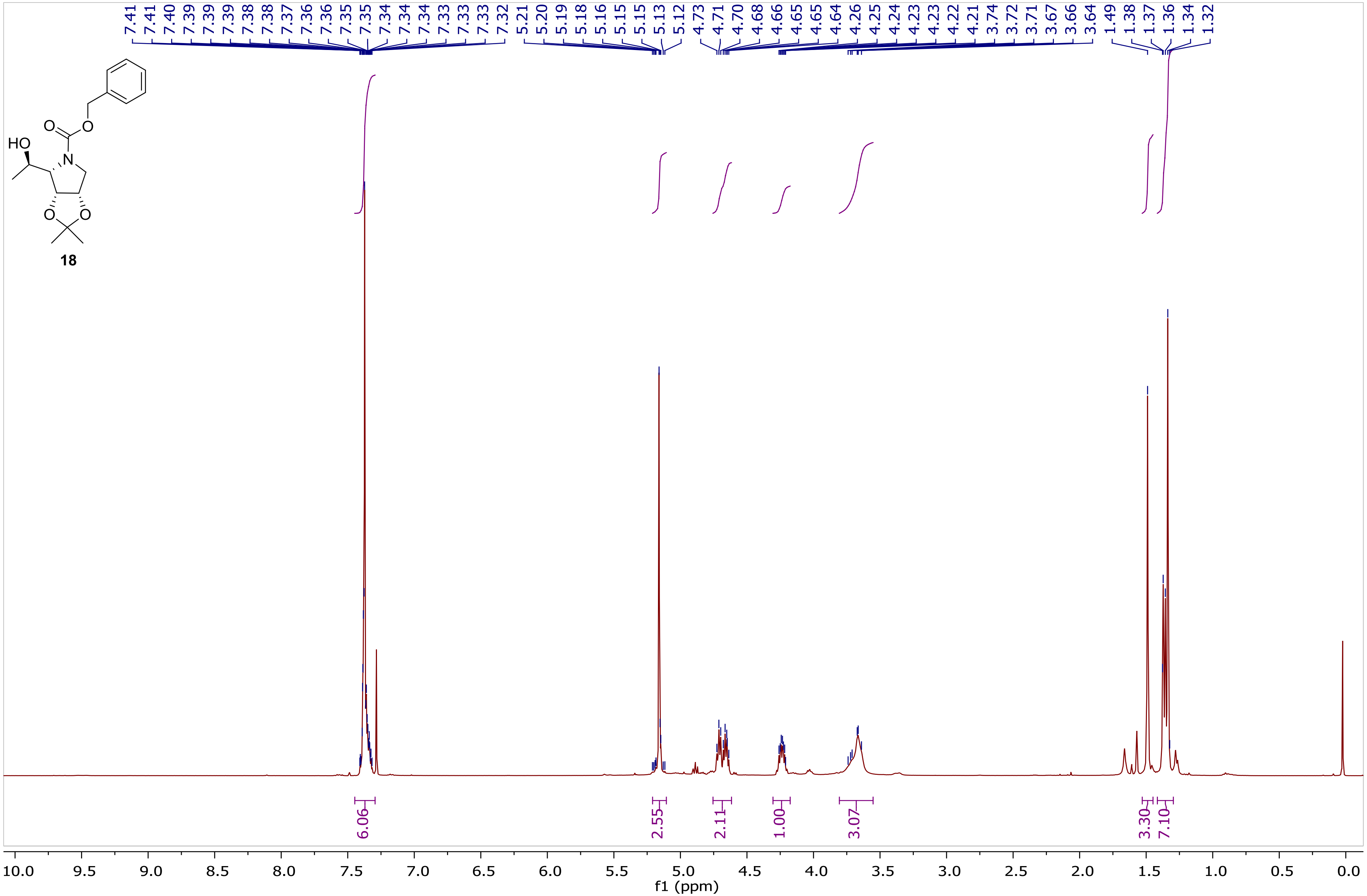

18
7.41
7.41
7.40
7.39
7.39
7.39
7.38
7.38
7.37
7.36
7.36
7.35
7.35
7.34
7.34
7.34
7.33
7.33
7.33
7.32
5.21
5.20
5.19
5.18
5.16
5.15
5.15
5.13
5.12
4.73
4.71
4.70
4.68
4.66
4.65
4.65
4.64
4.26
4.25
4.24
4.23
4.23
4.22
4.21
3.74
3.72
3.71
3.67
3.66
3.64
1.49
1.38
1.37
1.36
1.34
1.32
6.06
2.55
2.11
1.00
3.07
3.30
7.10
10.0
9.5
9.0
8.5
8.0
7.5
7.0
6.5
6.0
5.5
5.0
4.5
4.0
3.5
3.0
2.5
2.0
1.5
1.0
0.5
0.0
f1 (ppm)

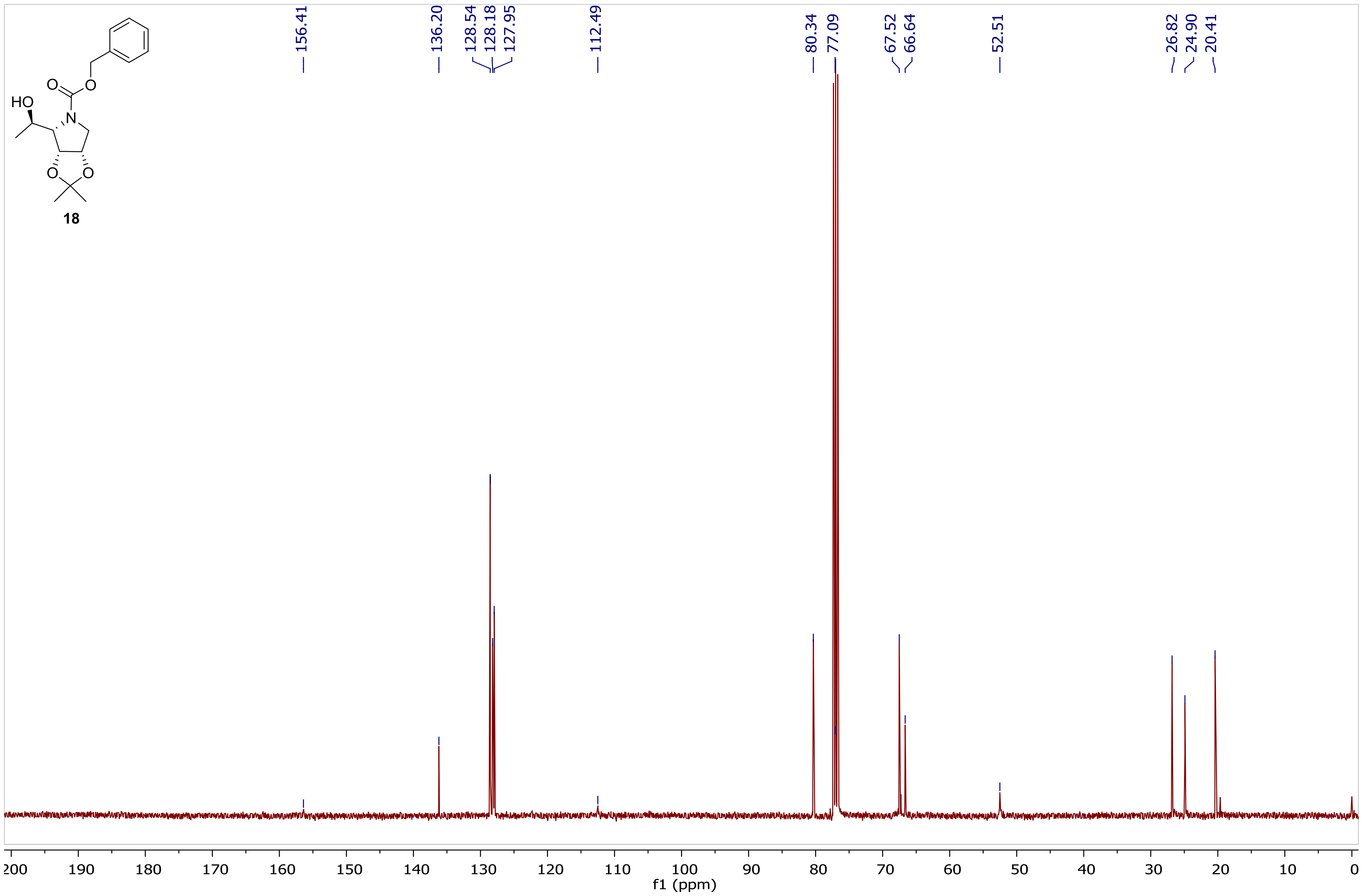

156.41
136.20
128.54
128.18
127.95
112.49
80.34
77.09
67.52
66.64
52.51
26.82
24.90
20.41
HO
O
O
N
O
O
18
200 190 180 170 160 150 140 130 120 110 100 90 80 70 60 50 40 30 20 10 0
f1 (ppm)

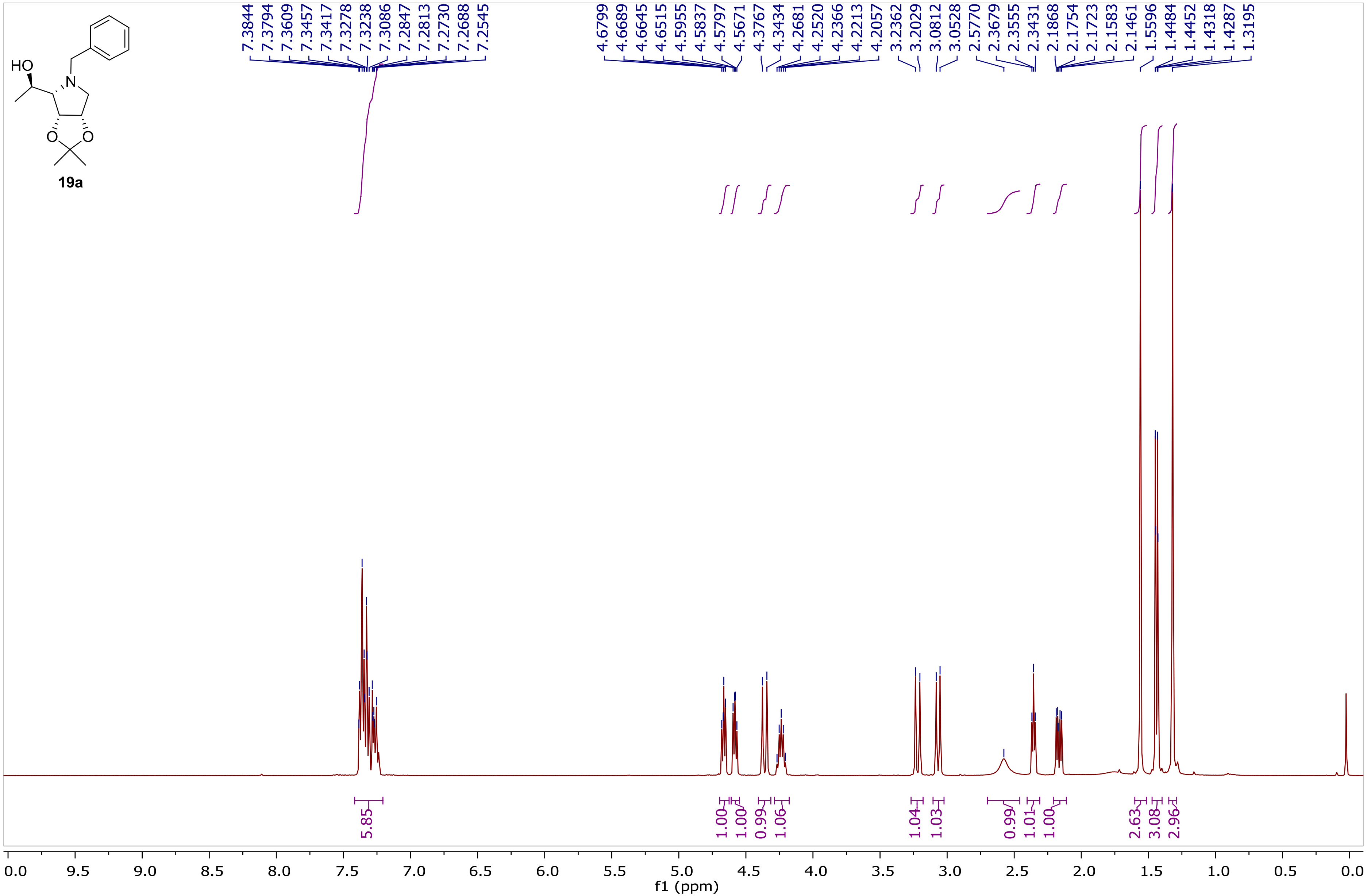

HO
N
O
O
19a
7.3844
7.3794
7.3609
7.3457
7.3417
7.3278
7.3238
7.3086
7.2847
7.2813
7.2730
7.2688
7.2545
4.6799
4.6689
4.6645
4.6515
4.5955
4.5837
4.5797
4.5671
4.3767
4.3434
4.2681
4.2520
4.2366
4.2213
4.2057
3.2362
3.2029
3.0812
3.0528
2.5770
2.3679
2.3555
2.3431
2.1868
2.1754
2.1723
2.1583
2.1461
1.5596
1.4484
1.4452
1.4318
1.4287
1.3195
5.85
1.00
1.00
0.99
1.06
1.04
1.03
0.99
1.01
1.00
2.63
3.08
2.96
f1 (ppm)

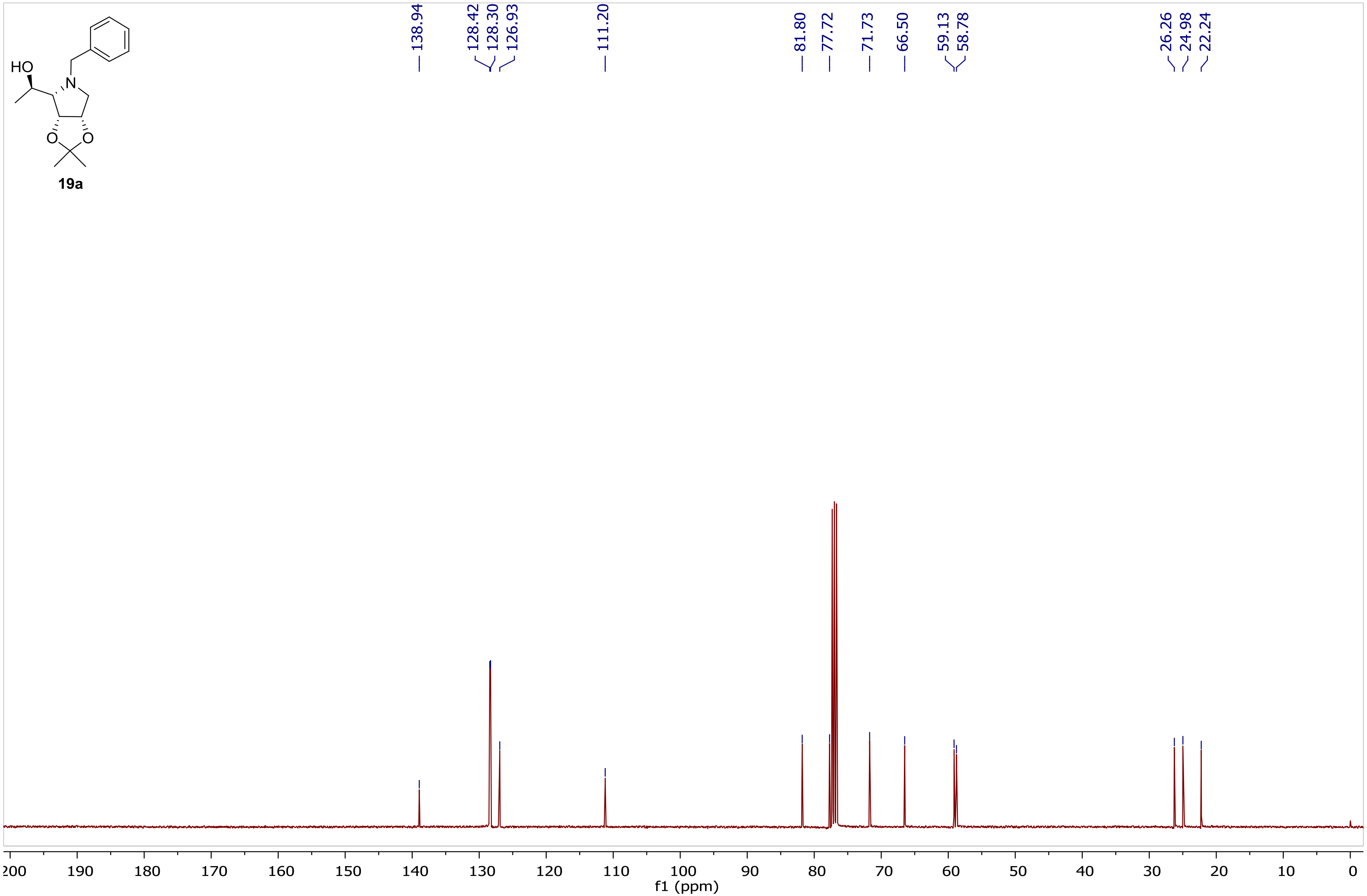

HO
N
O
O
19a
138.94
128.42
128.30
126.93
111.20
81.80
77.72
71.73
66.50
59.13
58.78
26.26
24.98
22.24
200
190
180
170
160
150
140
130
120
110
100
90
80
70
60
50
40
30
20
10
0
f1 (ppm)

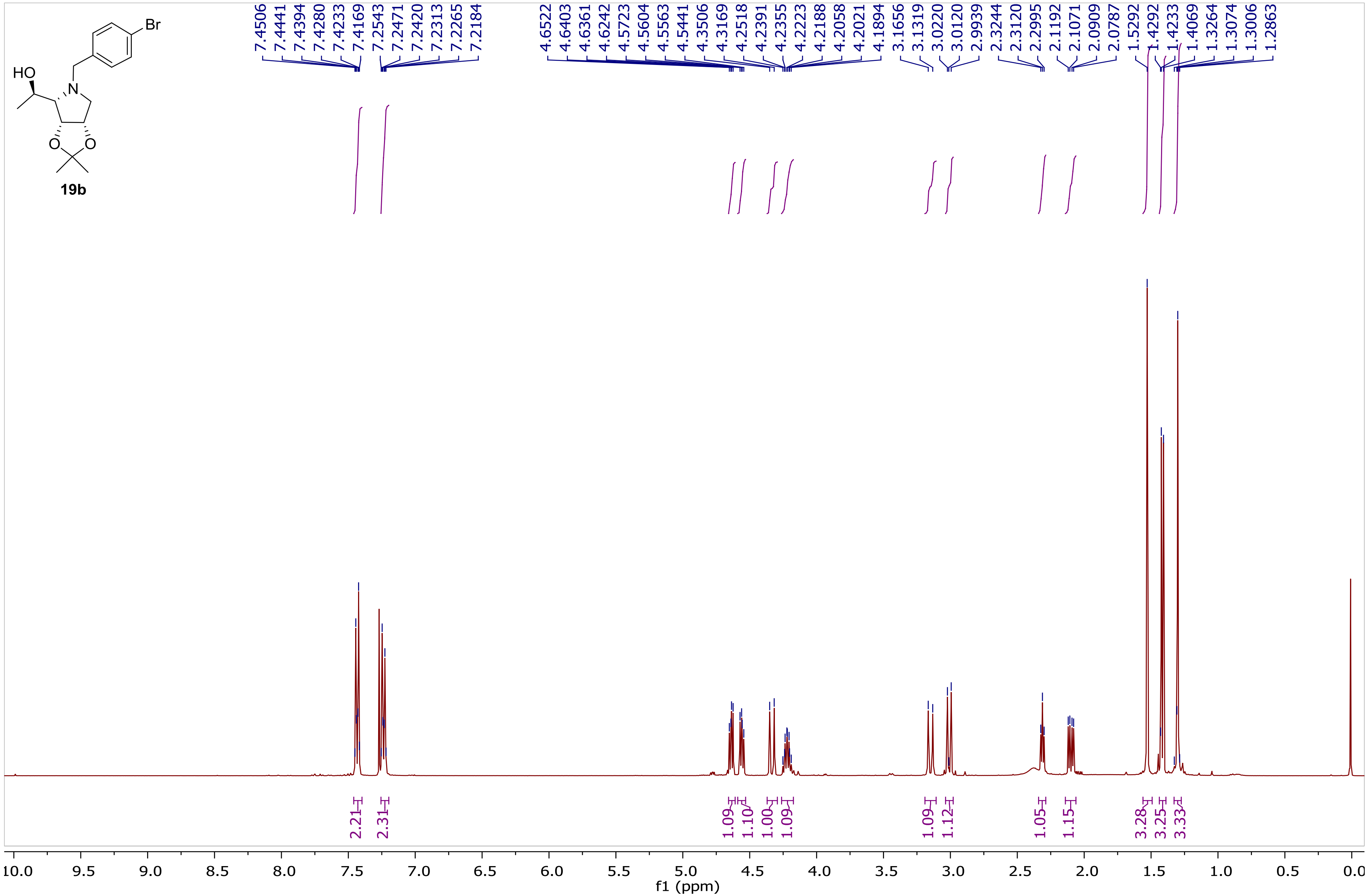
Br
HO
N
O
O
19b
f1 (ppm)

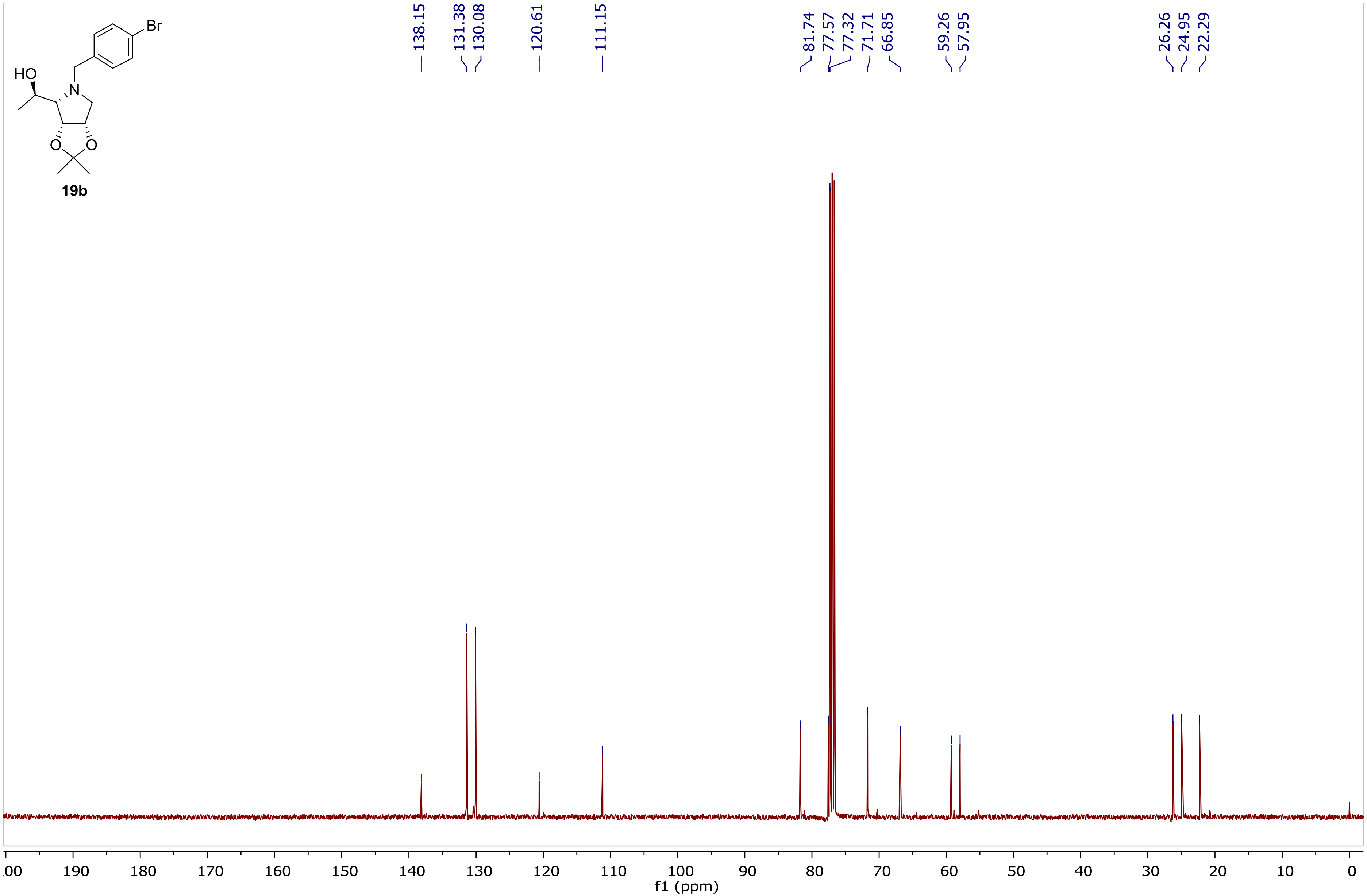
Br
HO
N
O
O
19b
138.15
131.38
130.08
120.61
111.15
81.74
77.57
77.32
71.71
66.85
59.26
57.95
26.26
24.95
22.29
00
190
180
170
160
150
140
130
120
110
100
90
80
70
60
50
40
30
20
10
0
f1 (ppm)

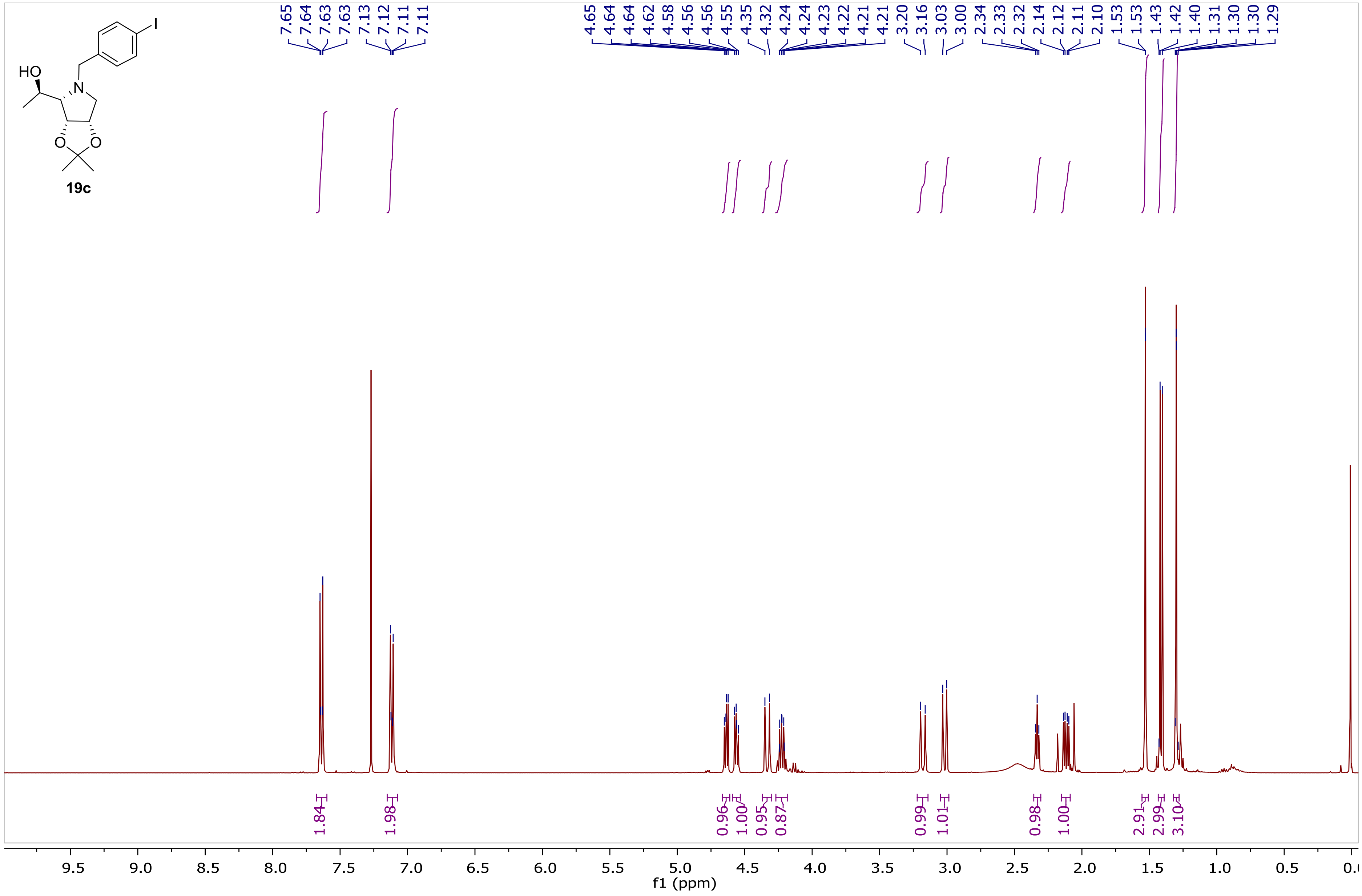
HO
N
I
O
O
19c
7.65
7.64
7.63
7.63
7.13
7.12
7.11
7.11
4.65
4.64
4.64
4.62
4.58
4.56
4.56
4.55
4.35
4.32
4.24
4.24
4.23
4.22
4.21
4.21
3.20
3.16
3.03
3.00
2.34
2.33
2.32
2.14
2.12
2.11
2.10
1.53
1.53
1.43
1.42
1.40
1.40
1.31
1.30
1.30
1.29
1.84
1.98
0.96
1.00
0.95
0.87
0.99
1.01
0.98
1.00
2.91
2.99
3.10
f1 (ppm)

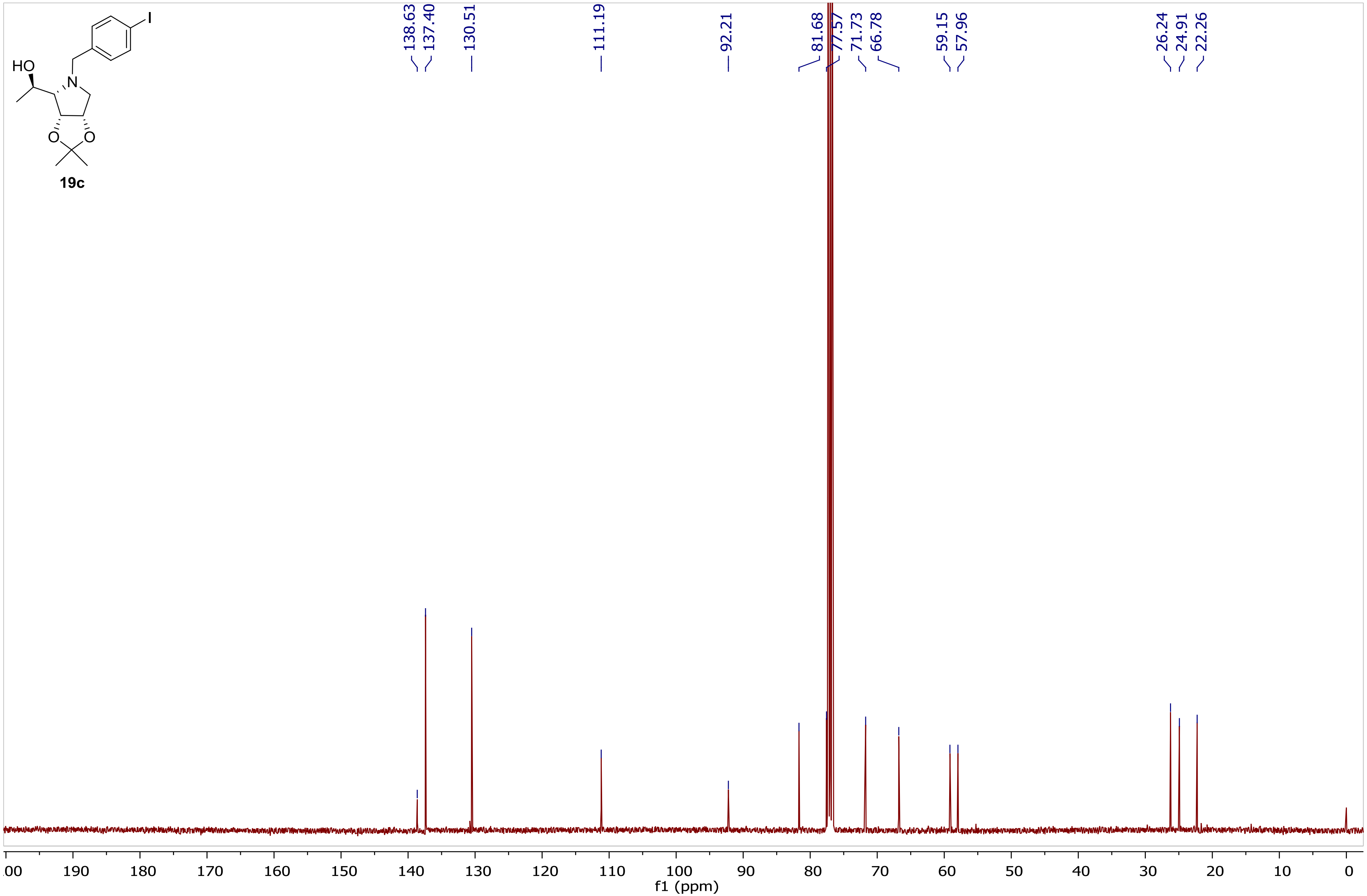

HO
N
O
O
I
19c
138.63
137.40
130.51
111.19
92.21
81.68
77.57
71.73
66.78
59.15
57.96
26.24
24.91
22.26
f1 (ppm)

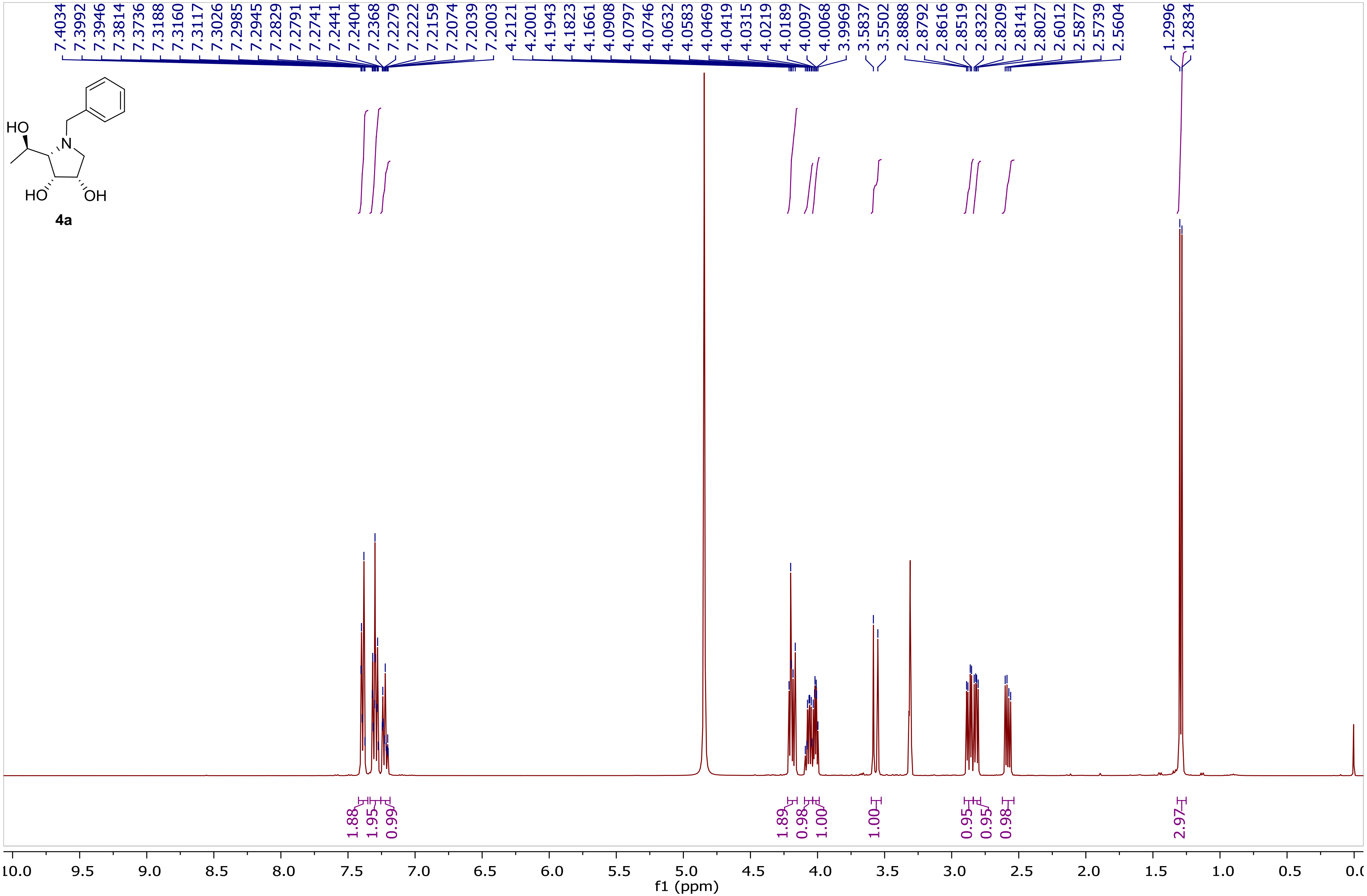
7.4034
7.3992
7.3946
7.3814
7.3736
7.3188
7.3160
7.3117
7.3026
7.2985
7.2945
7.2829
7.2791
7.2741
7.2441
7.2404
7.2368
7.2279
7.2222
7.2159
7.2074
7.2039
7.2003
4.2121
4.2001
4.1943
4.1823
4.1661
4.0908
4.0797
4.0746
4.0632
4.0583
4.0469
4.0419
4.0315
4.0219
4.0189
4.0097
4.0068
3.9969
3.5837
3.5502
2.8888
2.8792
2.8616
2.8519
2.8322
2.8209
2.8141
2.8027
2.6012
2.5877
2.5739
2.5604
1.2996
1.2834
HO
N
HO
OH
4a
1.88
1.95
0.99
1.89
0.98
1.00
1.00
0.95
0.95
0.98
2.97
10.0 9.5 9.0 8.5 8.0 7.5 7.0 6.5 6.0 5.5 5.0 4.5 4.0 3.5 3.0 2.5 2.0 1.5 1.0 0.5 0.0
f1 (ppm)

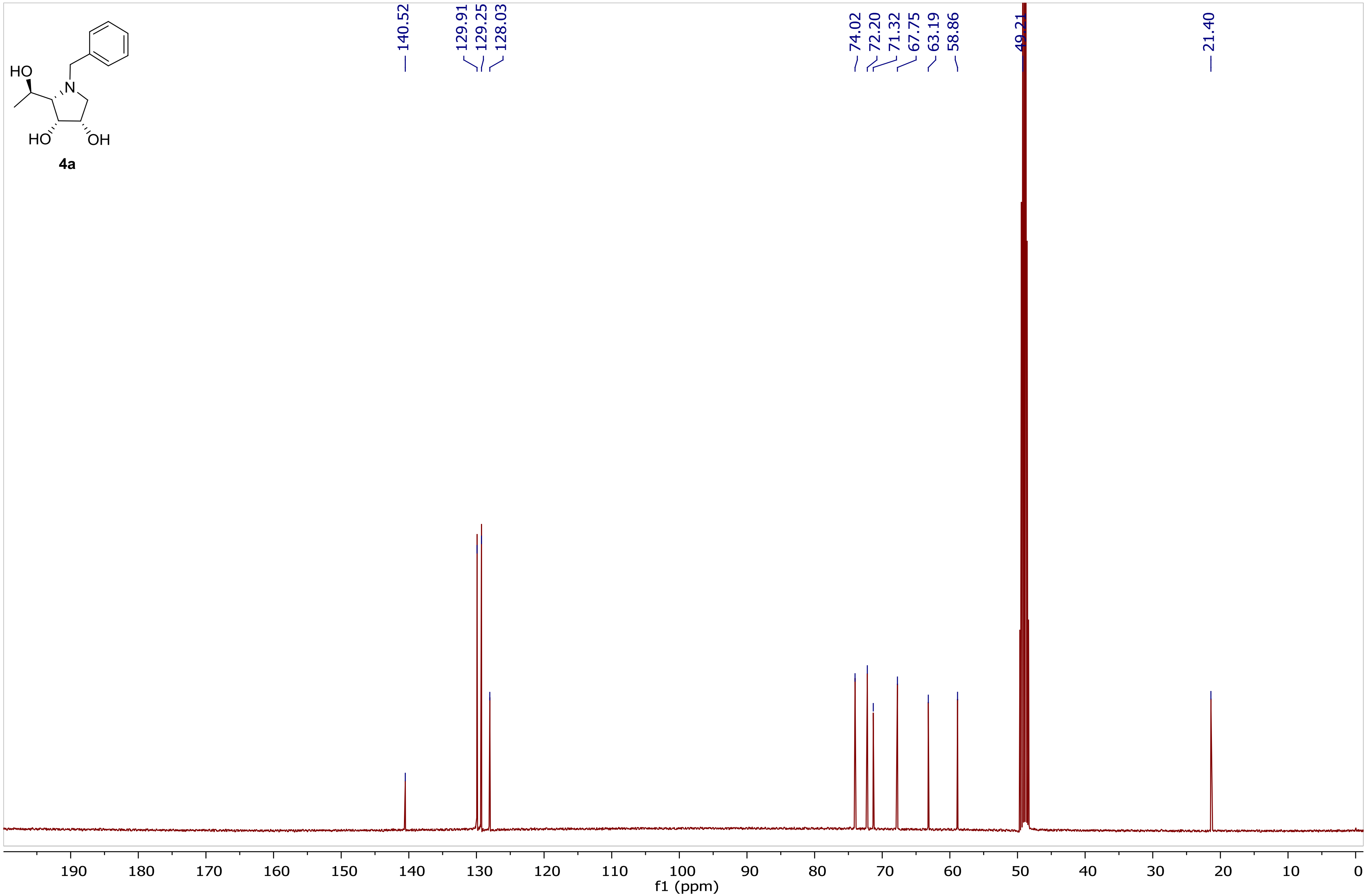

140.52
129.91
129.25
128.03
74.02
72.20
71.32
67.75
63.19
58.86
49.21
21.40
HO
N
HO
OH
4a
190 180 170 160 150 140 130 120 110 100 90 80 70 60 50 40 30 20 10 0
f1 (ppm)

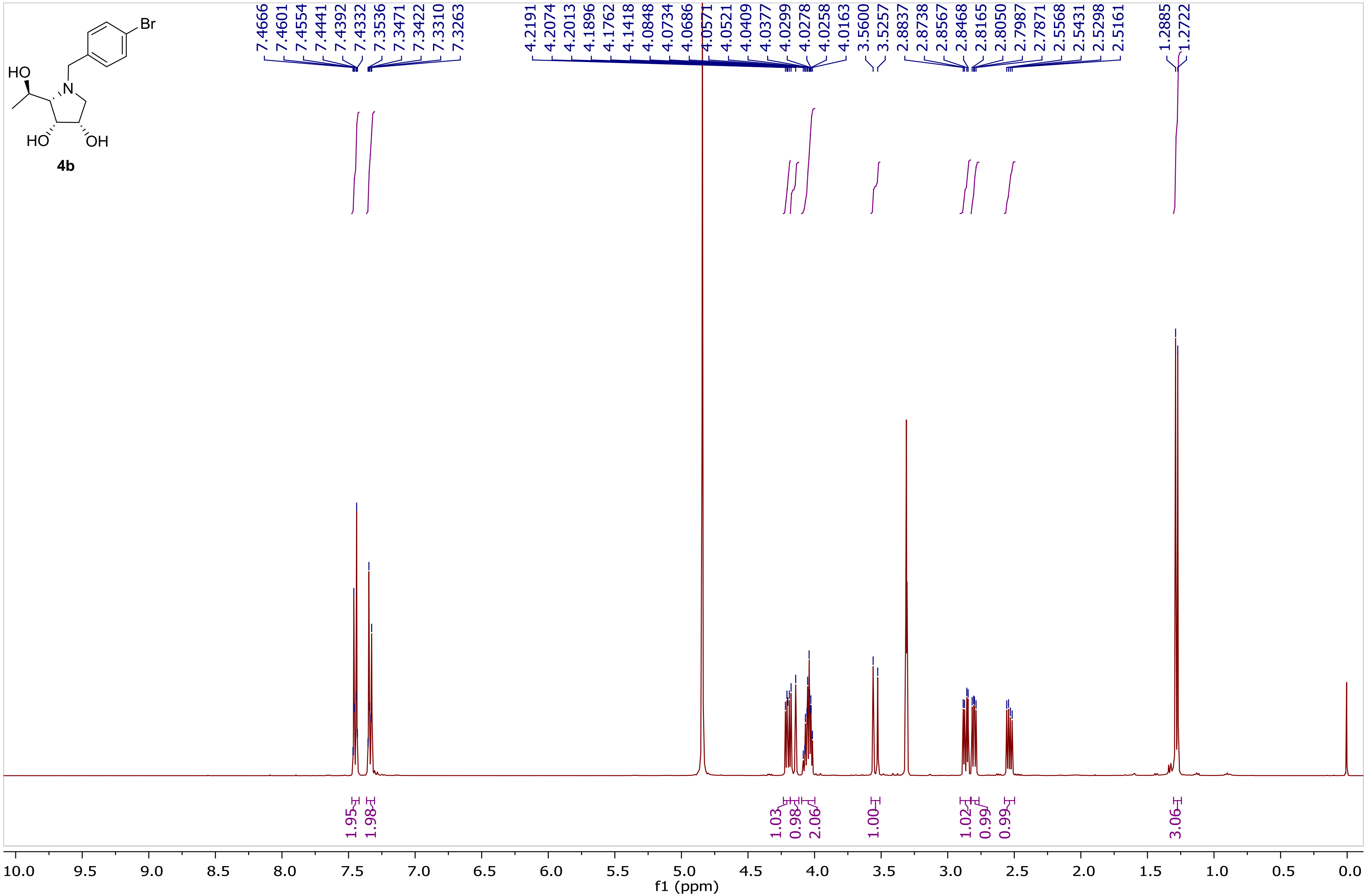

Br
HO
N
HO
OH
4b
7.4666 7.4601 7.4554 7.4441 7.4392 7.4332 7.3536 7.3471 7.3422 7.3310 7.3263
4.2191 4.2074 4.2013 4.1896 4.1762 4.1418 4.0848 4.0734 4.0686 4.0571 4.0521 4.0409 4.0377 4.0299 4.0278 4.0258 4.0163
3.5600 3.5257
2.8837 2.8738 2.8567 2.8468 2.8165 2.8050 2.7987 2.7871 2.5568 2.5431 2.5298 2.5161
1.2885 1.2722
1.95 1.98 1.03 0.98 2.06 1.00 1.02 0.99 0.99 3.06
10.0 9.5 9.0 8.5 8.0 7.5 7.0 6.5 6.0 5.5 5.0 4.5 4.0 3.5 3.0 2.5 2.0 1.5 1.0 0.5 0.0
f1 (ppm)

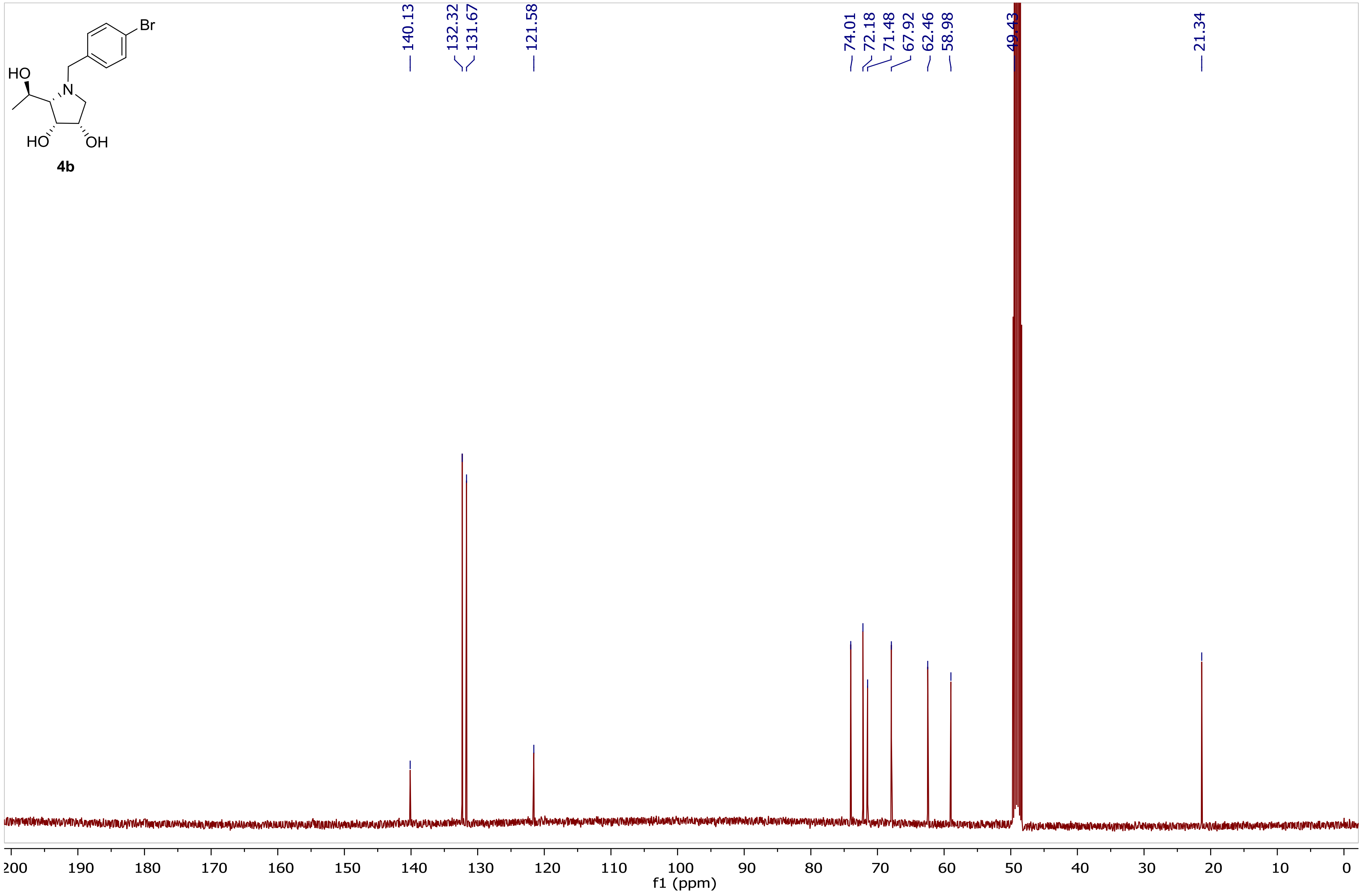
Br
HO
N
HO
OH
4b
140.13
132.32
131.67
121.58
74.01
72.18
71.48
67.92
62.46
58.98
49.43
21.34
200 190 180 170 160 150 140 130 120 110 100 90 80 70 60 50 40 30 20 10 0
f1 (ppm)

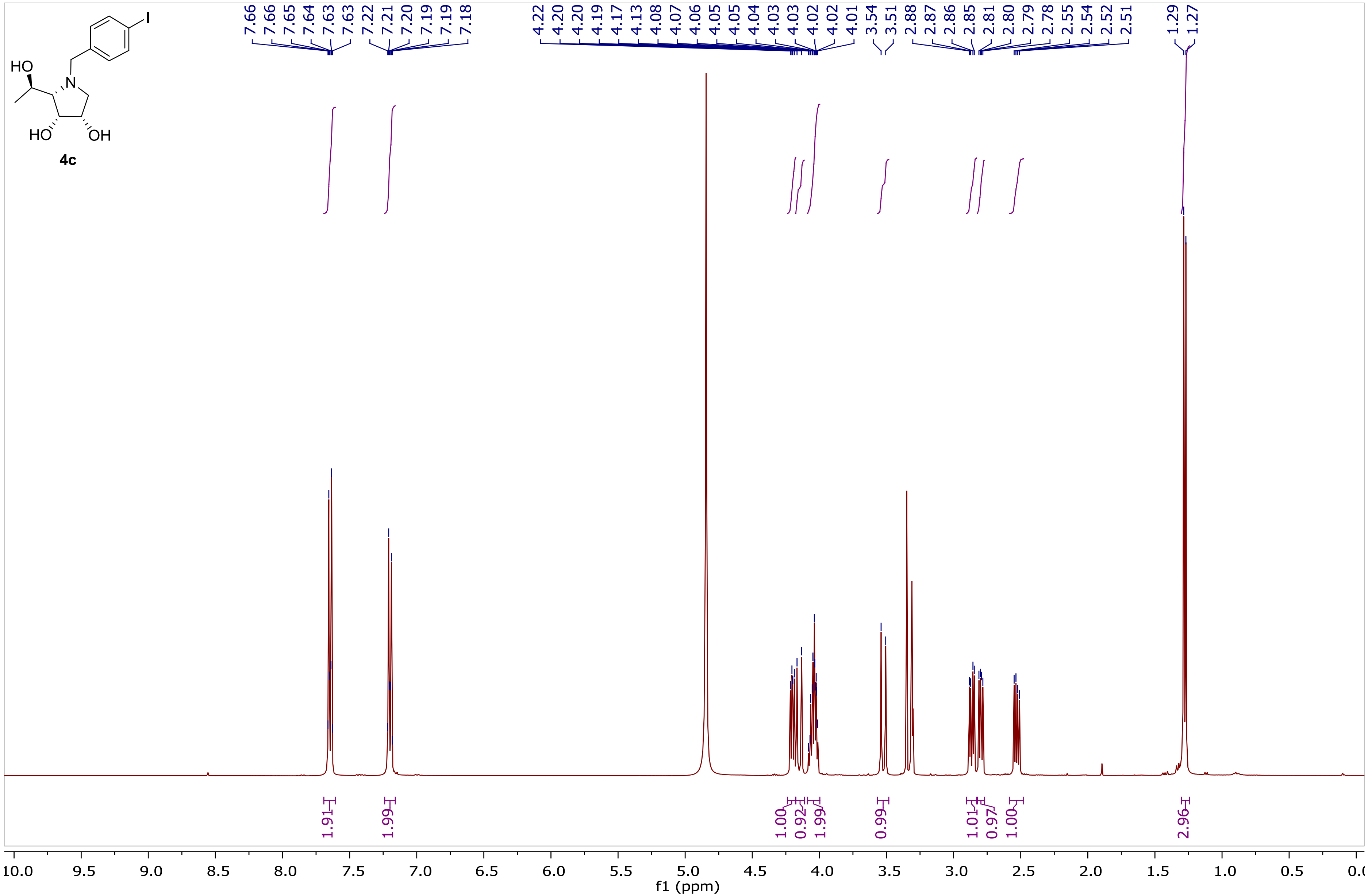

HO
N
I
HO
OH
4c
7.66 7.66 7.65 7.64 7.63 7.63 7.22 7.21 7.20 7.19 7.19 7.18
4.22 4.20 4.20 4.19 4.17 4.13 4.08 4.07 4.06 4.05 4.05 4.04 4.03 4.03 4.02 4.02 4.01 3.54 3.51 2.88 2.87 2.86 2.85 2.81 2.80 2.79 2.78 2.55 2.54 2.52 2.51
1.29 1.27
1.91 1.99 1.00 0.92 1.99 0.99 1.01 0.97 1.00 2.96
10.0 9.5 9.0 8.5 8.0 7.5 7.0 6.5 6.0 5.5 5.0 4.5 4.0 3.5 3.0 2.5 2.0 1.5 1.0 0.5
f1 (ppm)

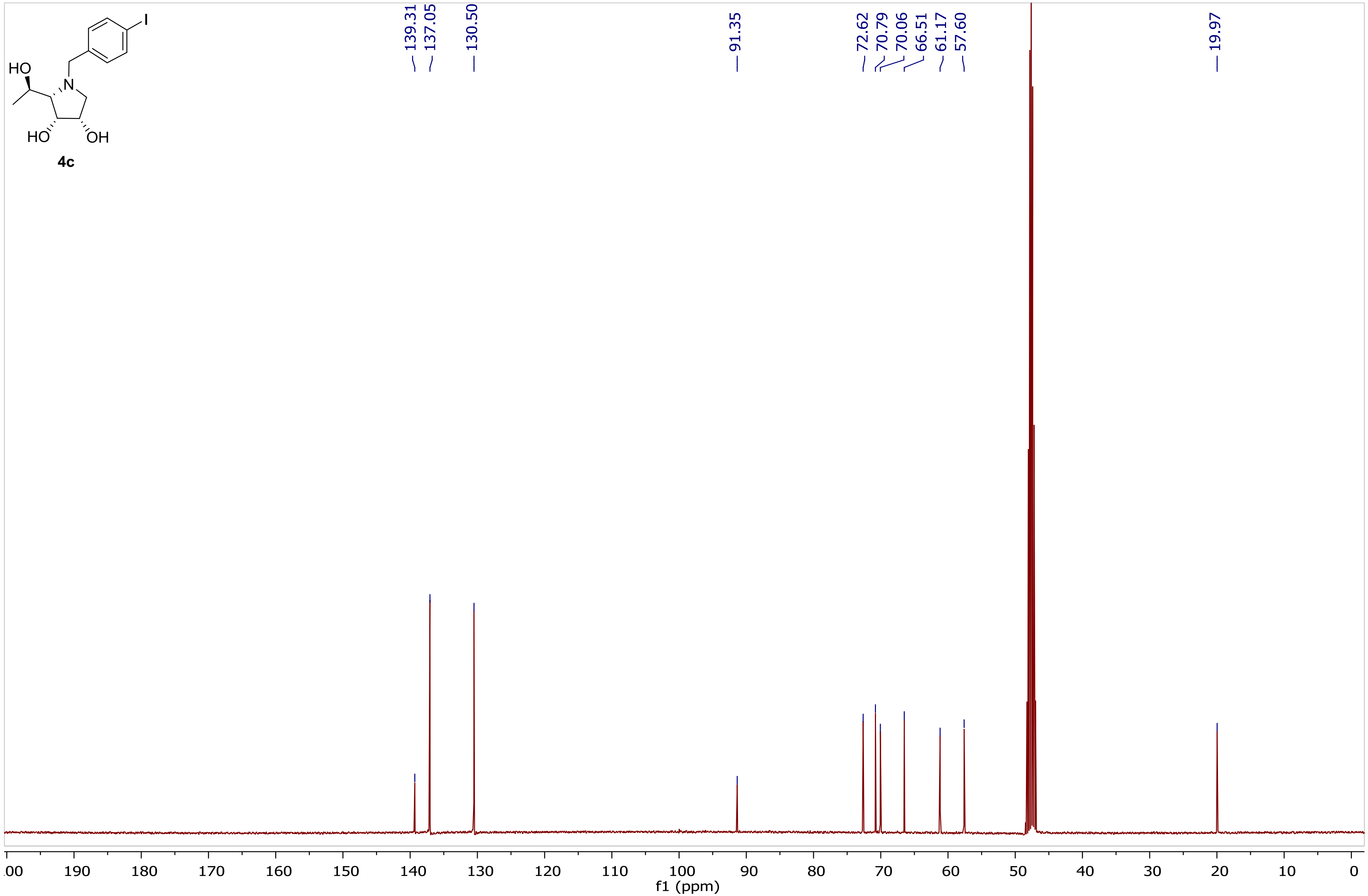
HO
N
I
HO
OH
4c
139.31
137.05
130.50
91.35
72.62
70.79
70.06
66.51
61.17
57.60
19.97
f1 (ppm)

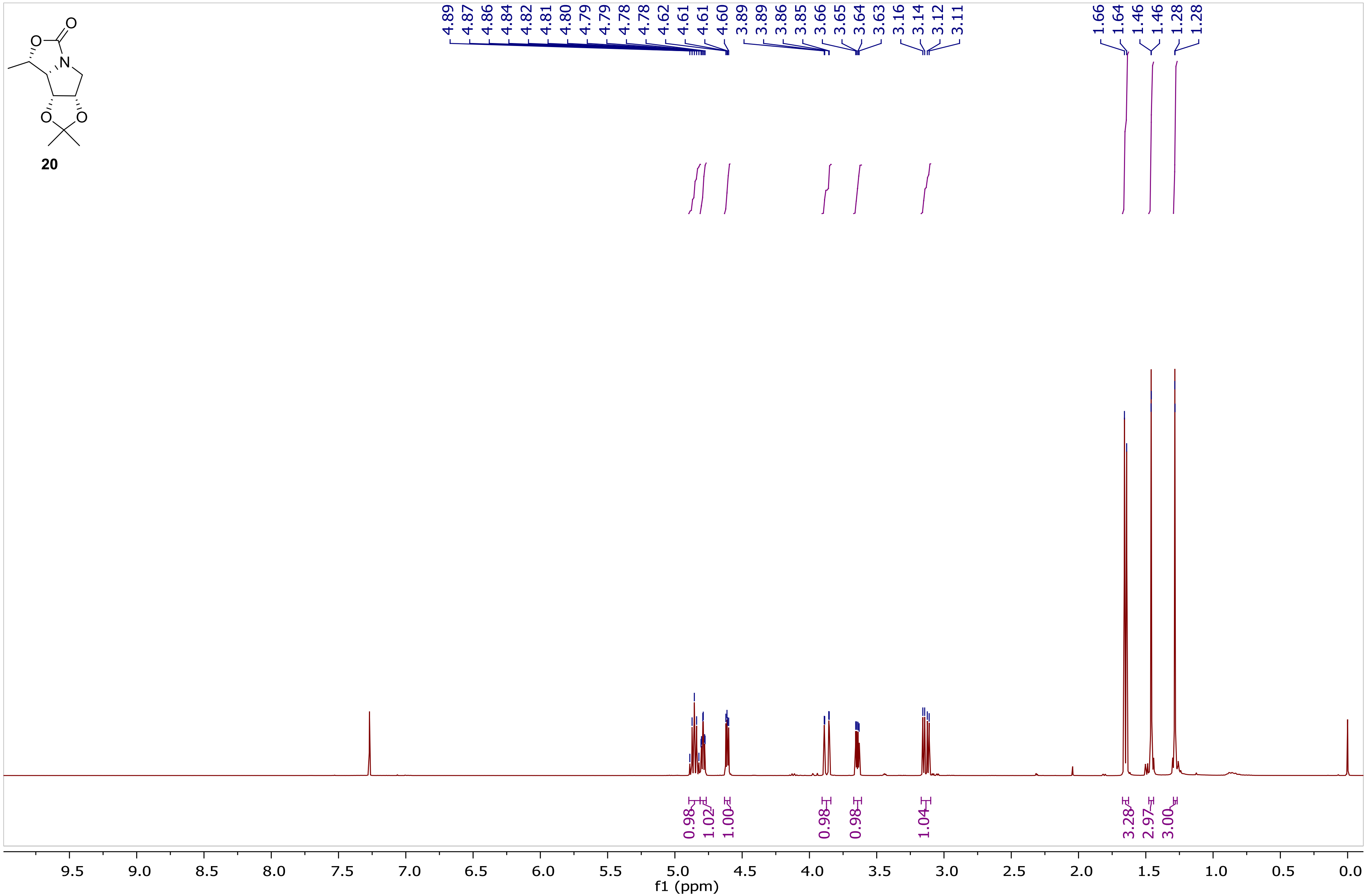
20
4.89
4.87
4.86
4.84
4.82
4.81
4.80
4.79
4.79
4.78
4.78
4.62
4.61
4.61
4.60
3.89
3.89
3.86
3.85
3.66
3.65
3.64
3.63
3.16
3.14
3.12
3.11
1.66
1.64
1.46
1.46
1.28
1.28
0.98
1.02
1.00
0.98
0.98
1.04
3.28
2.97
3.00
f1 (ppm)

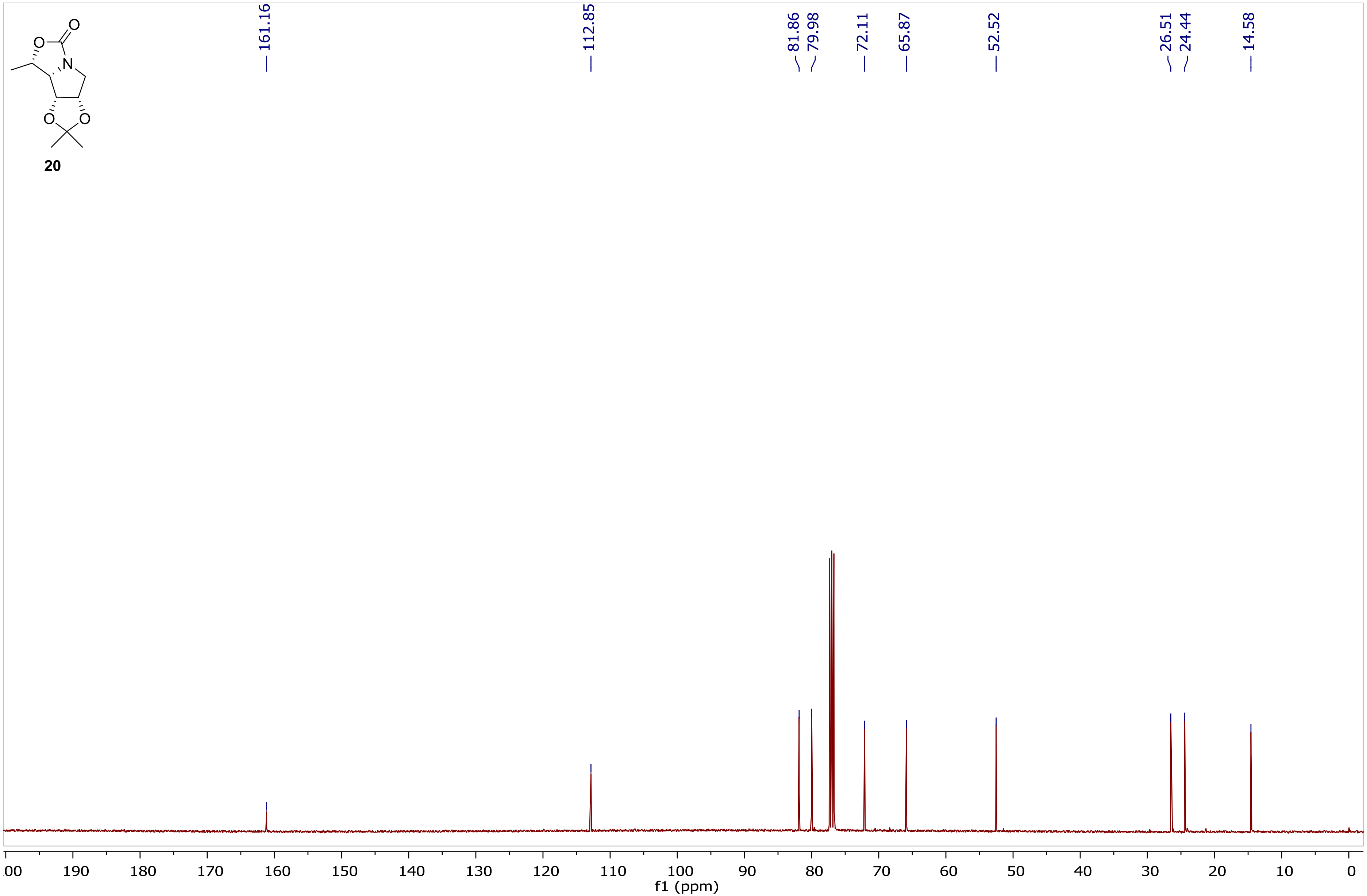
20
161.16
112.85
81.86
79.98
72.11
65.87
52.52
26.51
24.44
14.58
00 190 180 170 160 150 140 130 120 110 100 90 80 70 60 50 40 30 20 10 0
f1 (ppm)

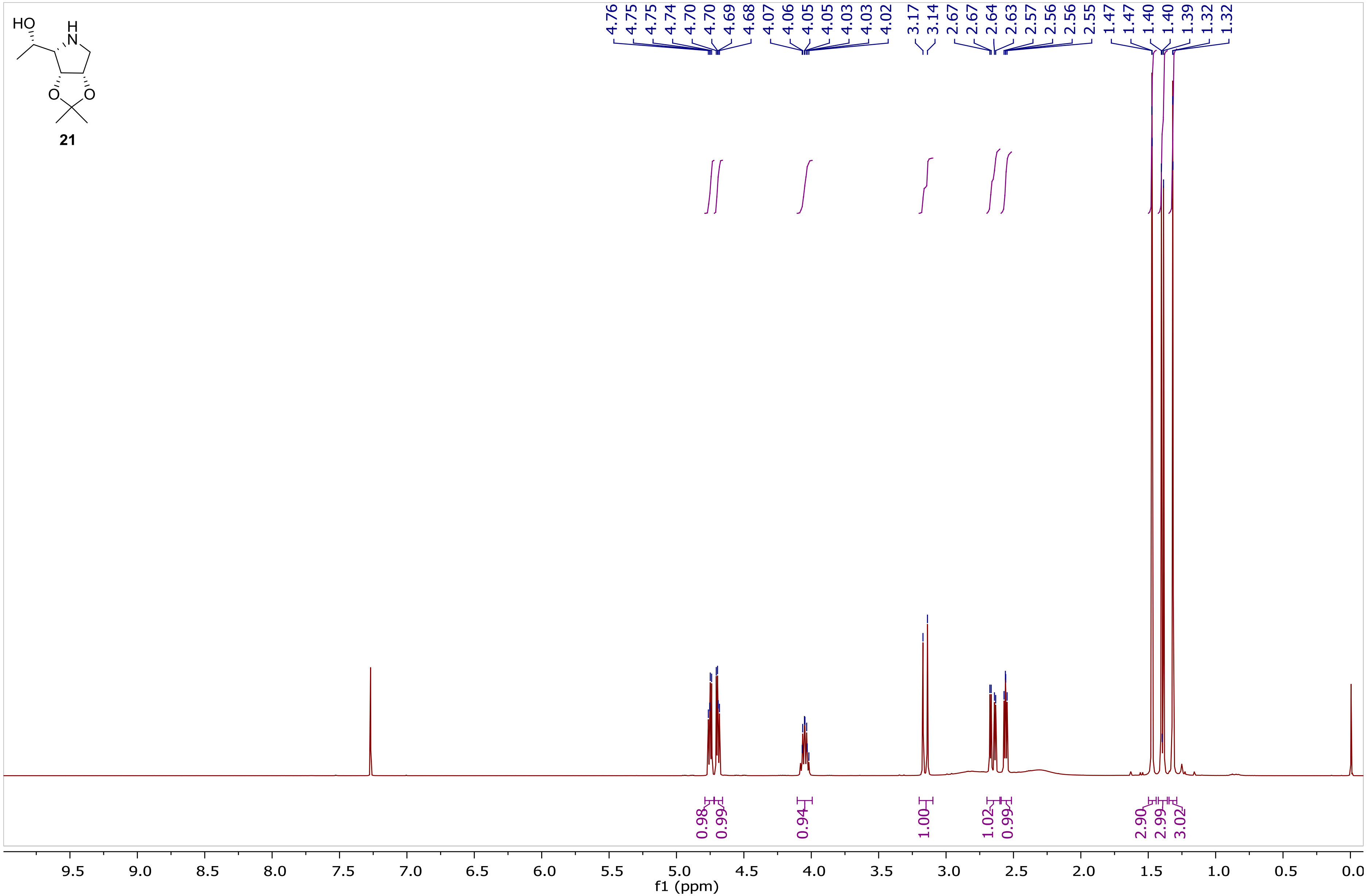
21
4.76 4.75 4.75 4.74 4.70 4.70 4.69 4.68 4.07 4.06 4.05 4.05 4.03 4.03 4.02 3.17 3.14 2.67 2.67 2.64 2.63 2.57 2.56 2.56 2.55 1.47 1.47 1.40 1.40 1.39 1.32 1.32
0.98 0.99 0.94 1.00 1.02 0.99 2.90 2.99 3.02
f1 (ppm)

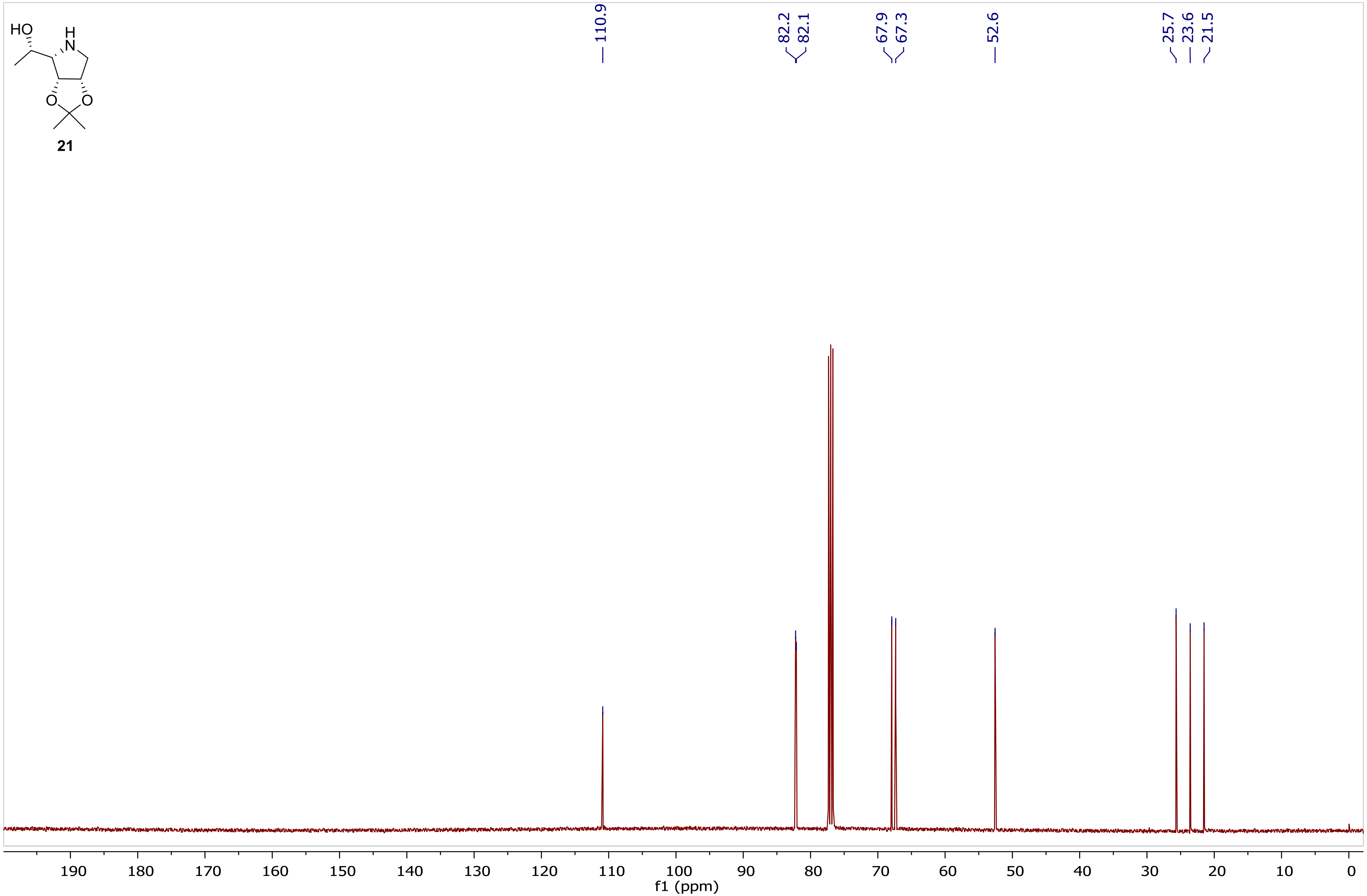
HO
H
N
O
O
21
110.9
82.2
82.1
67.9
67.3
52.6
25.7
23.6
21.5
190 180 170 160 150 140 130 120 110 100 90 80 70 60 50 40 30 20 10 0
f1 (ppm)

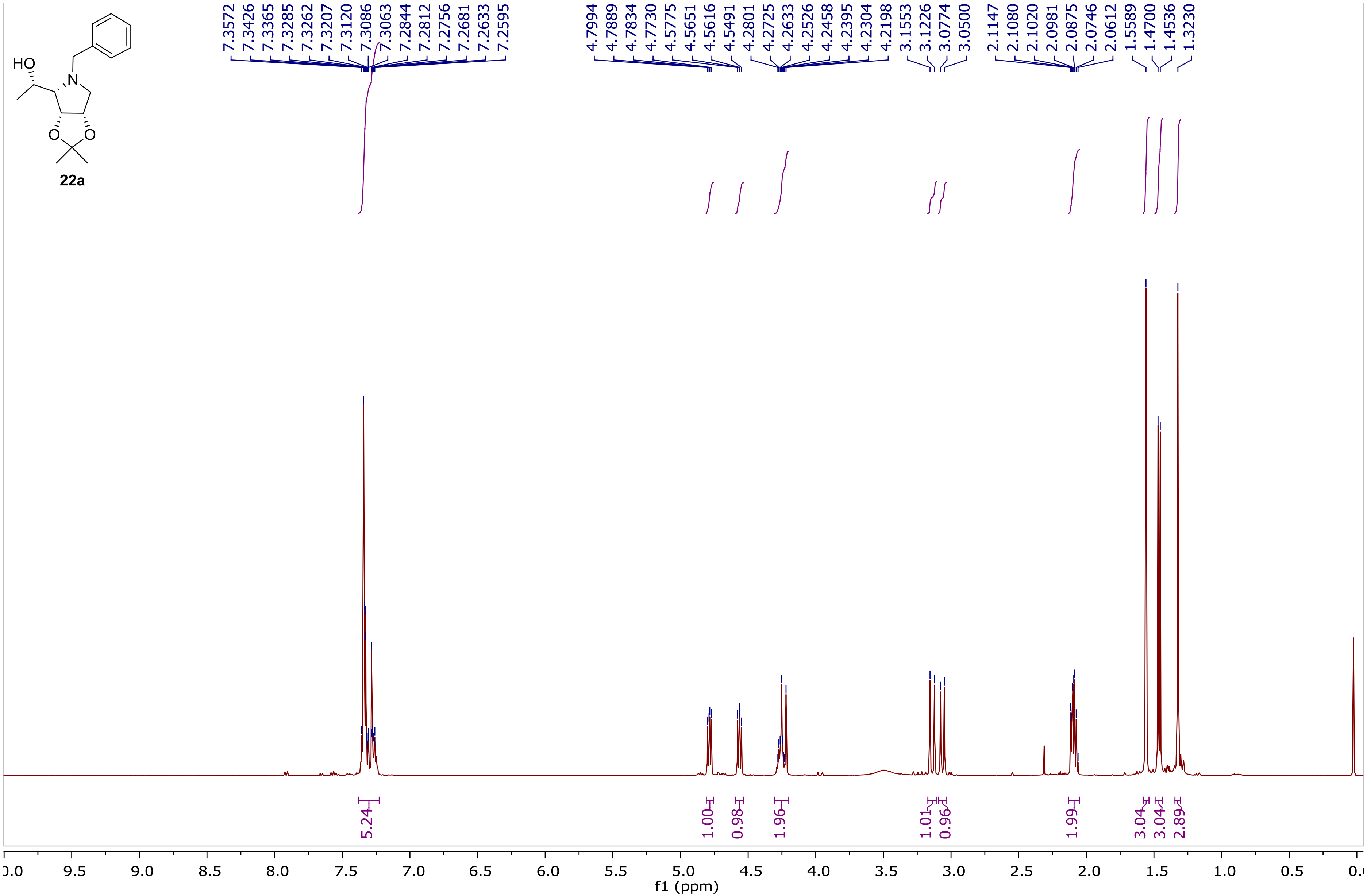
7.3572
7.3426
7.3365
7.3285
7.3262
7.3207
7.3120
7.3086
7.3063
7.2844
7.2812
7.2756
7.2681
7.2633
7.2595
4.7994
4.7889
4.7834
4.7730
4.5775
4.5651
4.5616
4.5491
4.2801
4.2725
4.2633
4.2526
4.2458
4.2395
4.2304
4.2198
3.1553
3.1226
3.0774
3.0500
2.1147
2.1080
2.1020
2.0981
2.0875
2.0746
2.0612
1.5589
1.4700
1.4536
1.3230
HO
N
O
O
22a
5.24
1.00
0.98
1.96
1.01
0.96
1.99
3.04
3.04
2.89
9.5
9.0
8.5
8.0
7.5
7.0
6.5
6.0
5.5
5.0
4.5
4.0
3.5
3.0
2.5
2.0
1.5
1.0
0.5
f1 (ppm)

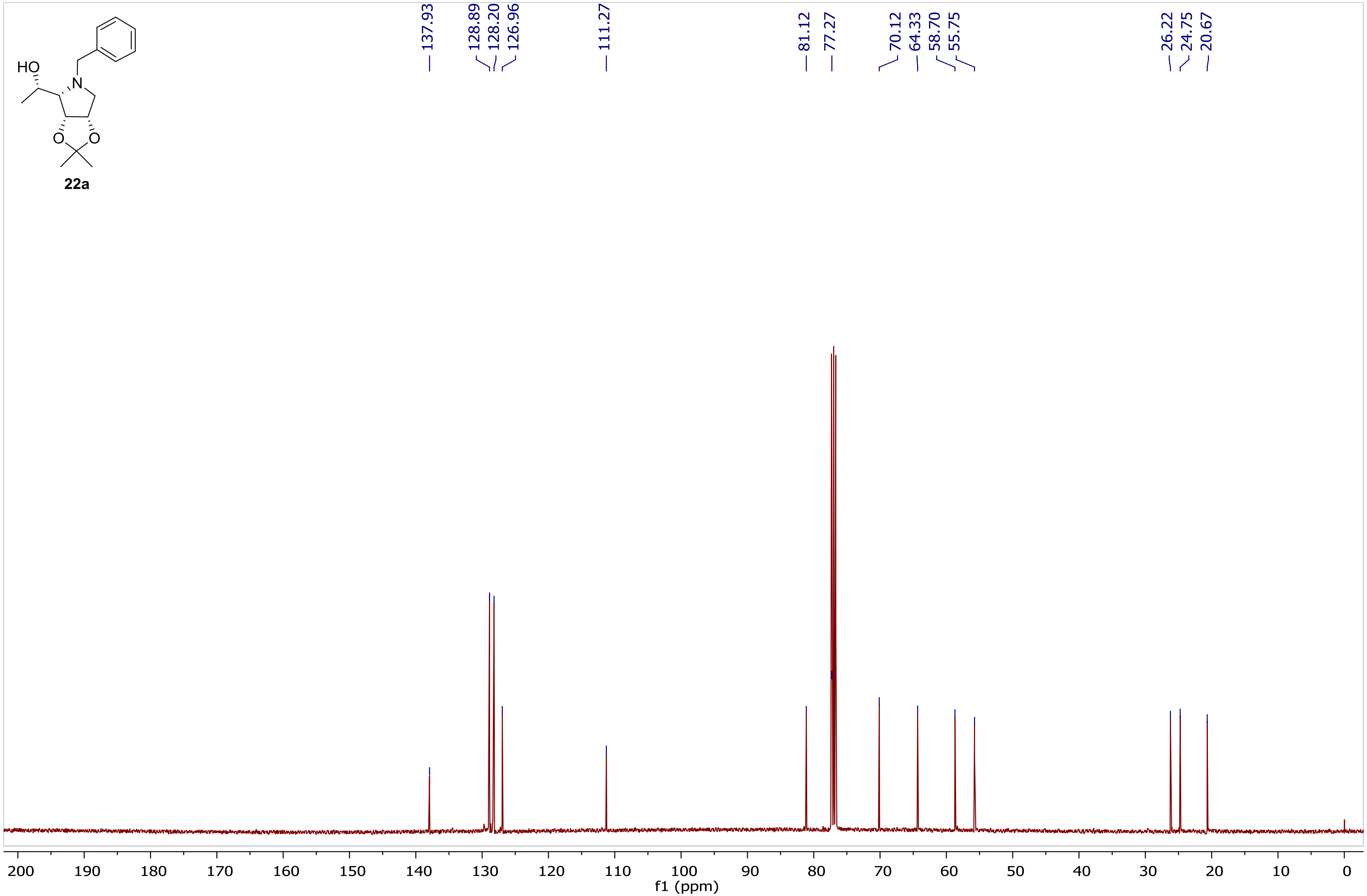

137.93
128.89
128.20
126.96
111.27
81.12
77.27
70.12
64.33
58.70
55.75
26.22
24.75
20.67
HO
N
O
O
22a
200 190 180 170 160 150 140 130 120 110 100 90 80 70 60 50 40 30 20 10 0
f1 (ppm)

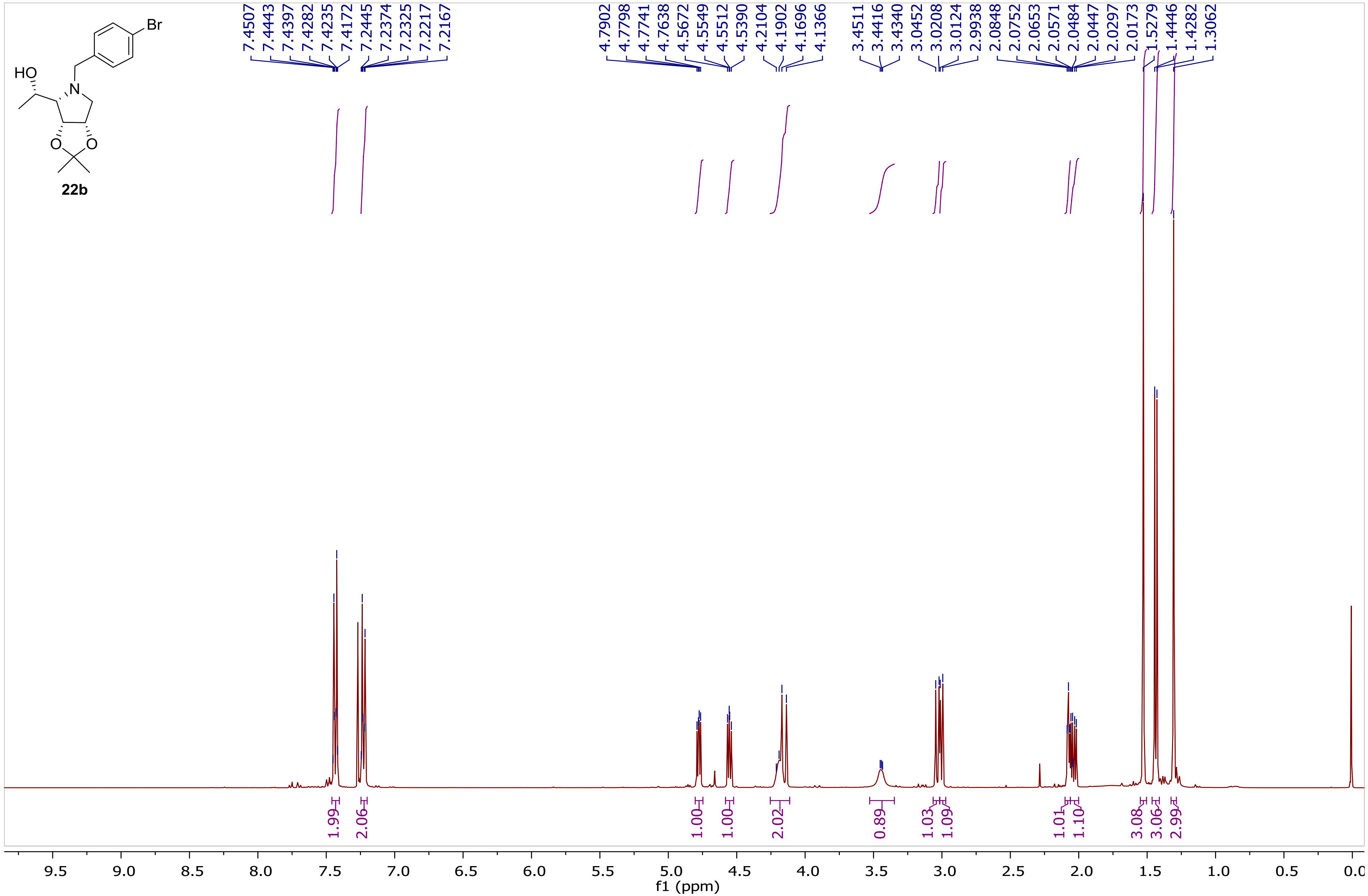
Br
HO
N
O
O
22b
7.4507
7.4443
7.4397
7.4282
7.4235
7.4172
7.2445
7.2374
7.2325
7.2217
7.2167
4.7902
4.7798
4.7741
4.7638
4.5672
4.5549
4.5512
4.5390
4.2104
4.1902
4.1696
4.1366
3.4511
3.4416
3.4340
3.0452
3.0208
3.0124
2.9938
2.0848
2.0752
2.0653
2.0571
2.0484
2.0447
2.0297
2.0173
1.5279
1.4446
1.4282
1.3062
1.99
2.06
1.00
1.00
2.02
0.89
1.03
1.09
1.01
1.10
3.08
3.06
2.99
f1 (ppm)

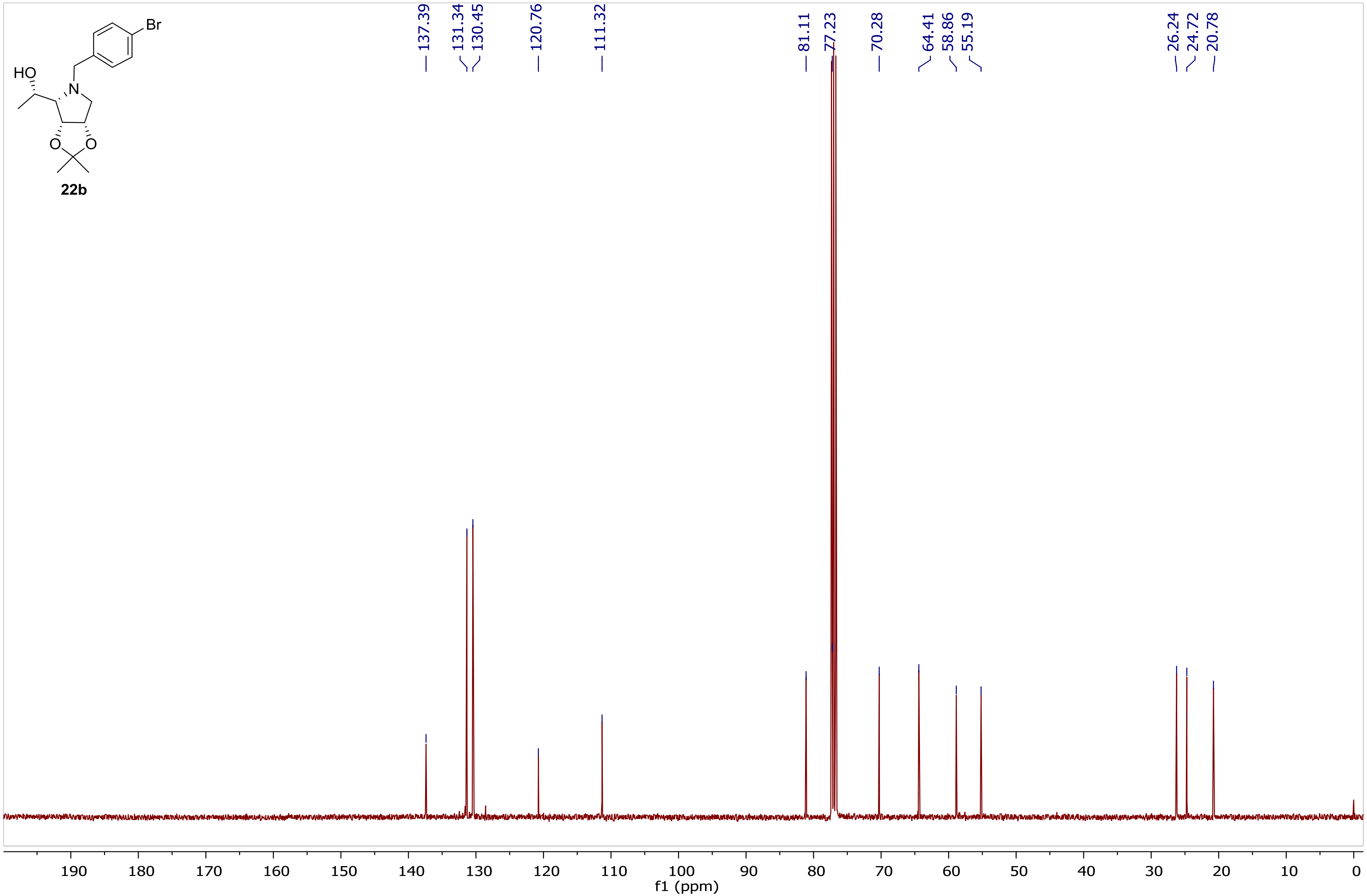
137.39
131.34
130.45
120.76
111.32
81.11
77.23
70.28
64.41
58.86
55.19
26.24
24.72
20.78
HO
N
Br
O
O
22b
190 180 170 160 150 140 130 120 110 100 90 80 70 60 50 40 30 20 10 0
f1 (ppm)

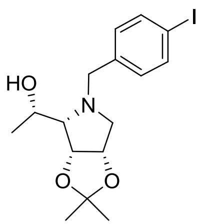
22c

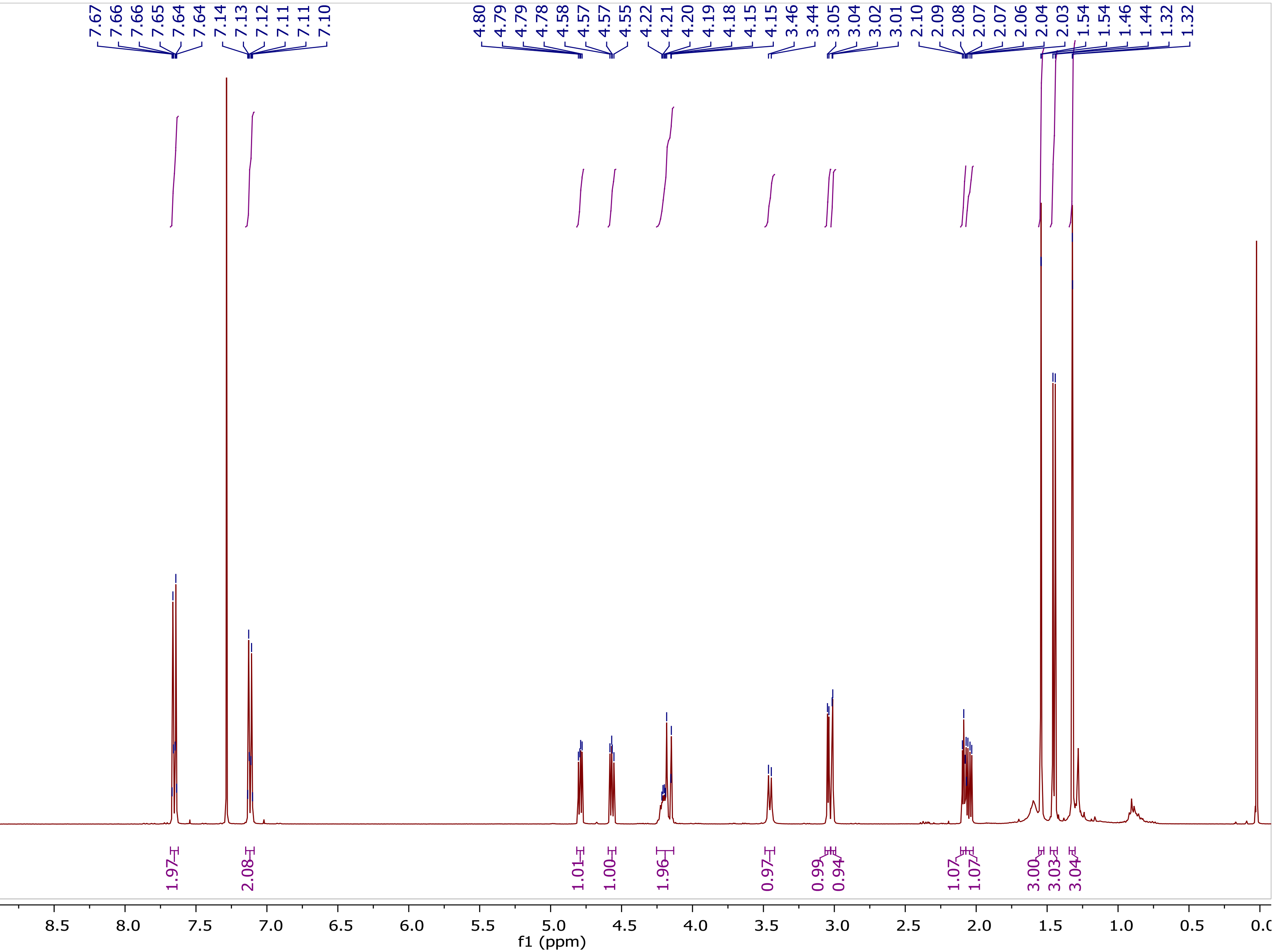

7.67
7.66
7.66
7.65
7.64
7.64
7.14
7.13
7.12
7.11
7.11
7.10
4.80
4.79
4.79
4.78
4.58
4.57
4.57
4.55
4.22
4.21
4.20
4.19
4.18
4.15
4.15
3.46
3.44
3.05
3.04
3.02
3.01
2.10
2.09
2.08
2.07
2.07
2.06
2.04
2.03
1.54
1.54
1.46
1.44
1.32
1.32
1.97
2.08
1.01
1.00
1.96
0.97
0.99
0.94
1.07
1.07
3.00
3.03
3.04
10.0
9.5
9.0
8.5
8.0
7.5
7.0
6.5
6.0
5.5
5.0
4.5
4.0
3.5
3.0
2.5
2.0
1.5
1.0
0.5
f1 (ppm)

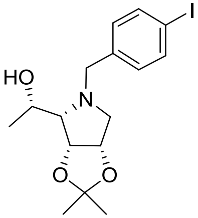

22c

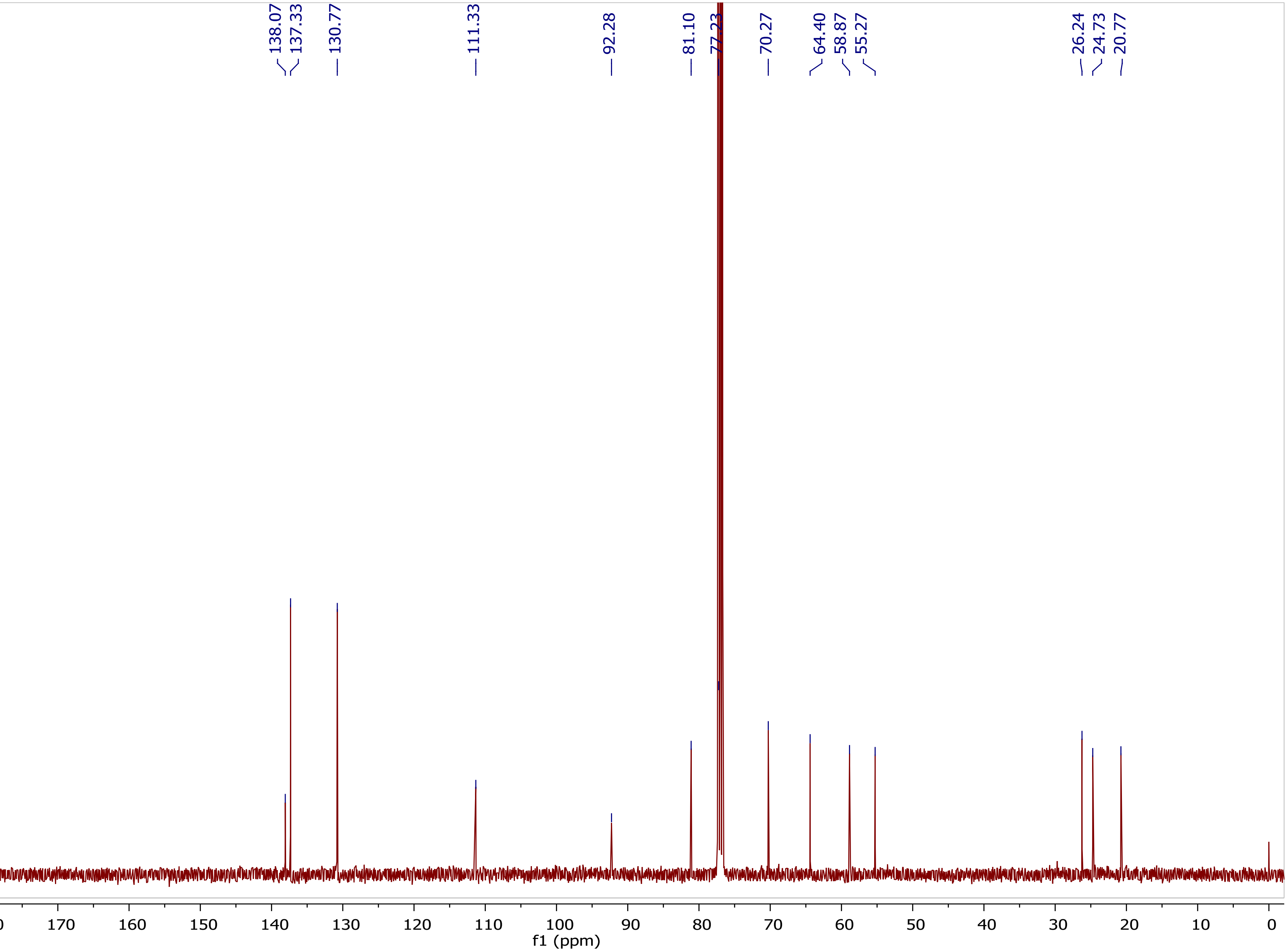

138.07
137.33
130.77
111.33
92.28
81.10
77.23
70.27
64.40
58.87
55.27
26.24
24.73
20.77
200
190
180
170
160
150
140
130
120
110
100
90
80
70
60
50
40
30
20
10
0
f1 (ppm)

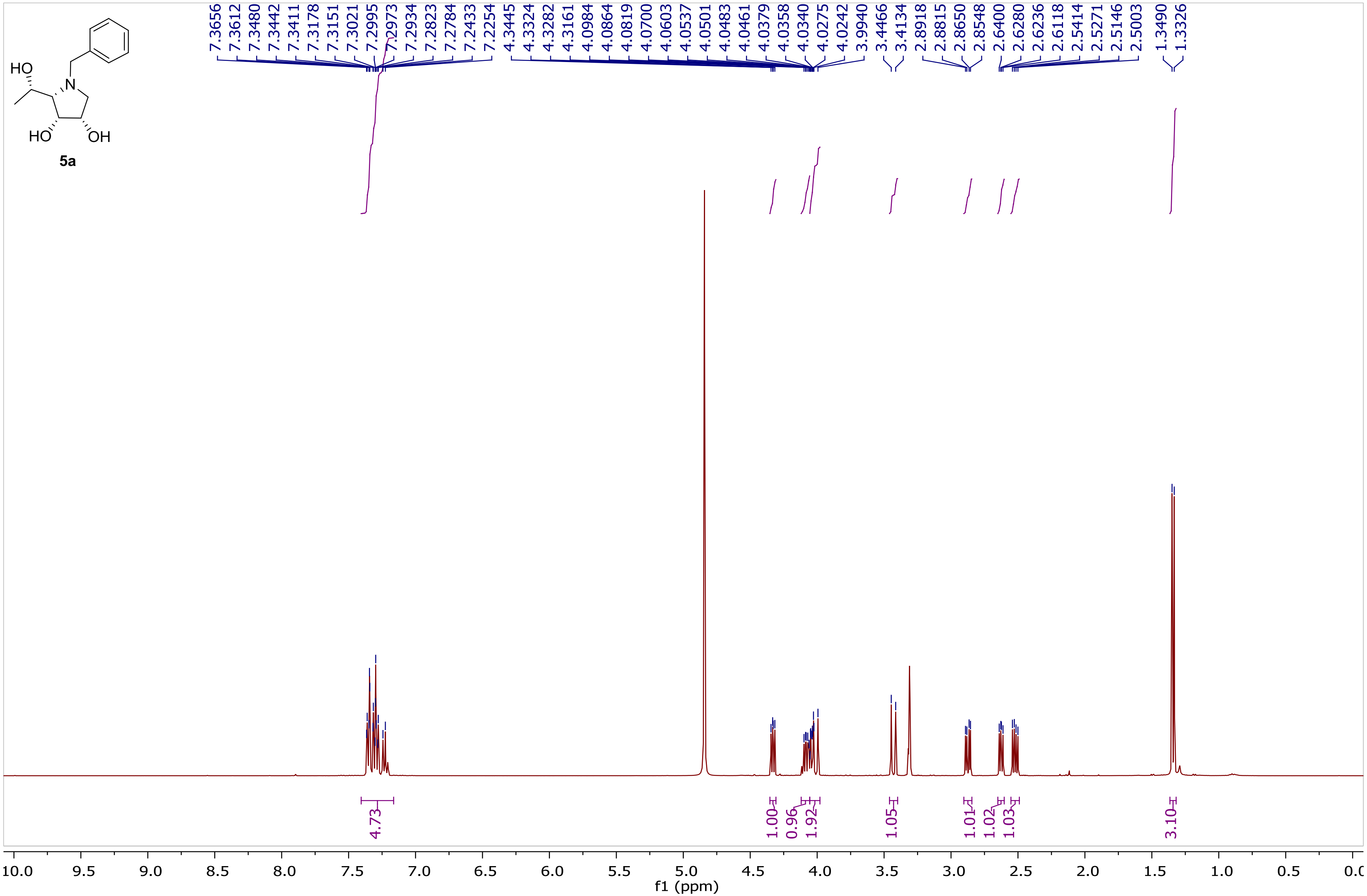
7.3656
7.3612
7.3480
7.3442
7.3411
7.3178
7.3151
7.3021
7.2995
7.2973
7.2934
7.2823
7.2784
7.2433
7.2254
4.3445
4.3324
4.3282
4.3161
4.0984
4.0864
4.0819
4.0700
4.0603
4.0537
4.0501
4.0483
4.0461
4.0379
4.0358
4.0340
4.0275
4.0242
3.9940
3.4466
3.4134
2.8918
2.8815
2.8650
2.8548
2.6400
2.6280
2.6236
2.6118
2.5414
2.5271
2.5146
2.5003
1.3490
1.3326
HO
N
HO
OH
4.73
1.00
0.96
1.92
1.05
1.01
1.02
1.03
3.10
f1 (ppm)

5a

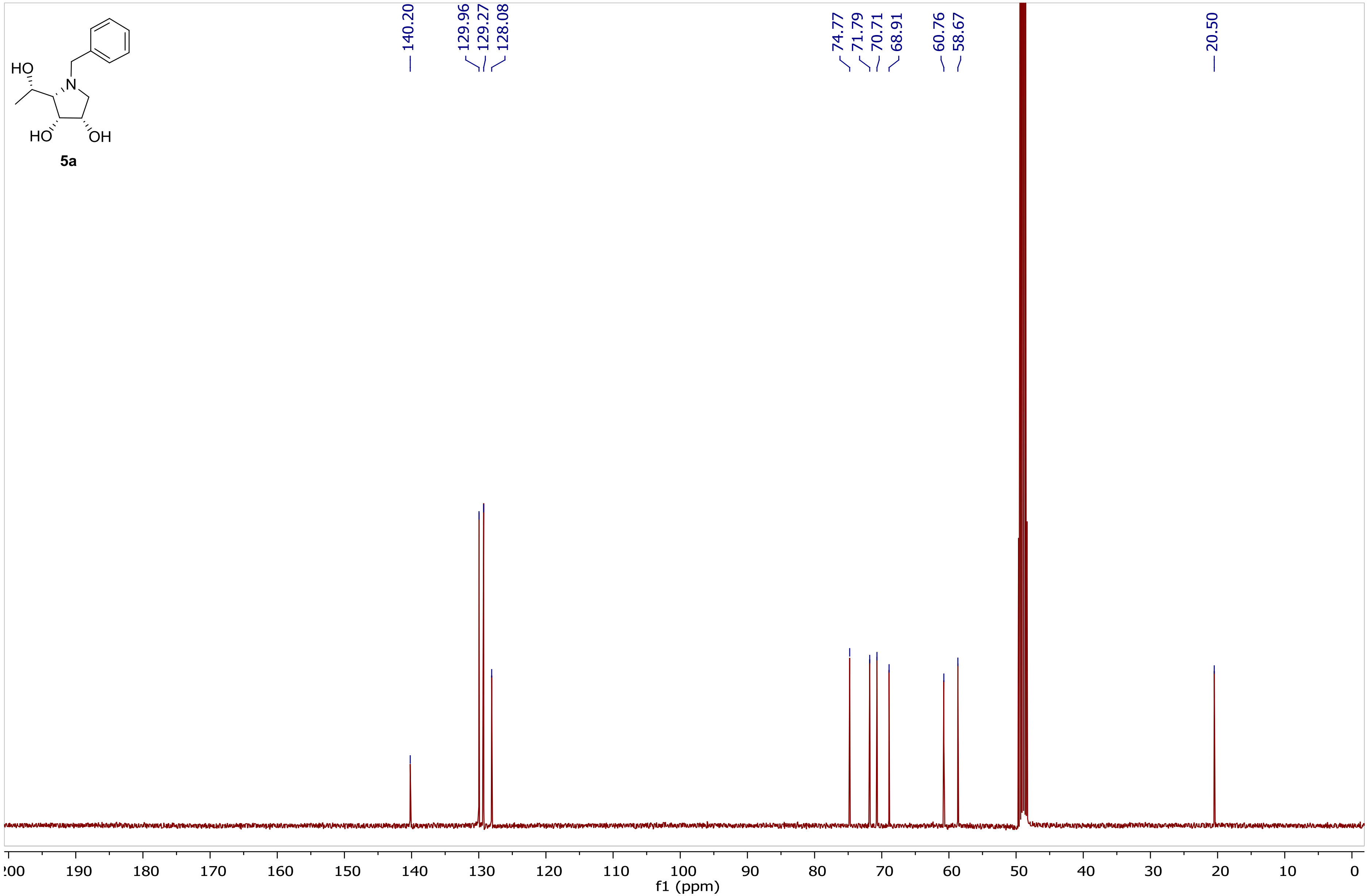

HO
N
HO
OH
5a
140.20
129.96
129.27
128.08
74.77
71.79
70.71
68.91
60.76
58.67
20.50
200
190
180
170
160
150
140
130
120
110
100
90
80
70
60
50
40
30
20
10
0
f1 (ppm)

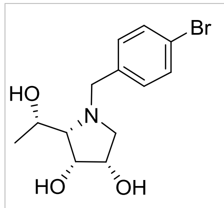
5b

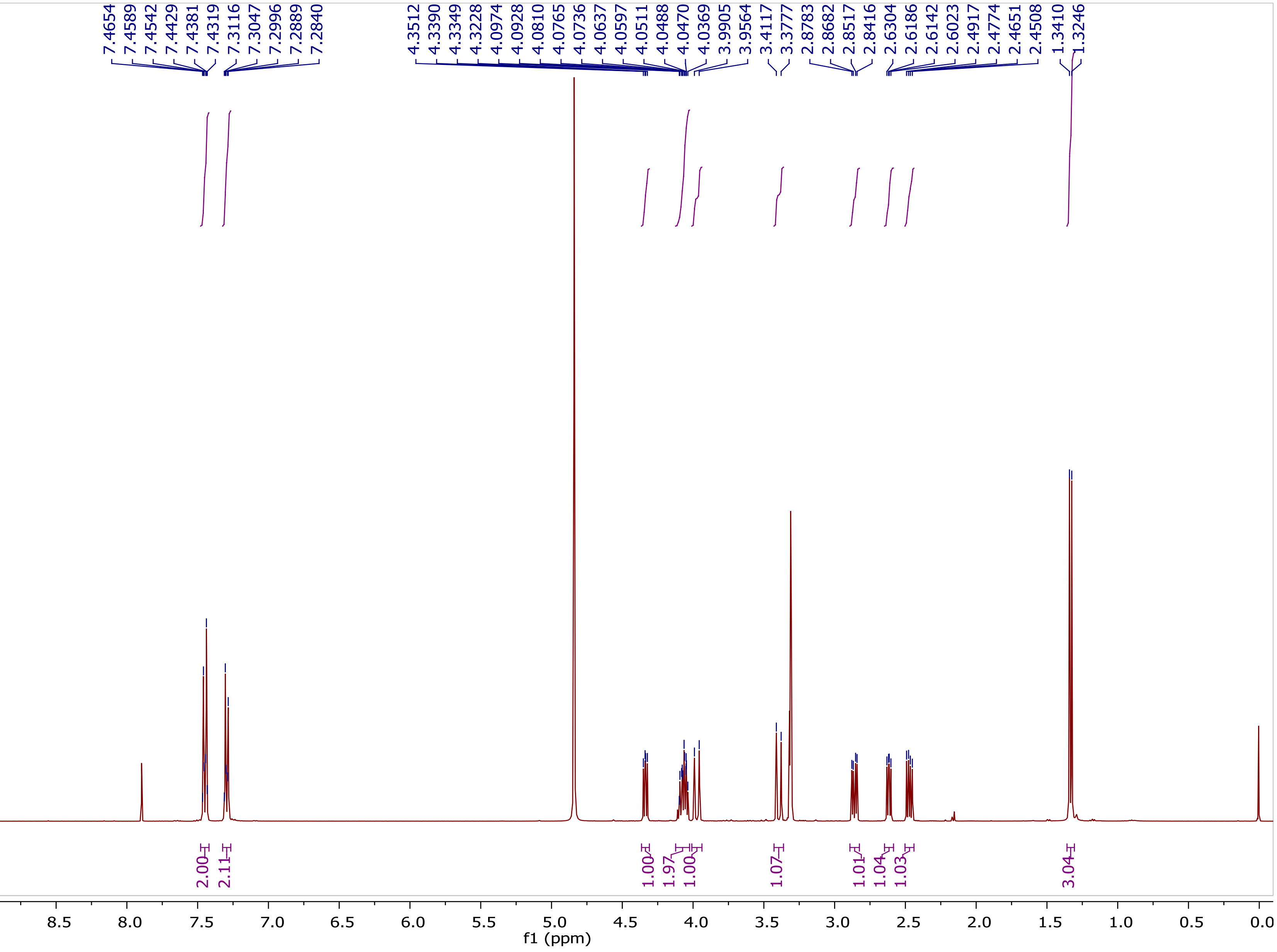
7.4654
7.4589
7.4542
7.4429
7.4381
7.4319
7.3116
7.3047
7.2996
7.2889
7.2840
4.3512
4.3390
4.3349
4.3228
4.0974
4.0928
4.0810
4.0765
4.0736
4.0637
4.0597
4.0511
4.0488
4.0470
4.0369
3.9905
3.9564
3.4117
3.3777
2.8783
2.8682
2.8517
2.8416
2.6304
2.6186
2.6142
2.6023
2.4917
2.4774
2.4651
2.4508
1.3410
1.3246
2.00
2.11
1.00
1.97
1.00
1.07
1.01
1.04
1.03
3.04
f1 (ppm)

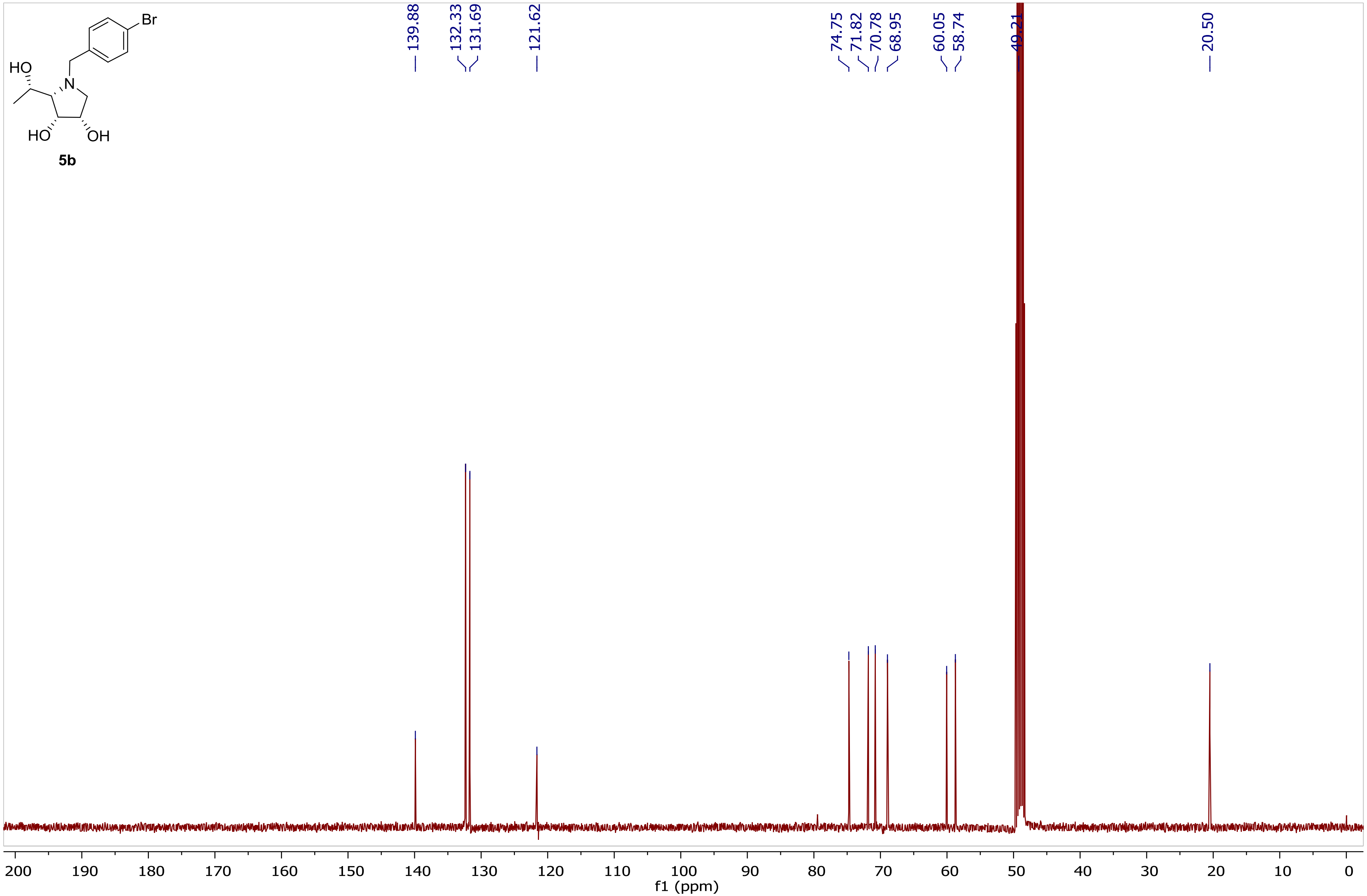

Br
HO
N
HO
OH
5b
139.88
132.33
131.69
121.62
74.75
71.82
70.78
68.95
60.05
58.74
49.21
20.50
200 190 180 170 160 150 140 130 120 110 100 90 80 70 60 50 40 30 20 10 0
f1 (ppm)

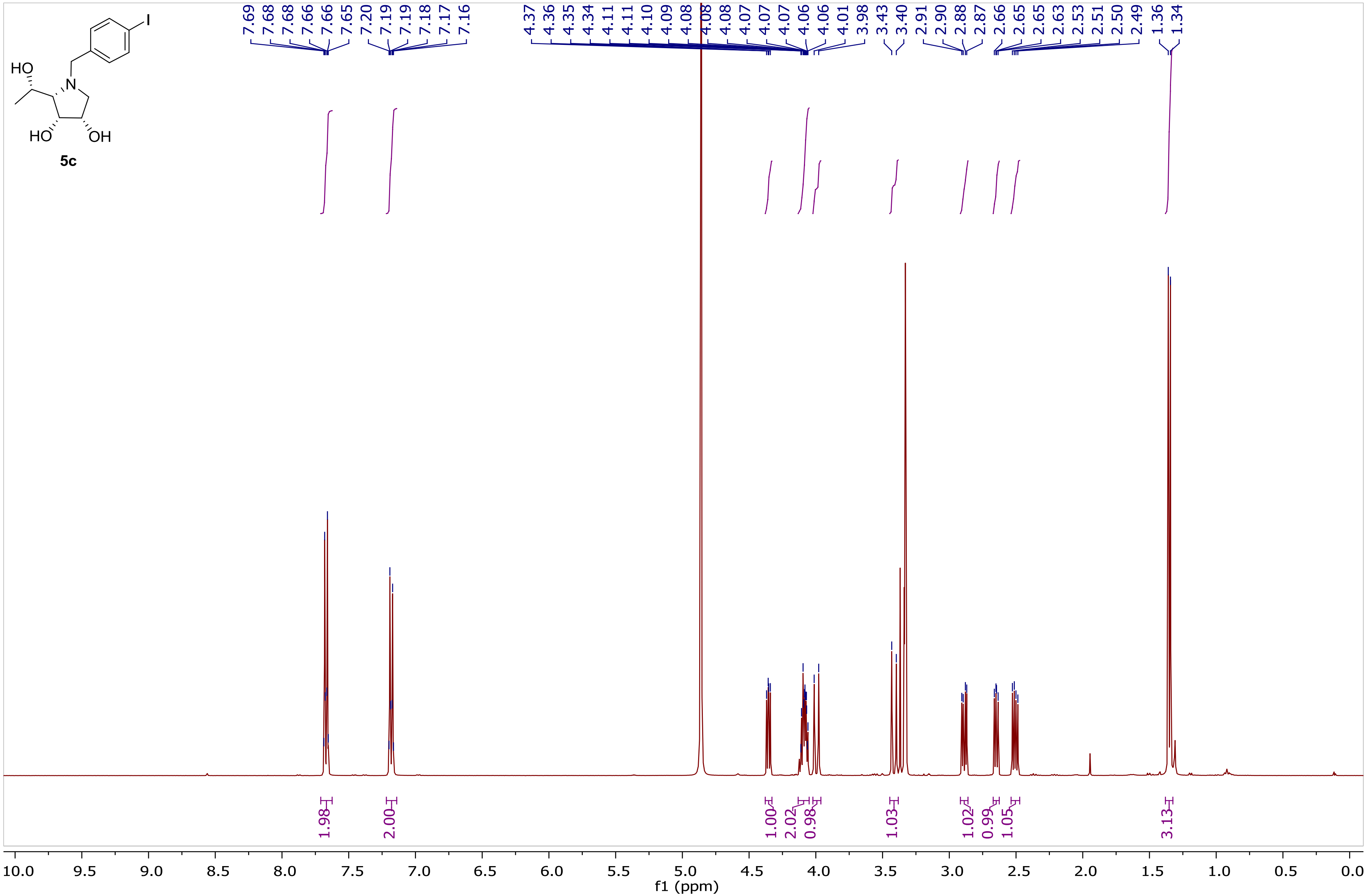
I
HO
N
HO
OH
5c
7.69 7.68 7.68 7.66 7.66 7.65 7.20 7.19 7.19 7.18 7.17 7.16
4.37 4.36 4.35 4.34 4.11 4.11 4.10 4.09 4.08 4.08 4.08 4.07 4.07 4.07 4.06 4.06 4.01 3.98 3.43 3.40
2.91 2.90 2.88 2.87 2.66 2.65 2.65 2.63 2.53 2.51 2.50 2.49 1.36 1.34
1.98 2.00 1.00 2.02 0.98 1.03 1.02 0.99 1.05 3.13
10.0 9.5 9.0 8.5 8.0 7.5 7.0 6.5 6.0 5.5 5.0 4.5 4.0 3.5 3.0 2.5 2.0 1.5 1.0 0.5 0.0
f1 (ppm)

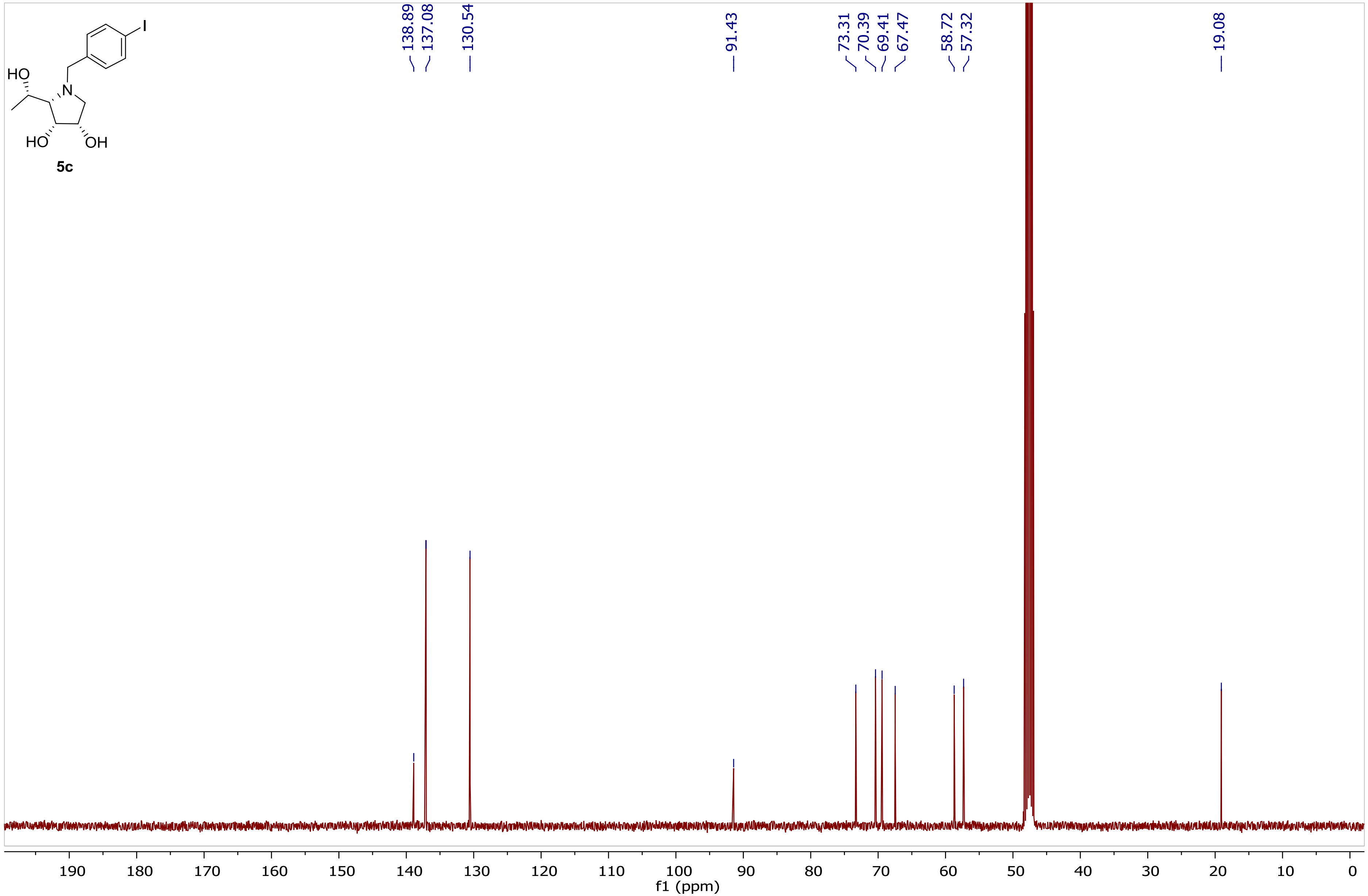

HO
N
I
HO
OH
5c
138.89
137.08
130.54
91.43
73.31
70.39
69.41
67.47
58.72
57.32
19.08
190 180 170 160 150 140 130 120 110 100 90 80 70 60 50 40 30 20 10 0
f1 (ppm)
